# Phenotyping Mediterranean Durum Wheat Landraces for Resistance to *Zymoseptoria tritici* in Tunisia

**DOI:** 10.3390/genes13020355

**Published:** 2022-02-16

**Authors:** Sarrah Ben M’Barek, Marwa Laribi, Hajer Kouki, Dalma Castillo, Chayma Araar, Meriem Nefzaoui, Karim Ammar, Carolina Saint-Pierre, Amor Hassine Yahyaoui

**Affiliations:** 1 Regional Field Crops Research Center of Beja (CRRGC), BP 350, Beja 9000, Tunisia; sarrah_bm@msn.com; 2 CRP-Wheat Septoria Precision Phenotyping Platform, Tunis 1082, Tunisia; mar.wa199@hotmail.fr (M.L.); koukihajercm@gmail.com (H.K.); chaymaaraar@hotmail.fr (C.A.); meriem.nef@gmail.com (M.N.); 3 National Agronomic Institute of Tunisia, University of Carthage, Tunis 1082, Tunisia; 4 CRI-Quilamapu, Instituto de Investigaciones Agropecuaria, Chillán 3780000, Chile; castillo.r.dalma@gmail.com; 5 Faculty of Sciences of Bizerte, University of Carthage, Jarzouna, Bizerte 7021, Tunisia; 6 Department of Agricultural and Food Sciences, Alma Mater Studiorum, University of Bologna, Via Zamboni 33, 40126 Bologna, Italy; 7 International Maize and Wheat Improvement Center (CIMMYT) km, 45 Carretera México-Veracruz El Batan, Texcoco CP56237, Mexico; k.ammar@cgiar.org (K.A.); c.saintpierre@cgiar.org (C.S.-P.)

**Keywords:** Mediterranean landraces, durum wheat, *Zymoseptoria tritici*, phenotyping, sources of resistance, diversity, seedling, adult, agronomic traits

## Abstract

Durum wheat landraces have huge potential for the identification of genetic factors valuable for improving resistance to biotic stresses. Tunisia is known as a hot spot for Septoria tritici blotch disease (STB), caused by the fungus *Zymoseptoria tritici* (*Z. tritici*). In this context, a collection of 3166 Mediterranean durum wheat landraces were evaluated at the seedling and adult stages for STB resistance in the 2016–2017 cropping season under field conditions in Kodia (Tunisia). Unadapted/susceptible accessions were eliminated to reach the final set of 1059 accessions; this was termed the Med-collection, which comprised accessions from 13 countries and was also screened in the 2018–2019 cropping season. The Med-collection showed high frequency of resistance reactions, among which over 50% showed an immune reaction (HR) at both seedling and adult growth stages. Interestingly, 92% of HR and R accessions maintained their resistance levels across the two years, confirming the highly significant correlation found between seedling- and adult-stage reactions. Plant Height was found to have a negative significant effect on adult-stage resistance, suggesting that either this trait can influence disease severity, or that it can be due to environmental/epidemiological factors. Accessions from Italy showed the highest variability, while those from Portugal, Spain and Tunisia showed the highest levels of resistance at both growth stages, suggesting that the latter accessions may harbor novel QTLs effective for STB resistance.

## 1. Introduction

Durum wheat is an important crop in the Mediterranean basin that has been cultivated over centuries and under widely variable climatic conditions. The crop originated and was domesticated in the Fertile Crescent (10,000 BP), and spread from the east to the west of the Mediterranean basin [1], reaching the Iberian Peninsula around 7000 years BP [2]. The diversification of the durum wheat genome and the development of a large collection of local populations in this region [3] are therefore mainly due to the contrasting environmental conditions. Moreover, multiple invasions that have occurred in the region, the migration of wheat from the east to the west of the Mediterranean basin, wheat imports, and natural and human selection are other important factors that contributed to its diversification (Mercer and Perales, 2010). This vast biodiversity within the species [4] also extends to the vast array of homemade foods derived from durum grains. Moreover, due to their high protein content and gluten strength, cultivars of durum wheat are preferred to produce semolina for use as pasta products, couscous, and bulgur [1,5]. Mediterranean durum wheat landraces have been, and continue to be, a great source of novel useful genes that could be further exploited by breeders.

Several studies have revealed the usefulness of landrace genetic resources, as they do offer a key element in breeding due to their inherent adaptability to respective agroecological niches [6,7,8] while maintaining considerable diversity between and within populations [9]. However, it is well known to breeders that useful genes are often linked to some undesirable traits that lead to longer cycles of selection to remove them. Landraces have been extensively characterized in terms of genetic diversity and population structure [3,10], and have great potential for the identification of novel sources of resistance to biotic and/or abiotic stresses [3,11,12,13,14]. Climate change does not only affect abiotic stresses such as drought, heat and cold, but also affects various fungal pathogens (rusts, leaf and head blights) [15] that have the potential to rapidly adapt to climate change; hence, it presents great limiting factors for wheat production in the Mediterranean region, which hosts some of the most damaging and virulent races of diseases and pests [16].

Septoria tritici blotch (STB), caused by the foliar fungal pathogen *Z. tritici*, is one of the most important threats to productivity in the Mediterranean basin, and particularly in Tunisia [17,18,19]. Ever since its emergence in 1970—which coincided with the introduction of the commonly high-yielding durum wheat varieties that became susceptible to Septoria over a few years [20]—Tunisia, and possibly other countries, experienced serious recurrent epidemics of STB, with yield losses reaching up to 40% [21]. Currently, in many countries, disease management relies on the use of fungicides and/or resistant cultivars. However, both fungicides and resistant cultivars are likely to lose efficacy against *Z. tritici* due to its high evolutionary potential [22]. Other factors—such as high seeding rate, early sowing, the excessive use of fertilizers, the slow release of resistant varieties, a lack of variety replacement, scarce crop rotations, a shift to reduced/no tillage agriculture, and the excessive use of fungicides—are major constraints limiting more efficient approaches to managing STB in Tunisia. In addition, cereal crops are dominated by a monoculture of genetically uniform wheat cultivars over very large areas. In Tunisia, durum wheat variety “Karim” (Jori“S”/Anhinga“S”//Flamingo“S”), which is highly susceptible to *Z. tritici*, covers more than 60% of the durum wheat acreage [23,24,25].

Despite all of the research undertaken, STB is still a major durum disease in the Mediterranean region, and with the pressure of Septoria becoming insensitive to some fungicides [26], demand for new Septoria resistant wheat varieties by farmers has increased. Moreover, the high genetic variability of *Z. tritici* that is mainly driven by sexual reproduction [27,28,29,30,31] allows not only the natural increase in inoculum density, but also new combinations of virulence alleles. Consequently, it seems likely that most resistance genes will not last long, and there will be a continual need to identify new sources of resistance.

Breeding for resistance is therefore an ongoing process that requires new resistance alleles to be incorporated into crop varieties, which must be managed strategically. This underscores the need for crop genetic-resource conservation and the need to systematically test wild relatives, landraces, and other germplasms, to identify new genetic sources of resistance to major diseases [32,33,34]. Recent studies have shown that Tunisian durum wheat landraces carry effective sources for resistance to *Z. tritici* [35,36,37].

The objectives of this study were to evaluate a sub-set of Mediterranean durum wheat landraces, mainly originating from Algeria, France, Italy, Portugal, Spain and Tunisia, for resistance to *Z. tritici* under field conditions at seedling and adult stages. The relationship between disease development and plant height (PH) was also evaluated. The integrated field seedling and adult plant phenotyping method reported in this study provides a great tool for identifying novel and durable resistance sources. Our method has the advantage of assessing the populations over a long growing period (GS11–87) [38] under the same field conditions for two years. Assessing plants at early and various growth stages can enable selection for desirable gene combinations. Moreover, the outcome of the present study will help in the identification of landraces that can be exploited for improving resistance to *Z. tritici* in Tunisia.

## 2. Materials and Methods

### 2.1. Plant Material, Experimental Design and Inoculation Method

**Figure 1:**
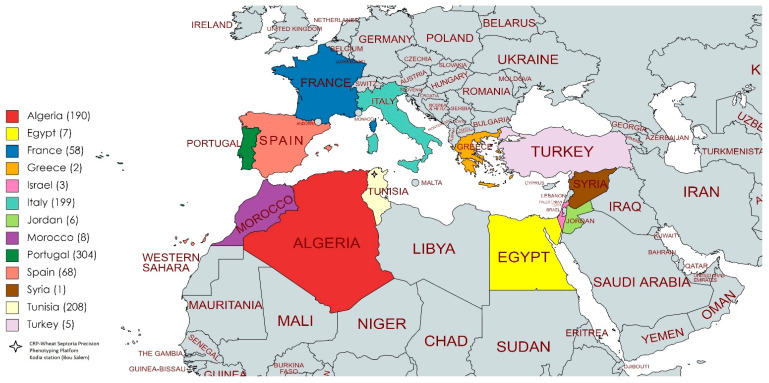
Origin of 1059 accessions tested over 2-year trials (2016–2017 and 2018–2019) at the CRP Wheat Septoria Phenotyping Platform, experimental station of Kodia (Bou Salem, Tunisia).

A collection of 3166 durum wheat accessions, provided by the USDA Aberdeen Gene Bank, including accessions from 17 different countries (Algeria (214), Palestine (3), Cyprus (44), Egypt (163), France (79), Greece (67), Israel (28), Italy (275), Jordan (56), Lebanon (11), Morocco (174), Portugal (389), Spain (128), Switzerland (2), Syria (45), Tunisia (299) and Turkey (1189)) were tested for resistance to *Z. tritici* under field conditions at the CRP Wheat Septoria Precision-Phenotyping Platform of the experimental station located at Kodia (36°32′51.89 N, 9°0′40.73 E)-INGC (Bou Salem, Tunisia), during the cropping season of 2016–2017. These materials were at different improvement statuses, including landraces (2352), cultivars (234), genetic material (15), breeding material (220) and unknown improvement status (345). Only 1059 (33.5%) of these accessions were retained and tested during the 2017–2019 cropping seasons for Septoria tritici blotch (STB) resistance (Supplementary Table S1). The remaining 2107 accessions (66.5%) were unadapted, as they were either winter type, highly susceptible to yellow rust, or failed to germinate, and were thus not considered for further analysis. The 1059 accessions, mostly composed of landraces (66%), were then screened for STB resistance/tolerance at both seedling and adult growth stages, at the location that is known as a hot-spot region for Septoria. These accessions were from Algeria (190), Egypt (7), France (58), Greece (2), Israel (3), Italy (199), Jordan (6), Morocco (8), Portugal (304), Spain (68), Syria (1), Tunisia (208) and Turkey (5) (Figure 1). All accessions were planted on 16 November 2016 and 13 November 2018, in a wheat-after-wheat production system. An augmented experimental design with unreplicated entries and replicated checks was implemented during both 2-year trials. The plots consisted of two rows of 1 m in length. The spacing between the plots and blocks was 0.5 m and 1 m, respectively. Each block contained three local checks—‘Karim’, ‘Nasr’ and ‘Salim’—known for their susceptibility, moderate susceptibility, and resistance to STB disease, respectively. These checks were sown in two rows and replicated among blocks, with a total of 30 replicates per check. The average of these replicates was used to classify the different accessions based on their levels of resistance/susceptibility (Supplementary Table S2). The replicated checks were also used as a tool to verify the uniformity of the infection among and within plots. The susceptible variety, ‘Karim’, was also planted in the middle of the block, and served as a disease spreader to further induce infection and ensure optimal disease distribution among and within plots.

Straw from the previous cropping seasons was incorporated into the soil with a rotary harrow. The inoculum density was approximately 200 g·m^−2^. Additional, straw inoculations were performed when the tested material reached the growth stage GS10 [38]. These inoculations were conducted by evenly spreading freshly cut infected wheat-straw over the experimental plots and disease spreader rows, using the susceptible cultivar ‘Karim’ to ensure optimal disease development. Moreover, to maintain a high level of disease pressure, artificial infection was performed. Five *Z. tritici* strains (TU16318, TU16323, TU16344, TU16363, TU16364), which were collected from the same region in 2016 and are known for their virulence, were chosen to prepare the inoculum. The inoculum concentration was adjusted to 1 × 10^6^ spores·mL^−1^ amended with 0.1% Tween 20 surfactant (polyoxyethylenesorbitan monolaurate) (Merck, Watford, UK), to reduce surface tension. Wheat plants were inoculated after sunset using a sprayer (Efco AT800, Reggio Emilia, Italy) three to four times prior to stem elongation at the tillering stage (from GS11 to GS29) [38]. Irrigation was applied as needed to ensure favorable conditions for STB development and standard wheat agronomic practices were applied during the 2016–2017 and 2018–2019 crop seasons.

Moreover, plant height was measured for all 1059 accessions during the two-year trials at maturity, for each plot, from ground level up to the extreme of the spike (including awns).

### 2.2. Disease Rating

The Mediterranean collection of 1059 accessions, hereafter called the Med-collection, was subsequently evaluated for STB resistance under field conditions. To better discriminate resistance at seedling and adult growth stages under field conditions, accessions were rated at GS (11–20) and GS (37–87) stages, respectively. At the seedling stage, a 0 to 5 scale was implemented (Table 1), where scores between 0–1 indicate an immune to highly resistant (HR) response. Scores equal to 2 and 3 indicate resistant (R) and moderately resistant (MR), respectively, while scores of 4 and 5 correspond to moderately susceptible (MS) and susceptible (S), respectively (Table 1 and Table S1).

At the adult stage, STB progression was evaluated by measuring STB incidence and severity based on the double-digit scale (00–99) [20] where the first digit indicates disease incidence on the infected plants, and the second digit refers to the severity of infection (Table 2) by evaluating the pycnidia coverage. Accessions were rated over multiple consecutive times, starting with GS37, at 10-day intervals [38].

Hence, the symptoms and lesion development over the assessment period were summarized by the area under disease progress curve (AUDPC), which allowed the identification of different classes of resistance. The area under disease-progress curve (AUDPC) and the relative area under diseaseprogress curve (rAUDPC) were determined according to [39];

$$\mathrm{AUDPC}=\overset{n-1}{\underset{i=1}{\sum }}\frac{y_{i}+y_{i+1}}{2}\times \left(t_{i+1}-t_{i}\right)$$

where:

y_i_: STB severity at time t_i_,

t_(i+1)_ − t_i_ = time interval (days) between two disease scores,

n = number of times when STB was recorded.

$$\mathrm{rAUDPC}=\frac{\mathrm{AUDPC}\;\left(\mathrm{genotype}\right)}{\mathrm{AUDPC}\;\left(\mathrm{Karim}\right)}$$

where: Karim is the susceptible check of the corresponding trial.

Based on the levels of resistance and susceptibility of the local checks Salim, Nasr and Karim of each trial, five classes were established at adult growth stage (Table 2).

The relative area under disease progress curve (rAUDPC) was calculated for all 1059 accessions over the 2-year trials using the AUDPC of the susceptible check ‘Karim’ for each corresponding cropping season. The rAUDPC of both ‘Salim’ and ‘Karim’ (Table 2 and Table S2), were ranked as highly resistant (HR), resistant (R), moderately resistant (MR), moderately susceptible (MS) and susceptible (S) (Table 2 and Table S1).

### 2.3. Statistical Analysis

R software version 4.1.2 (R Foundation for Statistical Computing (R Core Team (2021)) [40] was used for all data analysis. Principal Component Analysis (PCA) was performed on the parameters PH, AUDPC and rAUDPC over the two testing seasons using the R package ‘MASS’ [41]. The determination and visualization of clusters was performed using R packages ‘factoextra’, ‘cluster’ and ‘stats’ [40,42,43]. The coefficient of the correlation between variables (seedling and adult reaction, PH) was determined with ‘cor.test’ function from the R package ‘stats’. The analysis of variance (ANOVA) was performed with the ‘aov’ function from the R package ‘stats’ [40].

## 3. Results

### 3.1. Disease Response of the USDA Mediterreanean Collection

The USDA Mediterranean collection showed a high frequency of resistance (HR and R = 83.9%), among which 58.5% showed an immune reaction (HR) at adult growth stage (Table 3). The absence of typical symptoms of *Z. tritici* in these accessions could be attributed to a major-gene resistance or disease-escape traits, notably height.

A set of the USDA Mediterranean accessions (33.5%) was kept for the 2018–2019 cropping season, and comprised 1059 accessions that we named the Med-collection. The latter was composed of landraces (66%), breeding lines (12%), unknown improvement status (11%), cultivars (9%) and genetic material (<2%). The accessions were from major durum-wheat-growing countries where Septoria is a major disease of durum wheat, including Algeria, France, Egypt, Italy, Portugal, Spain, Tunisia, Greece, Israel, Morocco, Syria, Jordan, and Turkey. Countries represented by low numbers of accessions (≤8) were grouped together and named Other.

Data on disease evaluation based on seedling and adult scores (AUDPC and rAUDPC) under field conditions of the Med-collection, as well as phenology data such as plant height, over two seasons of the experiment are shown in Supplementary Table S1.

### 3.2. Reactions of Genotypes across the Two Trials

**Figure 2:**
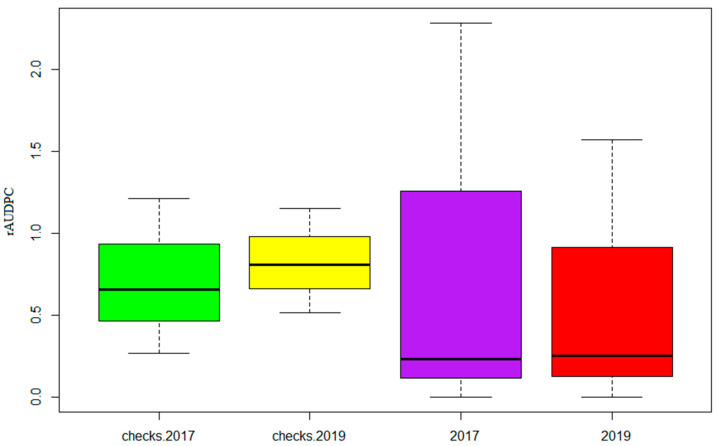
Box plots of the relative area under the disease-progress curve (rAUDPC) of the Med-collection and the checks inoculated with *Z. tritici* under field conditions in the 2016–2017 and 2018–2019 cropping seasons.

The comparison of the mean rAUDPC and standard deviation of all of the checks used in this study are represented in Supplementary Table S2 and Figure 2.

Optimal rainfall along with the artificial inoculations allowed good environmental conditions that were conducive to the development of Septoria among the genotypes and the checks during both seasons. Nevertheless, a higher disease pressure for the evaluated accessions was observed in 2017 (Figure 2). The combined analysis of variance showed no significant difference at seeding stage (*p* < 1) and a moderate significant difference at the adult stage (*p* < 0.01) between years 2017 and 2019, with regard to the disease progress (Figure 2, Table 4). At the seedling and adult stages, the genotype term in the ANOVA analysis was highly significant at *p* ≤ 0.001, which confirms that the observed variation was mainly due to the contribution of the variable genetic background of the tested germplasm. Conversely, there was no significant genotype–year interaction, indicating that genotypes behaved similarly between years (Table 4).

**Figure 3:**
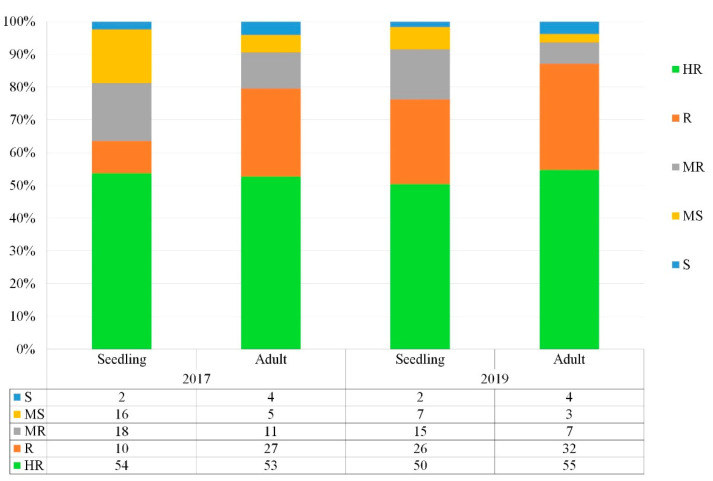
Bar graph showing the response of the core collection against *Z. tritici* at seedling and adult growth stages under field conditions for two cropping seasons (2016–2017 and 2018–2019). The X-axis represents the type of resistance found in accessions comprising highly resistant (HR), resistant (R), moderately resistant (MR), moderately susceptible (MS), and susceptible (S) accessions. Values on the bar represent frequency of landraces (%) based on their level of resistance/susceptibility.

The Med-collection showed a diverse response to STB (Supplementary Table S1) under field conditions at both seedling and adult stages, exhibiting reactions that ranged from susceptible (S) to highly resistant (HR) (Figure 3). Results showed that over 50% of the accessions had immune to highly resistant reactions (Figure 3), and around 30% had good resistance levels. Accessions in this category could be used as source of resistance in breeding programs. About 7% of the accessions in 2019 were susceptible types that should not be considered for exploitation by breeding programs. Interestingly, 92% of HR and R accessions maintained their resistance levels across the two years.

The Pearson’s correlation coefficient (*r*) values between two repeated experiments was highly significant, with 0.51 and 0.43 for adult–adult and seedling–seedling reactions for both years, respectively (Table 5). Similarly, the correlation between seedling and adult reactions was highly significant for both years (Pearson’s correlation *r* = 0.597, *p* ≤ 0.001; *r* = 0.455, *p* ≤ 0.001, respectively).

### 3.3. Association between Disease Parameters and Plant Height

**Figure 4:**
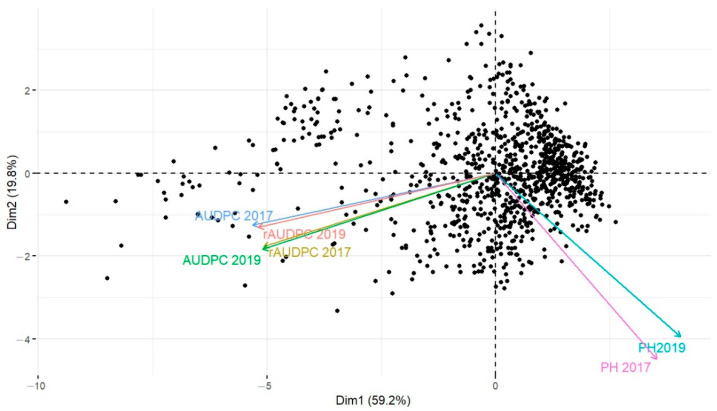
Principal Component Analysis (PCA) showing a significant correlation between AUDPC and rAUDPC, and a negative correlation between these parameters and Plant height of the Med-collection over two years.

In addition to the disease response, plant height (PH) was recorded to identify any association to STB disease. A significant variation within the Med-collection in relation to this trait was observed (Supplementary Table S1) with PH ranging from 60 to 195 cm. The PCA was performed to further understand the relative importance and contribution of the PH to the overall disease development. The results showed two dimensions of PCA explaining 79% of data variance (Figure 4). The first dimension accounted for 59.2% of the variances, while the second dimension accounted for 19.8% of variances. Among the disease parameters (rAUDPC), a highly significant correlation was observed between both years (*r* = 0.511; *p* ≤ 0.001). Among the agronomic trait, negative values for the Pearson’s correlation coefficient were observed with respect to rAUDPC. The Pearson’s correlation coefficients with rAUDPC were −0.263 (*p* ≤ 0.001) and −0.407 for plant height, respectively, in 2017 and 2019 (*p* ≤ 0.001) (Table 5). This indicated that over the two cropping seasons, PH had a negative effect on the severity of STB disease. In addition, it also showed that the shorter the plant, the higher the STB infection.

**Figure 5:**
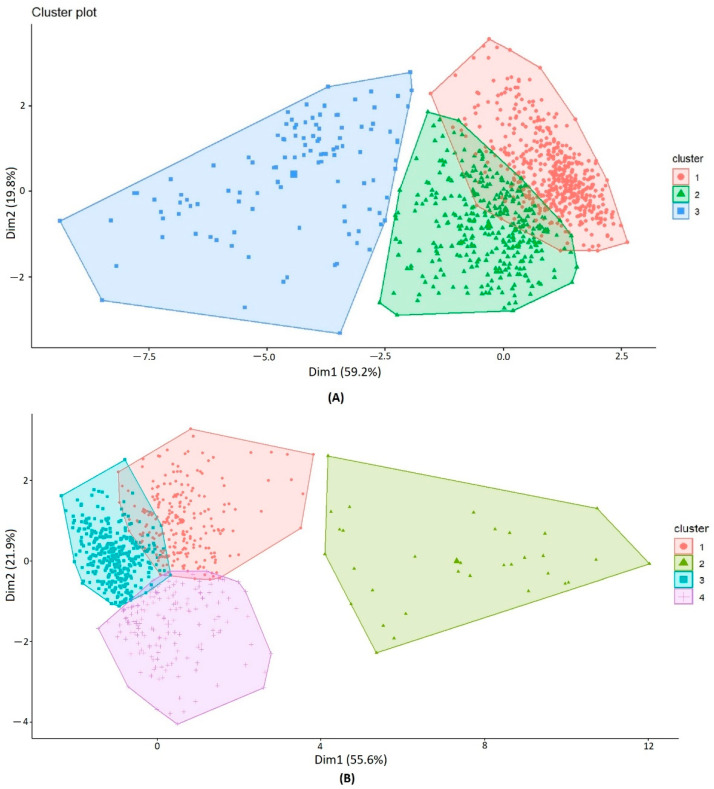
(**A**) Clustering of the Med-collection representing the contribution of three factors: plant height (PH), the area under disease progression curve (AUDPC) and the relative area under disease-progression curve (rAUDPC), seasons 2017 and 2019. Seven accessions were not assigned in any of the clusters; (**B**) clustering of the Med-collection and representation of the contribution of three factors: plant height (PH), the area under disease progression curve (AUDPC) and the relative area under disease progression curve (rAUDPC), seasons 2017 and 2019. This clustering was conducted after eliminating the accessions with moderately susceptible and moderately resistant reactions. One accession was not assigned in any of the clusters.

Clustering analysis revealed three main clusters (Figure 5A). Cluster 1 (red), which is the largest cluster comprised 579 accessions; cluster 2 (green), consisting of 359 accessions; and finally, cluster 3 (blue), containing114 accessions. Cluster 1 contained mostly 98% of HR and R genotypes (#571 accessions) and had PH values that ranged from 65 to 175 cm. Interestingly, 86% of 571 accessions, mostly composed of landraces, were classified as very tall and had PH values ranging from 110 to 175 cm. These accessions were mainly represented by genotypes from Portugal, Tunisia, Italy, Spain and Algeria. Cluster 2 displayed accessions with various reactions, with the majority (#60%) having HR and R reactions.

To further differentiate HR and R accessions from the susceptible, we eliminated the accessions with MS and MR reactions and reconducted a cluster analysis. In total, 831 accessions were clustered into four different clusters (Figure 5B). Results showed two dimensions explaining 77.5% of data variance. The first dimension accounted for 55.6% of the variance, while the second dimension accounted for 21.9% of the variance. Cluster 1 (red) comprised 228 accessions and cluster 2 (green) contained 32 accessions, while cluster 3 (blue) was the largest cluster and comprised 397 accessions. Finally, cluster 4 (purple) contained 173 accessions. Cluster 3 contained HR and R genotypes that were mostly very tall (PH ranging from 110 to 170 cm) (#99%). Cluster 1 also contained mostly HR and R genotypes that were rather tall, although with a predominance of R genotypes. Interestingly, cluster 4 contained genotypes with HR and R reactions, but with a predominance of HR and displaying different heights (PH = 65–150 cm). Within this cluster, 30% of genotypes classified as short (PH = 70–110 cm) were mostly composed of breeding material and landraces, and originated mainly from France, Italy and Portugal. In addition, 45% of genotypes had intermediate heights (PH = 110–130 cm). Finally, cluster 2 mostly contained susceptible genotypes. The clustering was not dependent on the country of origin or the status of improvement of the accessions.

### 3.4. Geographical Distribution of the Resistant and Susceptible Landraces

**Figure 6:**
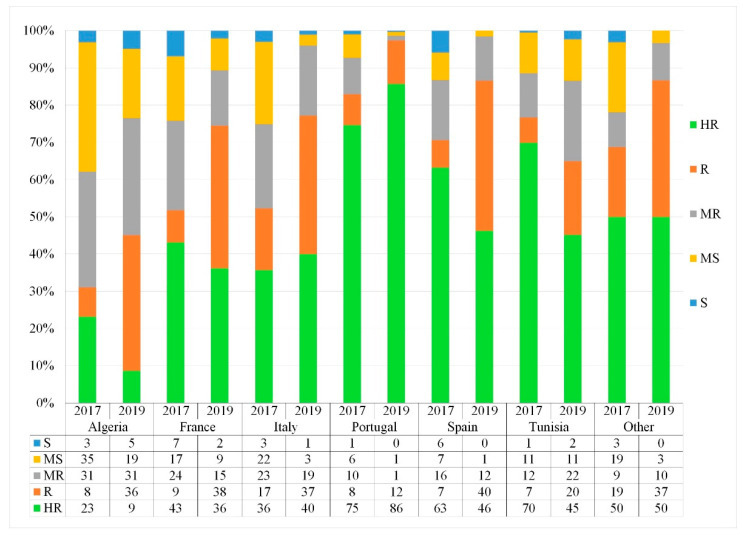
Seedling-stage frequency distribution (%) of disease response of the Med-collection based on the country of origin. HR: highly resistant; R: resistant; MR: moderately resistant; MS: moderately susceptible; S: susceptible.

**Figure 7:**
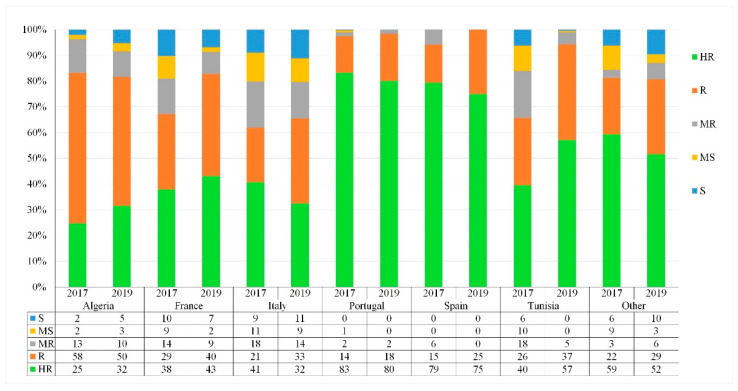
Adult growth-stage frequency distribution (%) of disease response of the Med-collection based on the country of origin. HR: highly resistant; R: resistant; MR: moderately resistant; MS: moderately susceptible; S: susceptible.

High diversity within and between the countries of origin was observed in resistance levels to *Z. tritici*, as well as the phenological traits measured. The frequency of resistance level varied between years and countries of origin (Figure 6 and Figure 7).

At the seedling stage, accessions from Portugal showed a high frequency of high resistance levels and a slight change between years. Accessions from Spain and Tunisia did have good levels of high resistant lines, but also showed lower frequency changes between years. Over 30% of accessions from France and Italy showed good resistance levels (HR) over the 2-year period, and resistance levels were maintained over the testing period. Algerian, Tunisian, and Italian populations were highly variable, even though the population showed a slight shift from HR to R (Figure 6).

At the adult stage, nearly the same trend was observed with accessions from Portugal and Spain, showing a high frequency of high resistance levels and a slight change between years, thus having the lowest variability within the populations. Accessions from Tunisia did have good levels of highly resistant lines, but also showed slight frequency changes between years. Nevertheless, accessions from France, Italy and Algeria were highly variable, and showed a lower frequency of HR accessions over the 2 years with a slight shift from HR to R compared to the other countries, though resistance levels were maintained over the testing period (Figure 7). Overall, the distribution of the resistant accessions varied between their geographical origin.

The moderately susceptible and susceptible accessions mostly originated from Italy. Among the tested populations, accessions from Algeria and Italy were the most variable, encompassing resistant, intermediate, and susceptible accessions.

Although a strong correlation of disease severity between seedling and adult stages was observed, we did notice some variability between countries. For instance, accessions from Algeria showed variability in their disease response from seeding to adult, while accessions from France and Portugal maintained relatively the same reaction.

### 3.5. Distribution of the Reaction Types among and between Populations

**Figure 8:**
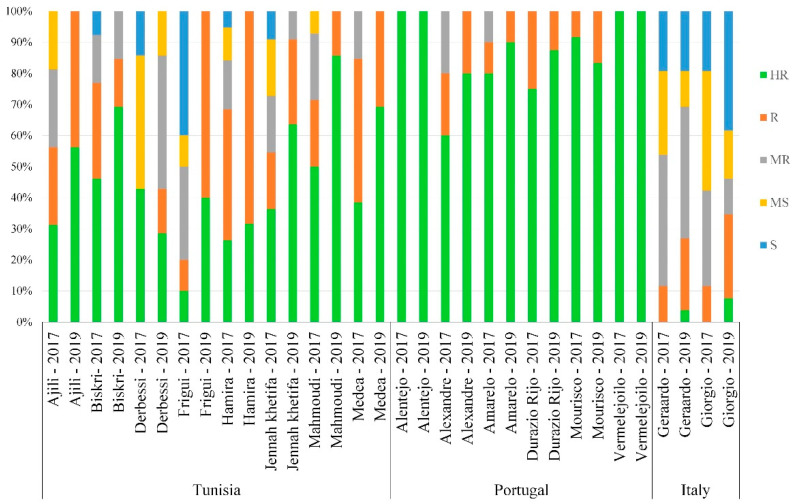
Disease reaction within lead population/country in both years.

Landrace populations within a country with several accessions known by their PI/Cltr numbers—a USDA reference identifier—were compared in this study. The distribution of resistance at the adult plant stage was not correlated with the geographical distribution of the landrace populations (Figure 8), even though some showed no significant differences in plant height and maturity.

As shown in Figure 8, Portuguese populations (Alentejo, Vermelejoilo) had the highest HR values, followed by the populations of Alexander, Amarelo, Durazio Rijo and Mourisco from Portugal, and the populations of Ajili and Mahmoudi from Tunisia. However, Italian populations (Gerardo and Giorgio) showed low levels of resistance in both trials. Portuguese populations had the lowest variability in resistance levels whereas Tunisian and Italian populations had a relatively high variation between and within populations and between years. Tunisian populations (Ajili, Biskri, Hamira, Jenah Khetifa, Mahmoudi, and Medea) (Figure 8) apparently had good resistance levels (HR, R, MR), which could be exploited in the Mediterranean region (Algeria, Portugal, France, Italy, Spain, Tunisia and neighboring countries).

**Figure 9:**
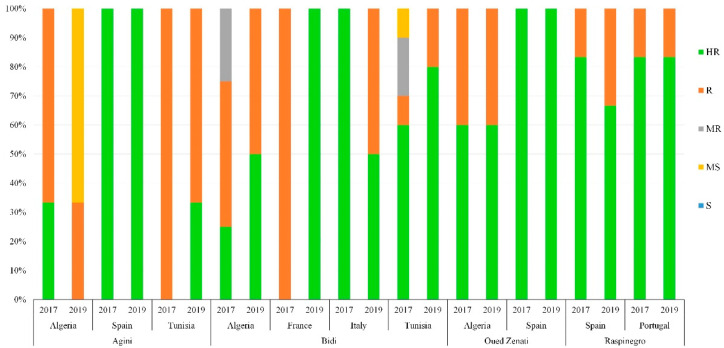
Disease reaction within same population originating from different countries in both years. HR: highly resistant; R: resistant; MR: moderately resistant; MS: moderately susceptible; S: susceptible.

In addition, populations with the same common name but originating from different agroecological regions—such as Ajini from Algeria, Spain and Tunisia; Bidi from Algeria, France, Italy and Tunisia; Oued Zenati from Algeria and Spain; and Raspinegro from Spain and Portugal—were assessed in this study. Interestingly, populations of Raspinegro were highly resistant to disease, independently of their origin (Spain and Portugal) (Figure 9).

## 4. Discussion

Breeding for disease resistance has been one of the important traits in breeding programs worldwide; thus, finding reliable sources of adult-plant resistance is of great importance to breeders. Durum wheat landraces represent important sources to resistance to diseases [7,44,45,46,47] and to abiotic stresses such as drought and salinity [14], while providing useful genes for other traits including quality [8,48].

Taking into consideration the role of the pathogen in host–pathogen interactions, a change in the fungal virulence following sexual recombination [27,49] could lead to new virulence types [22] that overcome resistance controlled by major genes when used solo. In the case of *Z. tritici*, the causal agent of Septoria tritici blotch disease—one of the most devastating diseases in the Mediterranean basin, and particularly in Tunisia [24]—it seems likely that most resistance genes will not last long because of the pathogen’s’s high diversity and genome plasticity [27,28,29,30,50,51,52]. Hence, there will be a continual need to identify new strategies for the deployment of durable resistance. Durum wheat landraces remain a reservoir of genetic diversity, and thus, are a powerful tool for the introgression of novel sources of resistance to STB in commercial breeding programs. However, the exploration of diversity for resistance in a suite of Tunisian landraces, though promising, has just begun [35,36,37]. Thus, screening durum wheat landraces for resistance to *Z. tritici* is crucial, as they contain allelic variation for agronomic and disease-resistance traits such as resistance to *Z. tritici* [3,11,16].

### 4.1. Mining for Novel Resistance to Septoria Tritici Blotch in the Med-Collection

In our study, we aimed to discriminate the various resistance levels of the full set of the USDA collection in 2016–2017. Testing 3166 USDA durum wheat accessions under field conditions gave us useful information pertaining to the potential exploitation of Mediterranean durum wheat landraces originating from similar agroecological zones, in the search for disease-resistance sources. During the following cropping season of 2018–2019, we opted to reduce the population size to 1059 accessions by excluding accessions that were not adapted during the 2016 season, were highly susceptible to yellow rust, were winter types, and that failed to germinate. From this collection, we further scrutinized durum accessions mainly from six Mediterranean countries using a 0–5 scale for the seedling assessment at GS11–20 [38], and the double-digit scale 00–99 [20] at the adult growth stage (GS37–87). These accessions originated mostly from Algeria, France, Italy, Portugal, Spain and Tunisia. Screening under field conditions in Tunisia is of great relevance to agroecology in the Mediterranean area; according to Bari et al. [53] and Street et al. [54], “novel genetic variation for resistance to pests and diseases can be detected in plant genetic resources originating from locations with an environmental profile similar to the collection sites of a reference set of accessions with known resistance, based on the Focused Identification of Germplasm Strategy (FIGS) approach”. Hence the field data collected over two seasons could lead to the development of FIGS-Septoria. Moreover, in search of sources of resistance, we would seek accessions from specific agroecological zones [6,7,55]. Thus, phenotyping the populations under Tunisian agroecological conditions (hot spot for STB) would result in discrimination within and between populations for eventual exploitation by collaborators in the Mediterranean region.

### 4.2. Comparison of Seedling and Adult-Plant Resistance

Kodia experimental station facilities (land, possibility of irrigation, planting under zero tillage, climatic conditions favorable for disease development) allowed us to assess seedling resistance/susceptibly under field conditions for the first time, and to discern correlations between seedling and adult-plant resistance under the same conditions for two seasons. In our experiments, considering the existence of a significant correlation between seedling and adult-plant resistance, evaluated under field conditions, the reaction at the seedling stage will most likely remain the same at the adult stage for most genotypes. This finding may be helpful as it will also allow screening genotypes at the seedling stage under field conditions against multiple strains/virulence types.

In many plant–pathogen interactions, plant disease resistance/susceptibility depends on many factors, including environmental conditions, the nature of the infected tissue, the genotypic combination of the host species and the pathogen, as well as the developmental stage at which the plant is infected [56]. In early growth stages, plants tend to be more susceptible to disease compared to late growth stages; this may indicate an increase in resistance over time, with plants already resistant to a pathogen increasing their ability to control infection and colonization at a precise growth phase. On the other hand, a host plant susceptible to a virulent pathogen at early stages of growth may acquire disease resistance during its development [57]. It is well known that, following infection by a microbial pathogen, susceptible plants can develop an enhanced resistance to further infection, known as a systemic resistance response [56,58,59]. This reaction can also be associated with an ability to ‘recall’ previous infection, known as priming, resulting in plants responding more rapidly and effectively the second time they encounter pathogen attack [60].

Although infection at the seedling stage could have influenced the analysis of phenotypic data at the adult stage, we were mostly interested in accessions that remained resistant at both growth stages across the two years, as they may harbor all of the stage-resistance genes/QTLs that are not likely to break down quickly. For example, the two qualitative genes that were identified in bread wheat, *Stb4* and *Stb5*, were found to confer resistance at both physiological stages [61,62].

To ensure adequate levels of Septoria infection, we used straw and artificial inoculations with diverse Septoria populations collected from the same region, coupled with naturally occurring *Z. tritici* that varied between years, possibly due to the occurrence of the sexual cycle; this resulted in the variability of *Z. tritici* virulence factors [27,31], which was also indicated by the reactions of national checks and possibly the variability within and between populations [6,9]. In this study, we observed different reactions to Septoria between the genotypes and the ANOVA analysis revealed that the genotype term is highly significant at both the seedling and adult stages, emphasizing that the materials had diverse genetic backgrounds. Several phenotypic classes were subsequently recognized and effective sources of resistance to *Z. tritici* were identified.

### 4.3. Diverse Sources of Resistance to STB and the Relationship among Disease-Resistance Traits and Plant Height

The same observation was established for PH, which had a significant effect on adult-stage resistance. The negative association between plant height and resistance is in accordance with previous reports [63,64,65]. While several scientists have reported genetic associations between increased disease severity and shortness [64,66,67], more recent studies by Arama et al. [68], Simon et al. [69] and Arraiano et al. [70], did not detect any genetic associations between STB resistance and plant height, and concluded that the negative association was most probably due to environmental or epidemiological factors. Hence, it is necessary to consider that other factors rather than genetic linkages among these traits could explain this association. Likewise, previous mapping studies also reported that STB was negatively correlated with earliness. Hence, tall and late wheat genotypes in general were found to be less prone to STB infection than short and early-heading ones [63,71,72,73]. Nevertheless, Arama et al. [68] reported no influence of heading date when cultivars were evaluated at the same developmental stage and under similar weather conditions. Therefore, in order to reduce the effect of heading date on disease resistance/susceptibility, disease severity should be measured at the same stage of development. In a more recent study, Goudermand et al. [73] identified QTLs/MQTLs of resistance that overlapped with QTLs of earliness but were not considered as foliar resistance per se as these were due to differences in leaf age and, as well as to differences in the duration of the period that leaves were exposed to the disease.

In our study, heading dates were not recorded in our field trials, but should be the subject of further investigation to shed further light on the effect of this trait on STB infection. While phenology could influence the evaluation of the reaction to STB by allowing disease escape by tall and late genotypes, in our experiments, disease evaluation was carried out during the same period when the flag leaf was fully emerged for all the accessions, and at several consecutive times. Hence, even though the possibility of environmental effect exists, it is not major, as not only did we ensure repeated readings, but all of the accessions were also continuously exposed to the susceptible infected spreader; moreover, continuous inoculations, coupled with irrigation, ensured optimal infections. Indeed, the checks implemented within the experimental design exhibited a uniform infection. Moreover, ascospores, released during all plant growth stages that have been reported in Tunisia [31], could reduce the effect of plant height and heading date in the expression of the disease, and therefore minimize the epidemiological aspect.

Moreover, the PH term was highly significant at the adult stage, meaning it affects STB infection, further indicating that seedling tests under controlled conditions are not fully informative/effective for STB resistance screening; moreover, it indicates that this needs to be further confirmed in the field at the adult stage. In fact, some qualitative genes identified at the seedling stage, such as *Stb7* and *Stb10*, were not identified/effective at the adult stage [71,74]. The PCA clearly showed a substantial set of durum wheat landraces with stable resistance over the seedling and the adult plant stages. Resistance level type HR at the seedling and adult growth stages could carry novel single major-resistance genes, while reaction R might have more than one resistance gene, as the disease progress—assessed using the rAUDPC of R reactions—progressively changed (GS37–87), but did not reach the MR type. MR reactions resulting from shifts from HR, R, MR in 2017 to MR in 2019 could carry combinations of major resistance or even minor genes [18,35,36], which could be confirmed further in a breeding program based on crosses made from these accessions. Genome-wide association mapping of these accessions could possibly reveal novel adult-plant resistance (APR) genes/QTLS that are effective either at the seedling or adult stages, or at both stages. This is essential, particularly in durum wheat, knowing that the current known resistance genes to STB have been identified in bread wheat (*Triticum aestivum*). Adequate levels of resistance at the seedling stage would be of great use in areas where *Z. tritici* does not go through sexual recombination in its life cycle, whereas in Tunisia, this type of resistance will not be of use; this is because Septoria could have more than one sexual cycle during the crop season [31]. Hence, a combination of resistance levels at the seedling and adult growth stages may provide effective and durable resistance to STB. HR at the adult stage could prevent incoming infection by exogenous airborne ascospores. It is essential to have clear knowledge of *Z. tritici* evolution/genetic diversity [22], as the shifts int he pathogen’s virulence could lead to a breakdown of resistance, particularly the dominant resistance governed by major genes. Nevertheless, a breakdown in resistance could happen over all levels of resistance types, but is less likely with the pyramiding of minor and major genes, which is considered the best strategy in breeding against fungal diseases with high genetic diversity and frequency recombination [36,75,76,77,78,79,80,81].

### 4.4. The Effect of Population Origin and Common Name on STB Resistance

When comparing reaction types among and between countries and between the different growth stages, for some of the countries, the levels of reaction were maintained between the seedling and adult stages. Conversely, for others, the levels of resistance were variable. For instance, Portugal and Spain showed the highest levels of resistance at both stages, unlike accessions from France and Algeria. Overall, in our study, Portuguese populations had the highest level and the lowest variability of resistance, whereas Tunisian and Italian populations had relatively high variation between and within populations.

Often, landrace populations can vary by country of origin [6,8,9,68], as well as between countries [82] for accessions with the same common name but a different USDA reference (PI/Cltr number) or local reference. Such populations are frequent in the Mediterranean region. For instance, the populations ‘Oued Zenati‘ originating from Algeria and Spain, or ‘Raspinegro’ from Portugal and Spain, had similar levels of resistance, suggesting that these populations may have the same origin or that seed exchange occurred between these two countries. The landraces ‘Bidi’ (Algeria, France, Italy, and Tunisia) and ‘Agini’ (Algeria, Spain, Tunisia) populations showed different levels of resistance/susceptibility between countries, suggesting that although these accessions have the same name, there might be different genotypes. Further genotyping of these accessions would reveal their origin and similarity/diversity.

Hence, this study lays the foundation to further explore this Med-collection, to identify the APR genes/QTLs involved in STB resistance, mainly through GWAS studies or mapping populations.

## 5. Conclusions

The results of this study showed that durum wheat accessions deposited at the USDA National Small Grains Collection provided a good and diverse source of resistance to Septoria tritici blotch disease. The Med-collection of 1059 accessions, mostly composed of landraces, mainly originated from Algeria, France, Italy, Portugal, Spain and Tunisia. The genotype term was found to be highly significant at both the seedling and adult stages, emphasizing the high genetic variability of the tested accessions. PH was found to have a significant negative effect on adult-stage resistance, indicating that the association between this trait and disease severity could be either phenotypic or genetic. When comparing reaction types among and between countries, high diversity was observed with regard to STB resistance and PH. Some accessions with the same name or different origins were found to have different reactions to STB, suggesting that these accessions may in fact be different genetically. Many accessions (92%) showed a similar resistance level at both seedling and adult stages over the two testing seasons, indicating that these accessions may harbor potential novel QTLs/genes. Genotyping of these resistant germplasms will further reveal their genetic similarity/dissimilarity, but also facilitate the characterization and mapping of effective Septoria resistance genes that could be exploited for the improvement of STB resistance in durum wheat.

## Figures and Tables

**Table 1:** Reaction types of the Med-collection at the seedling stage against *Z. tritici*.

Reaction Type at GS (11–20)	Scale 0–5	Symptom Descriptions
Seedling HR	0–1	Lesion not apparent or very small
Seedling R	2	Apparent small lesion
Seedling MR	3	Typical *Z. tritici* lesions (pycnidia) on leaves 2–3
Seedling MS	4	Well-developed lesion up to third and fourth leaves
Seedling S	5	Clear, susceptible reaction present on all leaves

HR: highly resistant; R: resistant; MR: moderately resistant; MS: moderately susceptible; S: susceptible.

**Table 2:** Reaction types of the Med-collection at the adult stage against *Z. tritici*.

Reaction Types at GS (37–87)	rAUDPC Range	Symptom Descriptions
Adult HR	<0.2	No symptoms or small lesions at lower leaves
Adult R	0.2–0.4	Apparent infection at lower leaves but small lesions
Adult MR	0.4–0.6	Mid-height infection, and apparent lesions not well developed
Adult MS	0.6–0.8	Well defined lesions up to flag leaf, and well-developed lesions up to F^−1^ *
Adult S	>0.8	Well-developed lesions up to flag leaf

HR: highly resistant; R: resistant; MR: moderately resistant; MS: moderately susceptible; S: susceptible. * F^−1^ corresponds to the leaf below the flag leaf.

**Table 3:** Disease reaction at adult growth stage of the 3166 Mediterranean landraces used in this study.

2016	HR	R	MR	MS	S	Missing	TOTAL
Subset	1853	800	268	129	97	19	3166
Algeria	58	119	28	3	6	0	214
France	28	24	10	10	7	0	79
Italy	122	62	41	30	20	0	275
Portugal	317	59	10	1	1	1	389
Spain	104	18	6	0	0	0	128
Tunisia	155	111	21	7	5	0	299
Other	1069	407	152	78	58	18	1782
Total	1853	800	268	129	97	19	3166

HR: highly resistant; R: resistant; MR: moderately resistant; MS: moderately susceptible; S: susceptible.

**Table 4:** Analysis of variance (ANOVA) of the relative area under the disease progress curve on the Med-collection inoculated by *Z. tritici*, at seedling and adult plant stages, during two cropping seasons (2016–2017 and 2018–2019).

Physiological Stage	Source of Variation	Sum of Squares	Mean of Squares	F Value	Pr (>F)
Seedling	Genotype	3344	3.160	2.518	<2 × 10^−16^ ***
	Residuals	1218	1.255		
	Year	1	0.635	0.282	0.595
	Residuals	4561	2.249		
	Genotype × Year	1236.302	0.317	0.9238	0.9238
	Residuals	4.000	4.00		
Adult	Year	0.34	0.335	6.046	0.014 *
	Residuals	117.17	0.055		
	Genotype	88.15	0.083	2.988	<2 × 10^−16^ ***
	Residuals	29.36	0.027		
	Genotype × Year	29.164	0.027	0.524	0.832537
	Residuals	0.529	0.529		
	Seedling	36.1	36.17	958.6	<2 × 10^−16^ ***
	Residuals	76.49	0.04		
	PH	23.45	0.868	19.48	<2 × 10^−16^ ***
	Residuals	89.22	0.044		
	Level of improvement	14.96	3.739	77.46	<2 × 10^−16^ ***
	Residuals	97.71	0.048		

Significance codes: *** highly significant as *p* ≤ 0.001; * significant as *p* ≤ 0.05.

**Table 5:** Pearson’s correlation coefficient for the disease parameters and agronomic traits among the wheat genotypes across seasons (2016–2017 and 2018–2019).

	Seedling 2019	PH2017	rAUDPC2019
Seedling 2017	*r* = 0.431 *p* < 2.2 × 10^−16^ ***	*-*	*-*
PH 2019	*-*	*r* = 0.679 *p* *<* 2.2 × 10^−16^ ***	*r* = −0.407 *p* < 2.2 × 10^−16^ ***
rAUDPC 2017	*-*	*r* = −0.264 *p* < 2.2 × 10^−16^ ***	*r* = 0.512 *p* < 2.2 × 10^−16^ ***

AUDPC = area under the disease progress curve; rAUDPC = relative area under the disease progress curve; PH = plant height; *** highly significant as *p* ≤ 0.001.

## Data Availability

The data presented in this study are available within the article.

## Supplementary Materials

The following are available online at: https://www.mdpi.com/article/10.3390/genes13020355/s1, Table S1: data on disease evaluation based on seedling and adult scores (AUDPC and rAUDPC) under field conditions, as well as agronomic data such as plant height of the Mediterranean collection (1059 accessions) over two seasons, Table S2: Ranking of mean area under disease progress curve (AUDPC) and plant height (PH) of checks (Karim, Salim and Nasr) evaluated in the field during two seasons (2016–2017 and 2018–2019) against *Z. tritici*.
