# Supplementary material for: Phenotyping Mediterranean Durum Wheat Landraces for Resistance to Zymoseptoria tritici in Tunisia
Source: Genes (Basel). 2022 Feb 16;13(2):355. doi: 10.3390/genes13020355 (PMC8872163; doi:10.3390/genes13020355)
Supplement: Supplementary file 1 [file genes-13-00355-s001.zip › genes-1540703-supplementary.pdf]

Table S1. Data on disease evaluation based on seedling and adult scores (AUDPC & rAUDPC) under field conditions as well as agronomic data such as plant height of the Mediterranean collection (1059 accessions) over two seasons.

Accession	Taxonomy	Origin	Level Of Improvement	PH 2017	PH2019	STBSD2017	class (SD2017)	AUDPC 2017	rAUDPC 2017	class (Adult 2017)	STB SD2019	class (SD2019)	AUDPC 2019	rAUDPC 2019	class (Adult 2019)	cluster 5A	cluster 5B
PI 57066	31	Algeria	Landrace	110	125	2	R	350	0,23	R	3	MR	308,64	0,39	R	2	4
PI 57067	34	Algeria	Landrace	120	150	2	R	246,3	0,16	HR	4	MS	281,48	0,36	R	2	1
PI 57068	41	Algeria	Landrace	120	135	4	MS	756,17	0,49	MR	5	S	709,88	0,9	S	3	-
PI 57069	45	Algeria	Landrace	120	135	2	R	350	0,23	R	2	R	350,62	0,45	MR	2	-
PI 57070	49	Algeria	Landrace	130	140	2	R	246,3	0,16	HR	2	R	281,48	0,36	R	2	1
PI 576807	1015	Algeria	Landrace	120	115	3	MR	777,78	0,51	MR	NA	NA	143,21	0,18	HR	2	-
CItr 1594	Adjini	Algeria	Landrace	130	140	4	MS	604,94	0,39	R	5	S	474,07	0,6	MS	2	-
CItr 3844	Agini	Algeria	Landrace	150	140	2	R	388,89	0,25	R	0	HR	565,43	0,72	MS	2	-
PI 520099	Algerian Line 27	Algeria	Breeding material	75	70	5	S	1378,4	0,9	S	4	MS	1234,57	1,57	S	3	2
PI 520100	Algerian Line 33	Algeria	Breeding material	95	85	3	MR	1158,02	0,75	MS	3	MR	344,44	0,44	MR	3	-
PI 520074	Algerian Line 38	Algeria	Breeding material	90	85	4	MS	1274,69	0,83	S	3	MR	795,06	1,01	S	3	2
PI 520101	Algerian Line 46	Algeria	Breeding material	100	100	5	S	1274,69	0,83	S	3	MR	814,81	1,04	S	3	2
CItr 3842	Beliouni	Algeria	Landrace	130	150	4	MS	738,89	0,48	MR	0	HR	474,07	0,6	MS	2	-
CItr 3848	Beliouni	Algeria	Landrace	140	195	3	MR	535,8	0,35	R	0	HR	344,44	0,44	MR	2	-
CItr 3852	Beliouni	Algeria	Landrace	120	130	5	S	851,23	0,55	MR	5	S	866,67	1,1	S	3	-
PI 576735	Bidi 17	Algeria	Landrace	80	150	4	MS	423,46	0,28	R	3	MR	190,12	0,24	R	2	4
PI 576736	Bidi 17	Algeria	Landrace	115	145	0	HR	661,11	0,43	MR	3	MR	88,89	0,11	HR	2	-
PI 576791	Bidi 17	Algeria	Landrace	140	145	4	MS	531,48	0,35	R	3	MR	116,05	0,15	HR	2	1
CItr 3847	Ble de Seville	Algeria	Unknown improvement status	150	145	2	R	393,21	0,26	R	1	HR	459,26	0,58	MR	2	-
CItr 1471	Ble Dur	Algeria	Landrace	135	110	3	MR	466,67	0,3	R	0	HR	288,89	0,37	R	2	1
CItr 1483	Ble Dur	Algeria	Landrace	125	110	3	MR	345,68	0,22	R	0	HR	285,19	0,36	R	2	4

PI 534437	Hedba	Algeria	Landrace	160	150	4	MS	712,96	0,46	MR	5	S	277,78	0,35	R	2	-
PI 576738	Hedba	Algeria	Landrace	120	145	3	MR	241,98	0,16	HR	3	MR	274,07	0,35	R	2	1
PI 576741	Hedba	Algeria	Landrace	160	150	4	MS	337,04	0,22	R	3	MR	340,74	0,43	MR	2	-
PI 576743	Hedba	Algeria	Landrace	165	140	4	MS	557,41	0,36	R	4	MS	251,85	0,32	R	2	1
PI 576751	Hedba	Algeria	Landrace	140	155	3	MR	328,4	0,21	R	4	MS	303,7	0,39	R	2	1
PI 576780	Hedba	Algeria	Landrace	125	145	3	MR	505,56	0,33	R	NA	NA	159,26	0,2	R	2	1
PI 263418	Hedba 3	Algeria	Cultivar	115	125	4	MS	604,94	0,39	R	4	MS	637,04	0,81	S	3	2
PI 534389	Hedba 3	Algeria	Cultivar	130	130	0	HR	635,19	0,41	MR	2	R	343,21	0,44	MR	2	-
CItr 3856	ICM 111	Algeria	Landrace	130	155	3	MR	401,85	0,26	R	4	MS	459,26	0,58	MR	2	-
PI 527460	Kammah	Algeria	Landrace	125	135	2	R	246,3	0,16	HR	2	R	274,07	0,35	R	2	1
PI 527463	Kammeh	Algeria	Landrace	140	115	4	MS	466,67	0,3	R	NA	NA	218,52	0,28	R	2	1
PI 352444	Langlois 1527	Algeria	Cultivar	140	125	3	MR	466,67	0,3	R	3	MR	343,21	0,44	MR	2	-
PI 7792	Mahmoudi	Algeria	Landrace	120	150	3	MR	449,38	0,29	R	2	R	376,54	0,48	MR	2	-
PI 11715	Marouani	Algeria	Landrace	130	145	0	HR	0	0	HR	5	S	707,41	0,9	S	2	1
PI 470820	MG 17964	Algeria	Landrace	160	150	3	MR	626,54	0,41	MR	2	R	237,04	0,3	R	2	-
PI 470821	MG 17965	Algeria	Landrace	150	150	3	MR	622,22	0,4	MR	2	R	212,35	0,27	R	2	-
PI 470822	MG 17966	Algeria	Landrace	130	130	1	HR	350	0,23	R	3	MR	192,59	0,25	R	2	1
PI 470827	MG 17972	Algeria	Landrace	100	155	4	MS	466,67	0,3	R	3	MR	266,67	0,34	R	2	1
PI 470830	MG 17975	Algeria	Landrace	130	145	3	MR	466,67	0,3	R	4	MS	288,89	0,37	R	2	1
PI 470831	MG 17976	Algeria	Landrace	150	165	3	MR	466,67	0,3	R	2	R	251,85	0,32	R	2	1
PI 470832	MG 17977	Algeria	Landrace	150	155	4	MS	466,67	0,3	R	3	MR	356,79	0,45	MR	2	-
PI 470835	MG 17981	Algeria	Landrace	150	140	3	MR	496,91	0,32	R	2	R	558,02	0,71	MS	2	-
PI 470836	MG 17983	Algeria	Landrace	160	155	3	MR	410,49	0,27	R	3	MR	419,75	0,53	MR	2	-
PI 470837	MG 17984	Algeria	Landrace	140	140	1	HR	432,1	0,28	R	3	MR	274,07	0,35	R	2	1
PI 470840	MG 17987	Algeria	Landrace	165	170	4	MS	535,8	0,35	R	4	MS	303,7	0,39	R	2	1
PI 470842	MG 17990	Algeria	Landrace	160	150	4	MS	691,36	0,45	MR	3	MR	343,21	0,44	MR	2	-
PI 470844	MG 17992	Algeria	Landrace	140	145	0	HR	337,04	0,22	R	2	R	474,07	0,6	MS	2	-
PI 470845	MG 17993	Algeria	Landrace	150	145	3	MR	509,88	0,33	R	3	MR	233,33	0,3	R	2	1
PI 470846	MG 17994	Algeria	Landrace	145	150	3	MR	509,88	0,33	R	2	R	195,06	0,25	R	2	1

PI 470847	MG 17995	Algeria	Landrace	140	165	4	MS	561,73	0,37	R	3	MR	274,07	0,35	R	2	1
PI 470848	MG 17996	Algeria	Landrace	140	155	3	MR	712,96	0,46	MR	3	MR	251,85	0,32	R	2	-
PI 470850	MG 17998	Algeria	Landrace	115	165	3	MR	583,33	0,38	R	2	R	376,54	0,48	MR	2	-
PI 470854	MG 18003	Algeria	Landrace	135	120	0	HR	453,7	0,3	R	2	R	237,04	0,3	R	2	1
PI 470858	MG 18007	Algeria	Landrace	130	120	2	R	350	0,23	R	2	R	288,89	0,37	R	2	1
PI 470859	MG 18008	Algeria	Landrace	140	135	2	R	388,89	0,25	R	2	R	143,21	0,18	HR	2	1
PI 470870	MG 18019	Algeria	Landrace	150	155	4	MS	604,94	0,39	R	2	R	196,3	0,25	R	2	1
PI 470872	MG 18021	Algeria	Landrace	80	95	4	MS	1062,96	0,69	MS	NA	NA	1046,91	1,33	S	3	-
PI 470874	MG 18025	Algeria	Landrace	150	150	3	MR	449,38	0,29	R	NA	NA	128,4	0,16	HR	2	1
PI 470877	MG 18028	Algeria	Landrace	140	135	3	MR	350	0,23	R	NA	NA	143,21	0,18	HR	2	1
PI 470878	MG 18029	Algeria	Landrace	135	155	0	HR	414,81	0,27	R	3	MR	229,63	0,29	R	2	1
PI 470880	MG 18032	Algeria	Landrace	145	155	3	MR	328,4	0,21	R	3	MR	192,59	0,25	R	2	1
PI 470881	MG 18033	Algeria	Landrace	140	155	3	MR	350	0,23	R	2	R	128,4	0,16	HR	2	1
PI 470882	MG 18034	Algeria	Landrace	140	150	3	MR	557,41	0,36	R	2	R	159,26	0,2	R	2	1
PI 470883	MG 18035	Algeria	Landrace	165	145	4	MS	591,98	0,39	R	2	R	303,7	0,39	R	2	1
PI 470885	MG 18038	Algeria	Landrace	150	160	3	MR	591,98	0,39	R	3	MR	128,4	0,16	HR	2	1
PI 470886	MG 18039	Algeria	Landrace	165	155	4	MS	695,68	0,45	MR	2	R	116,05	0,15	HR	2	-
PI 470887	MG 18041	Algeria	Landrace	165	145	4	MS	505,56	0,33	R	3	MR	193,83	0,25	R	2	1
PI 470888	MG 18042	Algeria	Landrace	170	155	4	MS	475,31	0,31	R	3	MR	128,4	0,16	HR	2	1
PI 470889	MG 18043	Algeria	Landrace	165	165	4	MS	717,28	0,47	MR	3	MR	128,4	0,16	HR	2	-
PI 470890	MG 18044	Algeria	Landrace	165	150	4	MS	527,16	0,34	R	2	R	196,3	0,25	R	2	1
PI 470891	MG 18045	Algeria	Landrace	155	140	3	MR	423,46	0,28	R	2	R	212,35	0,27	R	2	1
PI 470892	MG 18046	Algeria	Landrace	160	150	4	MS	505,56	0,33	R	4	MS	212,35	0,27	R	2	1
PI 470894	MG 18048	Algeria	Landrace	165	155	3	MR	557,41	0,36	R	2	R	251,85	0,32	R	2	1
PI 470896	MG 18050	Algeria	Landrace	160	145	3	MR	501,23	0,33	R	3	MR	288,89	0,37	R	2	1
PI 470897	MG 18051	Algeria	Landrace	165	150	4	MS	241,98	0,16	HR	3	MR	328,4	0,42	MR	2	-
PI 470898	MG 18052	Algeria	Landrace	170	155	3	MR	371,6	0,24	R	2	R	237,04	0,3	R	2	1
PI 470899	MG 18053	Algeria	Landrace	165	160	4	MS	591,98	0,39	R	4	MS	162,96	0,21	R	2	1
PI 470900	MG 18054	Algeria	Landrace	170	160	5	S	717,28	0,47	MR	3	MR	188,89	0,24	R	2	-

PI 470901	MG 18055	Algeria	Landrace	155	150	4	MS	466,67	0,3	R	2	R	128,4	0,16	HR	2	1
PI 470902	MG 18057	Algeria	Landrace	160	150	4	MS	397,53	0,26	R	2	R	170,37	0,22	R	2	1
PI 470907	MG 18062	Algeria	Landrace	155	145	4	MS	561,73	0,37	R	2	R	177,78	0,23	R	2	1
PI 470908	MG 18065	Algeria	Landrace	155	145	4	MS	380,25	0,25	R	3	MR	172,84	0,22	R	2	1
PI 470909	MG 18066	Algeria	Landrace	150	150	4	MS	639,51	0,42	MR	4	MS	128,4	0,16	HR	2	-
PI 470910	MG 18067	Algeria	Landrace	125	140	1	HR	401,85	0,26	R	2	R	237,04	0,3	R	2	-
PI 470911	MG 18068	Algeria	Landrace	140	145	3	MR	738,89	0,48	MR	2	R	128,4	0,16	HR	2	-
PI 470913	MG 18070	Algeria	Landrace	145	135	3	MR	466,67	0,3	R	2	R	148,15	0,19	HR	2	1
PI 576729	MG 18169	Algeria	Landrace	150	145	4	MS	661,11	0,43	MR	2	R	237,04	0,3	R	2	-
PI 576734	MG 18174	Algeria	Landrace	145	150	3	MR	246,3	0,16	HR	3	MR	237,04	0,3	R	2	1
PI 576737	MG 18180	Algeria	Landrace	130	90	3	MR	786,42	0,51	MR	3	MR	948,15	1,21	S	3	-
PI 534394	MG 18183	Algeria	Landrace	150	155	4	MS	518,52	0,34	R	4	MS	122,22	0,16	HR	2	1
PI 576739	MG 18186	Algeria	Landrace	120	130	3	MR	453,7	0,3	R	3	MR	251,85	0,32	R	2	1
PI 576740	MG 18187	Algeria	Landrace	160	145	4	MS	557,41	0,36	R	2	R	401,23	0,51	MR	2	-
PI 576742	MG 18189	Algeria	Landrace	150	125	4	MS	557,41	0,36	R	4	MS	322,22	0,41	MR	2	-
PI 576744	MG 18191	Algeria	Landrace	140	120	3	MR	583,33	0,38	R	4	MS	229,63	0,29	R	2	1
PI 576745	MG 18192	Algeria	Landrace	165	155	4	MS	712,96	0,46	MR	3	MR	218,52	0,28	R	2	-
PI 576746	MG 18193	Algeria	Landrace	160	170	4	MS	540,12	0,35	R	3	MR	303,7	0,39	R	2	1
PI 576747	MG 18194	Algeria	Landrace	140	155	3	MR	371,6	0,24	R	4	MS	128,4	0,16	HR	2	1
PI 576749	MG 18197	Algeria	Landrace	125	135	1	HR	449,38	0,29	R	4	MS	88,89	0,11	HR	2	1
PI 534409	MG 18198	Algeria	Landrace	150	130	4	MS	483,95	0,31	R	2	R	119,75	0,15	HR	2	1
PI 470871	MG 18020	Algeria	Landrace	95	105	0	HR	207,41	0,13	HR	NA	NA	214,81	0,27	R	1	4
PI 576750	MG 18200	Algeria	Landrace	140	130	2	R	483,95	0,31	R	3	MR	175,31	0,22	R	2	1
PI 576752	MG 18202	Algeria	Landrace	150	165	4	MS	738,89	0,48	MR	4	MS	128,4	0,16	HR	2	-
PI 576753	MG 18203	Algeria	Landrace	155	140	4	MS	609,26	0,4	R	4	MS	298,77	0,38	R	2	1
PI 576754	MG 18204	Algeria	Landrace	170	145	4	MS	674,07	0,44	MR	5	S	274,07	0,35	R	2	-
PI 576755	MG 18205	Algeria	Landrace	160	145	4	MS	406,17	0,26	R	4	MS	244,44	0,31	R	2	1
PI 576756	MG 18206	Algeria	Landrace	150	140	4	MS	669,75	0,44	MR	3	MR	281,48	0,36	R	2	-
PI 576757	MG 18207	Algeria	Landrace	140	155	3	MR	311,11	0,2	R	4	MS	251,85	0,32	R	2	1

PI 576758	MG 18208	Algeria	Landrace	145	160	4	MS	337,04	0,22	R	5	S	367,9	0,47	MR	2	-
PI 576759	MG 18209	Algeria	Landrace	150	140	4	MS	505,56	0,33	R	4	MS	251,85	0,32	R	2	1
PI 576760	MG 18210	Algeria	Landrace	150	135	4	MS	557,41	0,36	R	3	MR	340,74	0,43	MR	2	-
PI 534422	MG 18211	Algeria	Landrace	140	130	5	S	583,33	0,38	R	3	MR	138,27	0,18	HR	2	1
PI 576762	MG 18213	Algeria	Landrace	90	155	5	S	1508,02	0,98	S	2	R	258,02	0,33	R	3	2
PI 534426	MG 18215	Algeria	Landrace	150	150	3	MR	505,56	0,33	R	2	R	119,75	0,15	HR	2	1
PI 576764	MG 18215	Algeria	Landrace	165	150	4	MS	609,26	0,4	R	2	R	266,67	0,34	R	2	1
PI 534427	MG 18216	Algeria	Landrace	150	145	4	MS	505,56	0,33	R	3	MR	128,4	0,16	HR	2	1
PI 534428	MG 18217	Algeria	Landrace	140	135	4	MS	410,49	0,27	R	1	HR	125,93	0,16	HR	2	1
PI 534429	MG 18218	Algeria	Landrace	130	140	3	MR	557,41	0,36	R	NA	NA	128,4	0,16	HR	2	1
PI 576765	MG 18220	Algeria	Landrace	170	155	4	MS	635,19	0,41	MR	2	R	218,52	0,28	R	2	-
PI 534432	MG 18221	Algeria	Landrace	150	145	3	MR	583,33	0,38	R	3	MR	162,96	0,21	R	2	1
PI 576766	MG 18222	Algeria	Landrace	155	155	4	MS	591,98	0,39	R	2	R	288,89	0,37	R	2	1
PI 576767	MG 18223	Algeria	Landrace	130	150	3	MR	613,58	0,4	R	4	MS	218,52	0,28	R	2	1
PI 534435	MG 18224	Algeria	Landrace	150	145	4	MS	756,17	0,49	MR	4	MS	251,85	0,32	R	2	-
PI 576768	MG 18225	Algeria	Landrace	130	155	4	MS	466,67	0,3	R	NA	NA	177,78	0,23	R	2	1
PI 534438	MG 18227	Algeria	Landrace	160	155	4	MS	967,9	0,63	MS	5	S	125,93	0,16	HR	2	-
PI 576769	MG 18228	Algeria	Landrace	150	140	4	MS	505,56	0,33	R	NA	NA	177,78	0,23	R	2	1
PI 576770	MG 18230	Algeria	Landrace	150	140	3	MR	337,04	0,22	R	NA	NA	140,74	0,18	HR	2	1
PI 534442	MG 18231	Algeria	Landrace	150	155	4	MS	505,56	0,33	R	4	MS	128,4	0,16	HR	2	1
PI 576772	MG 18235	Algeria	Landrace	130	135	4	MS	380,25	0,25	R	NA	NA	214,81	0,27	R	2	1
PI 576773	MG 18236	Algeria	Landrace	150	145	4	MS	557,41	0,36	R	4	MS	159,26	0,2	R	2	1
PI 576777	MG 18240	Algeria	Landrace	140	140	1	HR	466,67	0,3	R	3	MR	177,78	0,23	R	2	1
PI 576778	MG 18242	Algeria	Landrace	135	150	3	MR	350	0,23	R	2	R	200	0,25	R	2	1
PI 576779	MG 18243	Algeria	Landrace	140	155	3	MR	591,98	0,39	R	2	R	128,4	0,16	HR	2	1
PI 576781	MG 18246	Algeria	Landrace	160	135	4	MS	337,04	0,22	R	NA	NA	190,12	0,24	R	2	1
PI 576782	MG 18248	Algeria	Landrace	160	155	4	MS	712,96	0,46	MR	NA	NA	288,89	0,37	R	2	-
PI 576784	MG 18250	Algeria	Landrace	140	125	4	MS	548,77	0,36	R	NA	NA	140,74	0,18	HR	2	1
PI 534465	MG 18254	Algeria	Landrace	160	165	4	MS	423,46	0,28	R	4	MS	218,52	0,28	R	2	1

PI 470861	MG 18010	Algeria	Landrace	100	115	0	HR	69,14	0,04	HR	2	R	214,81	0,27	R	1	4
PI 576792	MG 18264	Algeria	Landrace	155	165	4	MS	574,69	0,37	R	2	R	237,04	0,3	R	2	1
PI 576793	MG 18265	Algeria	Landrace	150	130	2	R	449,38	0,29	R	3	MR	128,4	0,16	HR	2	1
PI 576796	MG 18268	Algeria	Landrace	140	125	4	MS	466,67	0,3	R	NA	NA	128,4	0,16	HR	2	1
PI 7793	Mohamed Ben Bachir	Algeria	Landrace	120	185	3	MR	345,68	0,22	R	4	MS	716,05	0,91	S	2	1
PI 418199	Nadzenija	Algeria	Unknown improvement status	95	85	3	MR	509,88	0,33	R	3	MR	567,9	0,72	MS	3	-
PI 534393	Oued Zenati	Algeria	Landrace	140	145	3	MR	505,56	0,33	R	2	R	76,54	0,1	HR	2	1
PI 352410	Oued Zenati 368	Algeria	Cultivar	150	155	3	MR	466,67	0,3	R	3	MR	200	0,25	R	2	1
PI 5380	Peliss	Algeria	Cultivar	120	165	3	MR	358,64	0,23	R	4	MS	303,7	0,39	R	2	1
PI 263421	Saba 22738	Algeria	Cultivar	130	160	4	MS	583,33	0,38	R	2	R	190,12	0,24	R	2	1
PI 470867	MG 18016	Algeria	Landrace	110	130	0	HR	69,14	0,04	HR	1	HR	128,4	0,16	HR	1	4
PI 362227	Oued Zenati 368	Algeria	Cultivar	110	110	0	HR	69,14	0,04	HR	2	R	76,54	0,1	HR	1	4
PI 534379	MG 18168	Algeria	Landrace	115	100	0	HR	0	0	HR	NA	NA	214,81	0,27	R	1	4
PI 527462	Kameh	Algeria	Landrace	120	125	0	HR	0	0	HR	NA	NA	233,33	0,3	R	1	4
PI 470862	MG 18011	Algeria	Landrace	120	135	0	HR	0	0	HR	1	HR	128,4	0,16	HR	1	3
PI 470863	MG 18012	Algeria	Landrace	120	135	1	HR	246,3	0,16	HR	2	R	155,56	0,2	HR	1	3
PI 470866	MG 18015	Algeria	Landrace	120	145	0	HR	0	0	HR	2	R	101,23	0,13	HR	1	3
PI 470876	MG 18027	Algeria	Landrace	120	115	2	R	246,3	0,16	HR	NA	NA	140,74	0,18	HR	1	4
PI 576771	MG 18232	Algeria	Landrace	120	115	0	HR	69,14	0,04	HR	NA	NA	128,4	0,16	HR	1	4
CItr 3838	Agini	Algeria	Landrace	125	150	0	HR	69,14	0,04	HR	0	HR	269,14	0,34	R	1	3
CItr 3839	Beliouni	Algeria	Landrace	125	150	1	HR	69,14	0,04	HR	1	HR	401,23	0,51	MR	1	-
PI 576731	MG 18171	Algeria	Landrace	125	135	0	HR	69,14	0,04	HR	2	R	101,23	0,13	HR	1	3
PI 534452	MG 18241	Algeria	Landrace	125	135	0	HR	0	0	HR	2	R	175,31	0,22	R	1	3
PI 263420	Oued Zenati 368	Algeria	Cultivar	125	125	0	HR	0	0	HR	4	MS	229,63	0,29	R	1	3
PI 527461	Kammah	Algeria	Landrace	130	135	0	HR	0	0	HR	NA	NA	251,85	0,32	R	1	3
PI 576732	MG 18172	Algeria	Landrace	130	135	3	MR	241,98	0,16	HR	2	R	190,12	0,24	R	1	1

PI 576774	MG 18237	Algeria	Landrace	130	145	0	HR	350	0,23	R	3	MR	128,4	0,16	HR	2	1
PI 534449	MG 18238	Algeria	Landrace	130	140	2	R	69,14	0,04	HR	2	R	288,89	0,37	R	1	3
PI 576775	MG 18238	Algeria	Landrace	130	150	3	MR	298,15	0,19	HR	3	MR	140,74	0,18	HR	1	1
PI 534458	MG 18247	Algeria	Landrace	130	140	1	HR	69,14	0,04	HR	2	R	192,59	0,25	R	1	3
PI 576788	MG 18255	Algeria	Landrace	130	115	0	HR	0	0	HR	NA	NA	116,05	0,15	HR	1	3
PI 534467	MG 18256	Algeria	Landrace	130	145	0	HR	0	0	HR	3	MR	160,49	0,2	R	1	3
PI 534468	MG 18257	Algeria	Landrace	130	135	2	R	69,14	0,04	HR	3	MR	128,4	0,16	HR	1	3
PI 534470	MG 18259	Algeria	Landrace	130	130	0	HR	0	0	HR	4	MS	128,4	0,16	HR	1	3
PI 576733	Oved Zenati	Algeria	Landrace	130	140	0	HR	69,14	0,04	HR	3	MR	101,23	0,13	HR	1	3
PI 534469	Bidi 17	Algeria	Landrace	135	135	0	HR	69,14	0,04	HR	2	R	159,26	0,2	R	1	3
PI 534410	MG 18199	Algeria	Landrace	135	130	2	R	69,14	0,04	HR	1	HR	148,15	0,19	HR	1	3
PI 470856	MG 18005	Algeria	Landrace	140	140	0	HR	69,14	0,04	HR	1	HR	39,51	0,05	HR	1	3
PI 470865	MG 18014	Algeria	Landrace	140	130	0	HR	0	0	HR	2	R	165,43	0,21	R	1	3
PI 534407	MG 18196	Algeria	Landrace	140	135	1	HR	69,14	0,04	HR	2	R	101,23	0,13	HR	1	3
PI 534445	MG 18234	Algeria	Landrace	140	140	0	HR	0	0	HR	2	R	237,04	0,3	R	1	3
PI 576776	MG 18239	Algeria	Landrace	140	155	3	MR	280,86	0,18	HR	2	R	177,78	0,23	R	2	1
PI 576786	MG 18252	Algeria	Landrace	140	120	1	HR	337,04	0,22	R	3	MR	140,74	0,18	HR	2	1
PI 576787	MG 18253	Algeria	Landrace	140	135	3	MR	337,04	0,22	R	NA	NA	123,46	0,16	HR	2	1
PI 576789	MG 18261	Algeria	Landrace	140	120	3	MR	337,04	0,22	R	NA	NA	128,4	0,16	HR	1	1
PI 576790	MG 18262	Algeria	Landrace	140	120	3	MR	267,9	0,17	HR	NA	NA	76,54	0,1	HR	1	3
PI 576794	MG 18266	Algeria	Landrace	140	130	0	HR	155,56	0,1	HR	NA	NA	64,2	0,08	HR	1	3
PI 534480	MG 18269	Algeria	Landrace	140	135	0	HR	0	0	HR	2	R	200	0,25	R	1	3
PI 470860	MG 18009	Algeria	Landrace	145	120	0	HR	69,14	0,04	HR	2	R	101,23	0,13	HR	1	3
PI 576795	MG 18267	Algeria	Landrace	145	130	3	MR	328,4	0,21	R	NA	NA	155,56	0,2	HR	2	1
PI 192861	Hebda	Algeria	Unknown improvement status	150	145	0	HR	0	0	HR	1	HR	107,41	0,14	HR	1	3
PI 470873	MG 18024	Algeria	Landrace	150	150	1	HR	69,14	0,04	HR	NA	NA	130,86	0,17	HR	1	3
PI 576730	MG 18170	Algeria	Landrace	160	140	3	MR	241,98	0,16	HR	3	MR	101,23	0,13	HR	1	3
PI 191780	Egipto	Egypt	Unknown	100	125	0	HR	254,94	0,17	HR	0	HR	103,7	0,13	HR	1	4

			improvement status												
PI 422303	Chap-21563	Egypt	Breeding material	110	125	0	HR	470,99	0,31	R	1	HR	593,83	0,76	MS	3	-
PI 428464	DW92	Egypt	Breeding material	75	75	4	MS	1378,4	0,9	S	2	R	938,27	1,19	S	3	2
PI 191886	Egipto	Egypt	Unknown improvement status	130	110	3	MR	328,4	0,21	R	0	HR	248,15	0,32	R	2	1
PI 191931	Egipto	Egypt	Unknown improvement status	125	135	3	MR	397,53	0,26	R	1	HR	118,52	0,15	HR	2	1
PI 428458	Egypt Local No. 8	Egypt	Unknown improvement status	85	70	4	MS	1192,59	0,78	MS	NA	NA	429,63	0,55	MR	3	-
PI 185608	Beladi 116	Egypt	Cultivar	125	120	2	R	298,15	0,19	HR	1	HR	103,7	0,13	HR	1	4
PI 383417	2581-2-7-13	France	Breeding material	75	95	2	R	267,9	0,17	HR	2	R	39,51	0,05	HR	1	4
PI 352459	T 32	France	Unknown improvement status	75	115	0	HR	69,14	0,04	HR	0	HR	103,7	0,13	HR	1	4
PI 383415	237-4-13-3	France	Breeding material	80	90	0	HR	0	0	HR	0	HR	76,54	0,1	HR	1	4
PI 383420	2530-9-8-16	France	Breeding material	80	90	0	HR	246,3	0,16	HR	1	HR	190,12	0,24	R	1	4
PI 383413	150-7-9-9	France	Breeding material	85	70	0	HR	0	0	HR	0	HR	119,75	0,15	HR	1	4
PI 352457	LD 308	France	Unknown improvement status	85	100	0	HR	155,56	0,1	HR	1	HR	118,52	0,15	HR	1	4
PI 352464	Lez	France	Cultivar	90	120	0	HR	69,14	0,04	HR	2	R	150,62	0,19	HR	1	4
PI 306652	F8-324-1	France	Breeding material	95	100	0	HR	259,26	0,17	HR	NA	NA	76,54	0,1	HR	1	4
PI 306651	F6-503-5	France	Breeding material	100	95	0	HR	462,35	0,3	R	NA	NA	64,2	0,08	HR	1	4
PI 306659	F9-391-3	France	Breeding material	100	92	0	HR	0	0	HR	0	HR	155,56	0,2	HR	1	4
PI 383412	102-6-7-14	France	Breeding material	115	110	3	MR	466,67	0,3	R	2	R	222,22	0,28	R	2	4
PI 383418	2581-6-4-10	France	Breeding material	75	80	3	MR	561,73	0,37	R	1	HR	177,78	0,23	R	3	4

PI 383419	2593-8-5-13	France	Breeding material	85	90	3	MR	669,75	0,44	MR	2	R	392,59	0,5	MR	3	-
PI 306639	Ak Basale 63	France	Breeding material	115	120	3	MR	466,67	0,3	R	3	MR	232,1	0,3	R	2	1
PI 584839	Cargiflash	France	Cultivar	90	85	4	MS	1343,83	0,87	S	NA	NA	440,74	0,56	MR	3	-
PI 584838	Casoar	France	Cultivar	90	75	4	MS	1482,1	0,96	S	NA	NA	429,63	0,55	MR	3	-
PI 548992	Chiddam Blanc de Mars Selection	France	Unknown improvement status	130	140	3	MR	466,67	0,3	R	1	HR	118,52	0,15	HR	2	1
PI 306643	D 117	France	Breeding material	125	125	3	MR	414,81	0,27	R	2	R	148,15	0,19	HR	2	1
PI 306644	D 160	France	Breeding material	105	100	3	MR	466,67	0,3	R	2	R	192,59	0,25	R	2	4
PI 306647	D 211	France	Breeding material	140	115	3	MR	328,4	0,21	R	3	MR	214,81	0,27	R	2	1
PI 306648	D 304	France	Breeding material	155	110	4	MS	311,11	0,2	R	NA	NA	251,85	0,32	R	2	1
PI 428540	Diabolo	France	Cultivar	100	90	4	MS	1136,42	0,74	MS	0	HR	217,28	0,28	R	3	-
PI 371655	Durtal	France	Cultivar	90	75	1	HR	440,74	0,29	R	NA	NA	262,96	0,33	R	3	4
PI 306663	F11-142-1	France	Breeding material	100	95	3	MR	972,22	0,63	MS	NA	NA	159,26	0,2	R	3	-
PI 306645	D 162	France	Breeding material	105	100	0	HR	0	0	HR	2	R	155,56	0,2	HR	1	4
PI 306650	F6 34-8	France	Breeding material	105	80	2	R	350	0,23	R	NA	NA	88,89	0,11	HR	1	4
PI 306655	F8-6172-1	France	Breeding material	105	105	0	HR	0	0	HR	0	HR	64,2	0,08	HR	1	4
CItr 3994	Kavarna	France	Landrace	105	135	0	HR	0	0	HR	0	HR	200	0,25	R	1	4
PI 306660	F9 391-8	France	Breeding material	130	125	3	MR	769,14	0,5	MR	2	R	233,33	0,3	R	2	-
PI 306662	F9 396-8	France	Breeding material	100	105	2	R	786,42	0,51	MR	NA	NA	88,89	0,11	HR	2	-
PI 306661	F9-396-4	France	Breeding material	120	115	2	R	505,56	0,33	R	2	R	175,31	0,22	R	2	1
PI 174662	Iumillo	France	Unknown improvement status	130	135	3	MR	445,06	0,29	R	1	HR	118,52	0,15	HR	2	1
PI 428542	Kidur	France	Cultivar	100	85	5	S	1378,4	0,9	S	3	MR	920,99	1,17	S	3	2
PI 316012	Lez	France	Cultivar	100	90	4	MS	1953,09	1,27	S	4	MS	159,26	0,2	R	3	2

PI 352453	Marsica 4	France	Unknown improvement status	80	95	0	HR	475,31	0,31	R	2	R	200	0,25	R	1	4
PI 428543	Mondur	France	Cultivar	90	115	4	MS	1279,01	0,83	S	4	MS	293,83	0,37	R	3	2
PI 306666	Montferrier	France	Cultivar	90	105	3	MR	851,23	0,55	MR	2	R	39,51	0,05	HR	2	-
PI 306667	Palastinien 20.606	France	Unknown improvement status	135	120	4	MS	622,22	0,4	MR	2	R	343,21	0,44	MR	2	-
PI 428544	Poinville	France	Cultivar	95	80	5	S	1101,85	0,72	MS	3	MR	274,07	0,35	R	3	-
PI 428545	Presto	France	Cultivar	85	130	5	S	816,67	0,53	MR	4	MS	303,7	0,39	R	3	-
PI 428546	Prolixe	France	Cultivar	120	90	4	MS	1075,93	0,7	MS	3	MR	251,85	0,32	R	3	-
PI 428547	Safari	France	Unknown improvement status	95	75	3	MR	967,9	0,63	MS	3	MR	558,02	0,71	MS	3	-
PI 352452	T 55	France	Unknown improvement status	90	95	1	HR	570,37	0,37	R	2	R	192,59	0,25	R	2	4
PI 352450	T 76 L2	France	Unknown improvement status	145	145	3	MR	505,56	0,33	R	5	S	745,68	0,95	S	2	2
PI 352451	T-2528	France	Unknown improvement status	125	120	0	HR	0	0	HR	2	R	948,15	1,21	S	3	2
PI 352454	T-2531	France	Unknown improvement status	100	95	4	MS	717,28	0,47	MR	2	R	128,4	0,16	HR	2	-
PI 428548	Tomclair	France	Cultivar	90	105	5	S	1326,54	0,86	S	4	MS	839,51	1,07	S	3	2
PI 584840	Villemur	France	Cultivar	90	140	4	MS	855,56	0,56	MR	NA	NA	312,35	0,4	R	2	-
PI 362226	Agathe	France	Cultivar	110	140	0	HR	69,14	0,04	HR	1	HR	118,52	0,15	HR	1	3
PI 306656	F8 31192-2	France	Breeding material	110	105	0	HR	0	0	HR	2	R	101,23	0,13	HR	1	4
PI 383411	33-9-5-6	France	Breeding material	115	110	0	HR	69,14	0,04	HR	NA	NA	285,19	0,36	R	1	4
PI 306653	F7 2008-1	France	Breeding material	115	105	0	HR	0	0	HR	2	R	64,2	0,08	HR	1	4
PI 306641	Bidi 17	France	Cultivar	120	100	2	R	337,04	0,22	R	0	HR	143,21	0,18	HR	1	4
PI 306654	F8-3012-1	France	Breeding material	120	110	1	HR	69,14	0,04	HR	1	HR	64,2	0,08	HR	1	4

PI 174699	Trigo Glutinoso	France	Unknown improvement status	120	145	1	HR	69,14	0,04	HR	3	MR	351,85	0,45	MR	1	-
PI 184539	3228	France	Cultivar	130	150	0	HR	0	0	HR	2	R	255,56	0,33	R	1	3
PI 48212	Rubio enlargado Atlemtege	France	Unknown improvement status	130	145	0	HR	0	0	HR	0	HR	118,52	0,15	HR	1	3
PI 174621	Douro Boukowo	France	Unknown improvement status	140	135	0	HR	0	0	HR	1	HR	144,44	0,18	HR	1	3
CItr 15160	Dimini Lesvon	Greece	Unknown improvement status	110	100	4	MS	734,57	0,48	MR	2	R	237,04	0,3	R	2	-
PI 352419	Lala	Greece	Unknown improvement status	120	130	4	MS	496,91	0,32	R	2	R	118,52	0,15	HR	2	1
PI 384039	Etit	Israel	Cultivar	75	85	2	R	246,3	0,16	HR	2	R	39,51	0,05	HR	1	4
PI 292031	Abu Fashit	Israel	Landrace	120	120	5	S	1127,78	0,73	MS	0	HR	138,27	0,18	HR	2	-
PI 430164	Line 78	Israel	Breeding material	100	90	4	MS	1451,85	0,94	S	3	MR	1096,3	1,4	S	3	2
PI 596616	V. 966131	Italy	Breeding material	65	135	0	HR	69,14	0,04	HR	1	HR	118,52	0,15	HR	1	4
PI 593006	V. 453	Italy	Breeding material	70	135	0	HR	69,14	0,04	HR	2	R	118,52	0,15	HR	1	4
PI 596620	V. 966527	Italy	Breeding material	70	125	0	HR	0	0	HR	1	HR	118,52	0,15	HR	1	4
PI 352462	Maliani 17	Italy	Cultivar	75	90	0	HR	203,09	0,13	HR	0	HR	118,52	0,15	HR	1	4
PI 596607	Near Isogenic: Awnless	Italy	Genetic material	75	115	2	R	69,14	0,04	HR	1	HR	118,52	0,15	HR	1	4
PI 596618	V. 966301	Italy	Breeding material	75	90	0	HR	0	0	HR	1	HR	118,52	0,15	HR	1	4
PI 596619	V. 966691	Italy	Breeding material	75	120	0	HR	0	0	HR	1	HR	118,52	0,15	HR	1	4
PI 596605	Near Isogenic: Awnless	Italy	Genetic material	90	140	2	R	69,14	0,04	HR	1	HR	118,52	0,15	HR	1	4
PI 596621	V. 967136	Italy	Breeding material	90	120	0	HR	0	0	HR	1	HR	118,52	0,15	HR	1	4
PI 322740	Maliani 11C	Italy	Breeding material	95	110	0	HR	0	0	HR	1	HR	135,8	0,17	HR	1	4
PI 596604	Near Isogenic: Awned	Italy	Genetic material	95	135	2	R	69,14	0,04	HR	1	HR	103,7	0,13	HR	1	4

PI 596608	Near Isogenic: Awned	Italy	Genetic material	95	100	0	HR	69,14	0,04	HR	1	HR	140,74	0,18	HR	1	4
PI 596612	Near Isogenic: Awned	Italy	Genetic material	95	120	0	HR	69,14	0,04	HR	1	HR	155,56	0,2	HR	1	4
PI 596599	Near Isogenic: Awnless	Italy	Genetic material	95	105	0	HR	246,3	0,16	HR	1	HR	103,7	0,13	HR	1	4
PI 596603	Near Isogenic: Awnless	Italy	Genetic material	95	100	2	R	69,14	0,04	HR	1	HR	118,52	0,15	HR	1	4
PI 596609	Near Isogenic: Awnless	Italy	Genetic material	95	100	2	R	69,14	0,04	HR	1	HR	155,56	0,2	HR	1	4
PI 593005	V. 433	Italy	Breeding material	95	130	0	HR	0	0	HR	2	R	118,52	0,15	HR	1	4
PI 358945	V. Montanari	Italy	Cultivar	95	115	0	HR	0	0	HR	0	HR	101,23	0,13	HR	1	4
PI 596598	Near Isogenic: Awned	Italy	Genetic material	100	95	0	HR	0	0	HR	1	HR	118,52	0,15	HR	1	4
PI 596600	Near Isogenic: Awned	Italy	Genetic material	100	105	2	R	246,3	0,16	HR	1	HR	103,7	0,13	HR	1	4
PI 596602	Near Isogenic: Awned	Italy	Genetic material	100	95	2	R	69,14	0,04	HR	1	HR	214,81	0,27	R	1	4
PI 596613	V. 966093	Italy	Breeding material	100	115	0	HR	69,14	0,04	HR	1	HR	155,56	0,2	HR	1	4
PI 596615	V. 966272	Italy	Breeding material	100	150	2	R	69,14	0,04	HR	1	HR	214,81	0,27	R	1	4
PI 415176	Valgerardo	Italy	Cultivar	100	125	0	HR	0	0	HR	2	R	348,15	0,44	MR	1	-
PI 157961	Bidi	Italy	Landrace	105	140	1	HR	69,14	0,04	HR	1	HR	214,81	0,27	R	1	4
PI 596610	Near Isogenic: Awned	Italy	Genetic material	105	95	2	R	69,14	0,04	HR	NA	NA	107,41	0,14	HR	1	4
PI 264945	1061	Italy	Landrace	115	120	4	MS	972,22	0,63	MS	3	MR	192,59	0,25	R	2	-
PI 157952	43892G	Italy	Unknown improvement status	110	120	3	MR	466,67	0,3	R	2	R	150,62	0,19	HR	2	4
CItr 15094	Alex 281	Italy	Cultivar	150	135	0	HR	406,17	0,26	R	3	MR	600	0,76	MS	2	-
PI 157921	Aziziah	Italy	Cultivar	100	105	3	MR	466,67	0,3	R	NA	NA	276,54	0,35	R	2	4
PI 94732	452	Italy	Landrace	110	120	0	HR	0	0	HR	NA	NA	296,3	0,38	R	1	4
PI 106814	Aziziah 17-45	Italy	Cultivar	100	135	0	HR	0	0	HR	1	HR	NA	NA	NA	-	-
PI 322737	Maliani 5B	Italy	Breeding material	110	120	1	HR	69,14	0,04	HR	1	HR	229,63	0,29	R	1	4
PI 593004	V. 429	Italy	Breeding material	110	120	0	HR	0	0	HR	2	R	214,81	0,27	R	1	4

PI 596614	V. 965936	Italy	Breeding material	110	120	2	R	69,14	0,04	HR	1	HR	118,52	0,15	HR	1	4
PI 370751	Capeiti Liguleless Mutant	Italy	Genetic material	135	125	3	MR	574,69	0,37	R	3	MR	285,19	0,36	R	2	1
PI 264949	Cappelli	Italy	Cultivar	120	125	4	MS	522,84	0,34	R	3	MR	301,23	0,38	R	2	1
PI 231363	Civitella	Italy	Landrace	120	125	4	MS	466,67	0,3	R	2	R	192,59	0,25	R	2	1
PI 370752	Ga B 125	Italy	Genetic material	125	130	4	MS	717,28	0,47	MR	3	MR	262,96	0,33	R	2	-
PI 367211	Geraardo 562	Italy	Breeding material	95	90	4	MS	1114,81	0,73	MS	2	R	376,54	0,48	MR	3	-
CItr 15114	Gerardo 463	Italy	Cultivar	80	80	3	MR	700	0,46	MR	2	R	523,46	0,67	MS	3	-
CItr 15115	Gerardo 464	Italy	Cultivar	70	75	5	S	1291,98	0,84	S	4	MS	314,81	0,4	MR	3	-
CItr 15116	Gerardo 469	Italy	Cultivar	80	75	3	MR	903,09	0,59	MR	3	MR	396,3	0,5	MR	3	-
CItr 15117	Gerardo 472	Italy	Cultivar	75	90	2	R	1028,4	0,67	MS	2	R	370,37	0,47	MR	3	-
CItr 15118	Gerardo 480	Italy	Cultivar	75	70	3	MR	1283,33	0,84	S	2	R	237,04	0,3	R	3	2
CItr 15119	Gerardo 481	Italy	Cultivar	100	80	3	MR	1326,54	0,86	S	NA	NA	192,59	0,25	R	3	2
PI 367208	Gerardo 513	Italy	Breeding material	120	140	2	R	764,81	0,5	MR	1	HR	140,74	0,18	HR	2	-
CItr 15255	Gerardo 514	Italy	Cultivar	100	85	3	MR	1235,8	0,8	S	NA	NA	671,6	0,85	S	3	2
CItr 15257	Gerardo 516	Italy	Cultivar	110	110	3	MR	941,98	0,61	MS	NA	NA	474,07	0,6	MS	3	-
CItr 15258	Gerardo 517	Italy	Cultivar	120	120	3	MR	846,91	0,55	MR	NA	NA	350,62	0,45	MR	2	-
CItr 15259	Gerardo 518	Italy	Cultivar	85	90	4	MS	1240,12	0,81	S	1	HR	840,74	1,07	S	3	2
PI 367209	Gerardo 521	Italy	Breeding material	95	100	4	MS	1136,42	0,74	MS	3	MR	688,89	0,88	S	3	-
PI 367210	Gerardo 522	Italy	Breeding material	95	90	4	MS	1136,42	0,74	MS	3	MR	553,09	0,7	MS	3	-
PI 367213	Gerardo 573	Italy	Breeding material	80	75	3	MR	574,69	0,37	R	1	HR	407,41	0,52	MR	3	-
PI 367214	Gerardo 589	Italy	Breeding material	90	95	4	MS	877,16	0,57	MR	2	R	440,74	0,56	MR	3	-
PI 367215	Gerardo 592	Italy	Breeding material	90	85	2	R	540,12	0,35	R	2	R	262,96	0,33	R	3	4
PI 367216	Gerardo 594	Italy	Breeding material	90	105	3	MR	570,37	0,37	R	4	MS	351,85	0,45	MR	3	-
PI 367217	Gerardo 612	Italy	Breeding material	95	85	4	MS	816,67	0,53	MR	3	MR	459,26	0,58	MR	3	-

PI 367218	Gerardo 616	Italy	Breeding material	100	80	4	MS	877,16	0,57	MR	3	MR	459,26	0,58	MR	3	-
PI 367219	Gerardo 618	Italy	Breeding material	80	75	4	MS	734,57	0,48	MR	2	R	262,96	0,33	R	3	-
PI 367220	Gerardo 619	Italy	Breeding material	85	75	3	MR	898,77	0,58	MR	1	HR	718,52	0,91	S	3	-
PI 367221	Gerardo 620	Italy	Breeding material	85	90	3	MR	687,04	0,45	MR	2	R	748,15	0,95	S	3	-
PI 367222	Gerardo 622	Italy	Breeding material	100	85	2	R	708,64	0,46	MR	3	MR	285,19	0,36	R	3	-
PI 367223	Gerardo 623	Italy	Breeding material	85	85	2	R	1088,89	0,71	MS	NA	NA	259,26	0,33	R	3	-
PI 520030	Gerardo 667	Italy	Cultivar	90	85	4	MS	1162,35	0,76	MS	3	MR	370,37	0,47	MR	3	-
CItr 15214	Giorgio 298	Italy	Cultivar	70	70	2	R	959,26	0,62	MS	NA	NA	292,59	0,37	R	3	-
CItr 15148	Giorgio 317	Italy	Cultivar	80	80	4	MS	1291,98	0,84	S	3	MR	1106,17	1,41	S	3	2
CItr 15149	Giorgio 324	Italy	Cultivar	80	70	4	MS	1326,54	0,86	S	3	MR	948,15	1,21	S	3	2
CItr 15251	Giorgio 328	Italy	Cultivar	130	125	3	MR	743,21	0,48	MR	2	R	459,26	0,58	MR	2	-
CItr 15108	Giorgio 331	Italy	Cultivar	130	135	3	MR	483,95	0,31	R	3	MR	303,7	0,39	R	2	1
CItr 15150	Giorgio 360	Italy	Cultivar	80	75	4	MS	864,2	0,56	MR	4	MS	866,67	1,1	S	3	-
CItr 15151	Giorgio 378	Italy	Cultivar	120	135	2	R	475,31	0,31	R	3	MR	697,53	0,89	S	3	2
CItr 15109	Giorgio 380	Italy	Cultivar	90	90	3	MR	1136,42	0,74	MS	3	MR	143,21	0,18	HR	3	-
CItr 15152	Giorgio 381	Italy	Cultivar	80	90	3	MR	613,58	0,4	R	3	MR	646,91	0,82	S	3	2
CItr 15215	Giorgio 386	Italy	Cultivar	80	80	3	MR	1361,11	0,89	S	1	HR	888,89	1,13	S	3	2
CItr 15252	Giorgio 391	Italy	Cultivar	80	90	4	MS	1127,78	0,73	MS	1	HR	925,93	1,18	S	3	-
CItr 15111	Giorgio 396	Italy	Cultivar	80	85	2	R	1037,04	0,67	MS	3	MR	948,15	1,21	S	3	-
CItr 15216	Giorgio 445	Italy	Cultivar	80	75	3	MR	868,52	0,57	MR	2	R	474,07	0,6	MS	3	-
CItr 15253	Giorgio 446	Italy	Cultivar	80	90	3	MR	998,15	0,65	MS	2	R	959,26	1,22	S	3	-
PI 367226	Giorgio 446	Italy	Breeding material	90	75	0	HR	617,9	0,4	MR	NA	NA	233,33	0,3	R	3	-
CItr 15112	Giorgio 448	Italy	Cultivar	80	70	3	MR	1062,96	0,69	MS	0	HR	285,19	0,36	R	3	-
CItr 15218	Giorgio 449	Italy	Cultivar	90	60	4	MS	1443,21	0,94	S	2	R	748,15	0,95	S	3	2
PI 367228	Giorgio 449	Italy	Breeding material	100	75	4	MS	1132,1	0,74	MS	NA	NA	118,52	0,15	HR	3	-
CItr 15113	Giorgio 450	Italy	Cultivar	80	70	4	MS	1317,9	0,86	S	2	R	296,3	0,38	R	3	2

CItr 15254	Giorgio 454	Italy	Cultivar	80	75	3	MR	1201,23	0,78	MS	2	R	251,85	0,32	R	3	-
PI 367230	Giorgio 454	Italy	Breeding material	85	75	4	MS	1075,93	0,7	MS	NA	NA	251,85	0,32	R	3	-
PI 367233	Giorgio 613	Italy	Breeding material	80	80	3	MR	795,06	0,52	MR	2	R	395,06	0,5	MR	3	-
PI 367234	Giorgio 614	Italy	Breeding material	90	80	3	MR	859,88	0,56	MR	2	R	474,07	0,6	MS	3	-
PI 367235	Giorgio 615	Italy	Breeding material	90	75	2	R	738,89	0,48	MR	NA	NA	534,57	0,68	MS	3	-
PI 367236	Giorgio 617	Italy	Breeding material	80	75	4	MS	734,57	0,48	MR	NA	NA	585,19	0,74	MS	3	-
PI 367237	Giorgio 621	Italy	Breeding material	75	80	4	MS	985,19	0,64	MS	NA	NA	459,26	0,58	MR	3	-
PI 503555	Icaro	Italy	Cultivar	140	105	3	MR	350	0,23	R	2	R	225,93	0,29	R	2	1
PI 470759	MG 4469	Italy	Landrace	120	100	4	MS	635,19	0,41	MR	1	HR	192,59	0,25	R	2	-
PI 470760	MG 4470	Italy	Landrace	170	150	3	MR	466,67	0,3	R	1	HR	118,52	0,15	HR	2	1
PI 470761	MG 4471	Italy	Landrace	130	110	2	R	311,11	0,2	R	1	HR	286,42	0,36	R	2	1
PI 470762	MG 4472	Italy	Landrace	135	100	4	MS	583,33	0,38	R	1	HR	155,56	0,2	HR	2	1
PI 470765	MG 4475	Italy	Landrace	105	115	4	MS	591,98	0,39	R	2	R	239,51	0,3	R	2	4
PI 470766	MG 4476	Italy	Landrace	130	130	3	MR	371,6	0,24	R	2	R	255,56	0,33	R	2	1
PI 470767	MG 4477	Italy	Landrace	140	105	3	MR	613,58	0,4	R	2	R	118,52	0,15	HR	2	1
PI 470768	MG 4478	Italy	Landrace	130	135	3	MR	397,53	0,26	R	2	R	155,56	0,2	HR	2	1
PI 470769	MG 4479	Italy	Landrace	130	135	0	HR	350	0,23	R	2	R	143,21	0,18	HR	2	1
PI 470776	MG 4486	Italy	Landrace	160	145	2	R	453,7	0,3	R	3	MR	174,07	0,22	R	2	1
PI 192732	Aziziah II	Italy	Cultivar	115	120	3	MR	466,67	0,3	R	1	HR	64,2	0,08	HR	1	4
PI 322742	Maliani 16A	Italy	Breeding material	115	130	0	HR	0	0	HR	2	R	143,21	0,18	HR	1	3
PI 596622	V. 967291	Italy	Breeding material	115	115	0	HR	0	0	HR	1	HR	118,52	0,15	HR	1	4
PI 157977	Pavone	Italy	Landrace	140	115	0	HR	0	0	HR	1	HR	474,07	0,6	MS	1	-
PI 264950	Pavone	Italy	Landrace	130	145	0	HR	250,62	0,16	HR	3	MR	251,85	0,32	R	2	1
PI 191471	Razza 181	Italy	Unknown improvement status	135	135	5	S	604,94	0,39	R	5	S	103,7	0,13	HR	2	1
PI 157973	Russello	Italy	Landrace	140	105	3	MR	328,4	0,21	R	1	HR	296,3	0,38	R	2	1

PI 157922	Sabaudia	Italy	Unknown improvement status	120	105	2	R	350	0,23	R	2	R	592,59	0,75	MS	3	-
PI 157972	Semenzella	Italy	Landrace	135	115	4	MS	535,8	0,35	R	1	HR	192,59	0,25	R	2	1
CItr 15169	Sincape 9	Italy	Cultivar	110	95	4	MS	717,28	0,47	MR	2	R	314,81	0,4	MR	3	-
PI 231358	Timilia SG 1	Italy	Cultivar	110	120	4	MS	712,96	0,46	MR	1	HR	613,58	0,78	MS	3	-
CItr 15277	Tonus	Italy	Unknown improvement status	120	140	5	S	717,28	0,47	MR	NA	NA	155,56	0,2	HR	2	-
PI 157959	Tripolino	Italy	Unknown improvement status	100	130	3	MR	535,8	0,35	R	1	HR	118,52	0,15	HR	2	4
PI 157967	Tunisina	Italy	Unknown improvement status	140	125	3	MR	466,67	0,3	R	2	R	288,89	0,37	R	2	1
PI 520274	V 910	Italy	Breeding material	110	110	2	R	769,14	0,5	MR	2	R	143,21	0,18	HR	2	-
PI 520275	V 922	Italy	Breeding material	85	85	4	MS	1546,91	1,01	S	3	MR	802,47	1,02	S	3	2
PI 593000	V. 40113A	Italy	Breeding material	95	90	4	MS	1343,83	0,87	S	3	MR	281,48	0,36	R	3	2
PI 593001	V. 40113B	Italy	Breeding material	85	75	4	MS	1585,8	1,03	S	3	MR	237,04	0,3	R	3	2
PI 584655	V. 7051	Italy	Breeding material	135	110	4	MS	505,56	0,33	R	2	R	214,81	0,27	R	2	1
PI 593002	V. 903-5	Italy	Breeding material	95	85	4	MS	1261,73	0,82	S	2	R	637,04	0,81	S	3	2
PI 593003	V. 903-9	Italy	Breeding material	90	125	3	MR	1456,17	0,95	S	2	R	686,42	0,87	S	3	2
PI 584657	V. 9054	Italy	Breeding material	95	90	4	MS	1343,83	0,87	S	3	MR	451,85	0,58	MR	3	-
PI 584658	V. 9132	Italy	Breeding material	95	80	4	MS	1274,69	0,83	S	3	MR	871,6	1,11	S	3	2
PI 191438	Aziziah 17-45	Italy	Cultivar	120	125	0	HR	0	0	HR	1	HR	103,7	0,13	HR	1	3
CItr 15147	Valaniene	Italy	Cultivar	85	75	5	S	1123,46	0,73	MS	NA	NA	451,85	0,58	MR	3	-
PI 367231	Valfiora	Italy	Breeding material	80	75	4	MS	1136,42	0,74	MS	NA	NA	313,58	0,4	R	3	-
PI 192025	Bidi	Italy	Landrace	120	110	0	HR	0	0	HR	1	HR	64,2	0,08	HR	1	4
PI 191766	Edda	Italy	Cultivar	120	120	0	HR	0	0	HR	0	HR	103,7	0,13	HR	1	4

CItr 15217	Valgiorgio	Italy	Cultivar	80	70	4	MS	777,78	0,51	MR	NA	NA	581,48	0,74	MS	3	-
PI 367238	Vallega Zitelli 609	Italy	Cultivar	95	90	2	R	730,25	0,48	MR	2	R	834,57	1,06	S	3	-
PI 367239	Vallega Zitelli 610	Italy	Cultivar	85	85	3	MR	881,48	0,57	MR	3	MR	459,26	0,58	MR	3	-
CItr 15260	Vallega Zittelli 456	Italy	Breeding material	120	140	3	MR	712,96	0,46	MR	2	R	474,07	0,6	MS	2	-
CItr 15261	Vallega Zittelli 457	Italy	Breeding material	120	135	4	MS	980,86	0,64	MS	3	MR	592,59	0,75	MS	3	-
CItr 15263	Vallega Zittelli 459	Italy	Breeding material	115	115	5	S	661,11	0,43	MR	3	MR	281,48	0,36	R	2	-
CItr 15264	Vallega Zittelli 460	Italy	Breeding material	140	140	3	MR	466,67	0,3	R	1	HR	274,07	0,35	R	2	1
CItr 15096	Vallega Zittelli 482	Italy	Breeding material	130	135	2	R	362,96	0,24	R	3	MR	274,07	0,35	R	2	1
CItr 15097	Vallega Zittelli 483	Italy	Breeding material	120	125	5	S	635,19	0,41	MR	2	R	311,11	0,4	R	2	-
CItr 15098	Vallega Zittelli 484	Italy	Breeding material	130	130	4	MS	535,8	0,35	R	3	MR	350,62	0,45	MR	2	-
CItr 15099	Vallega Zittelli 485	Italy	Breeding material	130	140	3	MR	436,42	0,28	R	5	S	351,85	0,45	MR	2	-
CItr 15100	Vallega Zittelli 486	Italy	Breeding material	130	135	3	MR	561,73	0,37	R	4	MS	229,63	0,29	R	2	1
PI 322741	Maliani 12D	Italy	Breeding material	120	115	0	HR	0	0	HR	1	HR	118,52	0,15	HR	1	4
CItr 15102	Vallega Zittelli 488	Italy	Breeding material	110	135	3	MR	527,16	0,34	R	3	MR	451,85	0,58	MR	2	-
CItr 15103	Vallega Zittelli 489	Italy	Breeding material	105	125	3	MR	445,06	0,29	R	2	R	637,04	0,81	S	3	2
CItr 15104	Vallega Zittelli 490	Italy	Breeding material	120	105	2	R	445,06	0,29	R	NA	NA	325,93	0,41	MR	2	-
CItr 15105	Vallega Zittelli 491	Italy	Breeding material	120	120	2	R	350	0,23	R	NA	NA	155,56	0,2	HR	1	4
CItr 15106	Vallega Zittelli 492	Italy	Breeding material	120	120	3	MR	535,8	0,35	R	2	R	556,79	0,71	MS	3	-
PI 157979	Vallelunga Glabra	Italy	Unknown improvement status	140	120	0	HR	0	0	HR	0	HR	NA	NA	NA	-	-
CItr 15110	Valloriolo	Italy	Cultivar	80	85	2	R	652,47	0,42	MR	4	MS	617,28	0,79	MS	3	-
PI 367232	Valnera	Italy	Breeding material	80	70	4	MS	838,27	0,55	MR	NA	NA	390,12	0,5	MR	3	-

PI 415177	Valnova	Italy	Cultivar	95	85	4	MS	959,26	0,62	MS	NA	NA	429,63	0,55	MR	3	-
PI 367229	Valsacco	Italy	Breeding material	95	80	4	MS	1231,48	0,8	S	1	HR	686,42	0,87	S	3	2
PI 367212	Valselva	Italy	Breeding material	90	85	3	MR	708,64	0,46	MR	3	MR	617,28	0,79	MS	3	-
CItr 15183	Valtarquinio	Italy	Cultivar	85	75	4	MS	717,28	0,47	MR	1	HR	601,23	0,77	MS	3	-
PI 367225	Valtarquinio	Italy	Breeding material	90	75	0	HR	643,83	0,42	MR	NA	NA	318,52	0,41	MR	3	-
PI 271894	Aziziah	Italy	Cultivar	125	140	2	R	263,58	0,17	HR	0	HR	103,7	0,13	HR	1	3
PI 596597	Near Isogenic: Awnless	Italy	Genetic material	125	100	0	HR	0	0	HR	1	HR	118,52	0,15	HR	1	4
PI 157950	13892C	Italy	Unknown improvement status	130	135	2	R	259,26	0,17	HR	2	R	155,56	0,2	HR	1	1
PI 264942	22a	Italy	Landrace	130	115	0	HR	0	0	HR	2	R	192,59	0,25	R	1	3
PI 352430	Capeiti 8	Italy	Cultivar	130	150	0	HR	0	0	HR	1	HR	237,04	0,3	R	1	3
PI 157954	Francesa	Italy	Landrace	130	145	0	HR	0	0	HR	1	HR	143,21	0,18	HR	1	3
PI 191108	Garigliano	Italy	Cultivar	130	175	0	HR	0	0	HR	1	HR	111,11	0,14	HR	1	3
PI 157956	Gigante	Italy	Landrace	130	130	1	HR	69,14	0,04	HR	1	HR	214,81	0,27	R	1	3
CItr 6888	King Emanuel	Italy	Unknown improvement status	130	130	3	MR	324,07	0,21	R	2	R	155,56	0,2	HR	1	1
PI 157966	Scavuzza	Italy	Landrace	130	125	0	HR	0	0	HR	2	R	327,16	0,42	MR	1	-
CItr 15101	Vallega Zittelli 487	Italy	Breeding material	130	125	1	HR	69,14	0,04	HR	2	R	255,56	0,33	R	1	3
PI 191988	Batisti	Italy	Unknown improvement status	135	145	0	HR	0	0	HR	0	HR	135,8	0,17	HR	1	3
PI 264958	Farro Lungo	Italy	Landrace	135	150	0	HR	0	0	HR	1	HR	230,86	0,29	R	1	3
PI 157955	Francesone	Italy	Landrace	135	135	0	HR	0	0	HR	1	HR	213,58	0,27	R	1	3
PI 352417	Garigliano	Italy	Cultivar	135	125	0	HR	69,14	0,04	HR	1	HR	237,04	0,3	R	1	3
PI 322736	Maliani 4B	Italy	Breeding material	135	150	0	HR	0	0	HR	1	HR	174,07	0,22	R	1	3
PI 470772	MG 4482	Italy	Landrace	135	145	0	HR	0	0	HR	2	R	118,52	0,15	HR	1	3
PI 231380	Saragolla	Italy	Landrace	135	150	0	HR	0	0	HR	2	R	155,56	0,2	HR	1	3

PI 231361	Brottu	Italy	Landrace	140	135	0	HR	0	0	HR	2	R	274,07	0,35	R	1	3
PI 157984	Bufala Rossa Lunga	Italy	Landrace	140	120	2	R	298,15	0,19	HR	2	R	204,94	0,26	R	2	1
PI 157981	Castiglione Pubescente	Italy	Landrace	140	125	0	HR	0	0	HR	2	R	196,3	0,25	R	1	3
PI 157975	Cotrone	Italy	Unknown improvement status	140	100	0	HR	0	0	HR	1	HR	419,75	0,53	MR	1	-
PI 157969	Farro Lungo	Italy	Landrace	140	125	0	HR	0	0	HR	1	HR	155,56	0,2	HR	1	3
PI 157976	Gioia	Italy	Landrace	140	120	0	HR	0	0	HR	1	HR	118,52	0,15	HR	1	3
PI 157970	Giustalisa	Italy	Landrace	140	130	0	HR	0	0	HR	2	R	237,04	0,3	R	1	3
PI 470764	MG 4474	Italy	Landrace	140	135	2	R	69,14	0,04	HR	2	R	118,52	0,15	HR	1	3
PI 470774	MG 4484	Italy	Landrace	140	150	1	HR	69,14	0,04	HR	1	HR	98,77	0,13	HR	1	3
PI 470775	MG 4485	Italy	Landrace	140	140	1	HR	241,98	0,16	HR	2	R	103,7	0,13	HR	1	3
PI 470777	MG 4487	Italy	Landrace	140	140	0	HR	69,14	0,04	HR	2	R	118,52	0,15	HR	1	3
PI 264956	Regina	Italy	Cultivar	140	170	0	HR	0	0	HR	2	R	262,96	0,33	R	1	3
PI 157978	Ruscia	Italy	Landrace	140	110	0	HR	0	0	HR	1	HR	296,3	0,38	R	1	3
PI 352418	Sabaudia	Italy	Unknown improvement status	140	140	0	HR	0	0	HR	0	HR	155,56	0,2	HR	1	3
PI 157958	Sammartinara	Italy	Landrace	140	125	1	HR	69,14	0,04	HR	2	R	262,96	0,33	R	1	3
PI 157982	Scorsonera	Italy	Landrace	140	120	2	R	350	0,23	R	1	HR	118,52	0,15	HR	1	1
PI 264955	Scorzonora	Italy	Unknown improvement status	140	135	0	HR	0	0	HR	2	R	162,96	0,21	R	1	3
PI 157965	Trentino	Italy	Cultivar	140	155	0	HR	0	0	HR	2	R	118,52	0,15	HR	1	3
PI 157980	Vallelunga Pubescente	Italy	Unknown improvement status	140	90	0	HR	0	0	HR	2	R	262,96	0,33	R	1	4
CItr 12452	Capelli 38	Italy	Cultivar	150	140	0	HR	241,98	0,16	HR	2	R	143,21	0,18	HR	1	1
PI 191209	Sambaudia	Italy	Unknown improvement status	150	170	0	HR	0	0	HR	2	R	159,26	0,2	R	1	3
PI 232815	San Giorgio	Italy	Cultivar	150	170	0	HR	0	0	HR	2	R	200	0,25	R	1	3
PI 584656	V. 9104	Italy	Breeding	150	120	0	HR	0	0	HR	2	R	279,01	0,36	R	1	3

			material														
PI 367207	Valgerardo	Italy	Breeding material	150	150	0	HR	0	0	HR	1	HR	237,04	0,3	R	1	3
PI 470771	MG 4481	Italy	Landrace	160	120	0	HR	0	0	HR	2	R	118,52	0,15	HR	1	3
PI 210879	F-8	Jordan	Unknown improvement status	120	140	0	HR	0	0	HR	0	HR	101,23	0,13	HR	1	3
PI 420939	12	Jordan	Landrace	95	110	3	MR	1045,68	0,68	MS	4	MS	844,44	1,07	S	3	-
PI 371821	Diera Alla 1	Jordan	Unknown improvement status	95	105	2	R	587,65	0,38	R	2	R	101,23	0,13	HR	2	4
PI 428468	Jordan 38	Jordan	Breeding material	110	100	1	HR	350	0,23	R	2	R	214,81	0,27	R	1	4
PI 234380	Durz Karaki	Jordan	Unknown improvement status	135	130	0	HR	0	0	HR	2	R	143,21	0,18	HR	1	3
PI 223150	Auttma White	Jordan	Landrace	140	130	0	HR	0	0	HR	2	R	237,04	0,3	R	1	3
PI 191630	Marrocos	Morocco	Unknown improvement status	95	135	2	R	246,3	0,16	HR	1	HR	209,88	0,27	R	1	4
PI 184528	Marrocos 110	Morocco	Unknown improvement status	120	150	0	HR	0	0	HR	0	HR	159,26	0,2	R	1	3
PI 192514	Marrocos	Morocco	Unknown improvement status	130	165	0	HR	0	0	HR	3	MR	255,56	0,33	R	1	3
PI 192635	2270A	Morocco	Breeding material	135	145	0	HR	0	0	HR	0	HR	140,74	0,18	HR	1	3
PI 191405	Recio del Pais	Morocco	Landrace	135	145	0	HR	0	0	HR	1	HR	83,95	0,11	HR	1	3
PI 192482	2023A	Morocco	Unknown improvement status	140	150	2	R	272,22	0,18	HR	0	HR	103,7	0,13	HR	1	3
PI 191029	Ble Dur 250	Morocco	Landrace	140	145	0	HR	0	0	HR	2	R	101,23	0,13	HR	1	3
PI 191621	Marrocos	Morocco	Unknown improvement status	150	145	1	HR	69,14	0,04	HR	1	HR	288,89	0,37	R	1	3
PI 191812	HN ROD 51 14874	Portugal	Unknown improvement status	90	125	0	HR	0	0	HR	0	HR	214,81	0,27	R	1	4

PI 134942	2860	Portugal	Landrace	95	110	0	HR	358,64	0,23	R	0	HR	197,53	0,25	R	1	4
PI 191785	H 22 E 13381	Portugal	Unknown improvement status	95	130	0	HR	0	0	HR	2	R	264,2	0,34	R	1	4
PI 192835	Candeal de Grao Escuro	Portugal	Landrace	100	100	3	MR	432,1	0,28	R	2	R	148,15	0,19	HR	1	4
PI 192837	Candeal de Grao Escuro	Portugal	Landrace	100	110	4	MS	466,67	0,3	R	1	HR	101,23	0,13	HR	1	4
PI 191775	HN ROD 28 13775	Portugal	Unknown improvement status	100	120	0	HR	0	0	HR	0	HR	140,74	0,18	HR	1	4
PI 270133	Preto Amarelo	Portugal	Landrace	100	95	2	R	259,26	0,17	HR	3	MR	175,31	0,22	R	1	4
PI 56256	Tremez Preto	Portugal	Landrace	100	110	3	MR	272,22	0,18	HR	0	HR	119,75	0,15	HR	1	4
PI 134906	2847	Portugal	Landrace	105	95	0	HR	0	0	HR	4	MS	407,41	0,52	MR	1	-
PI 134944	2865	Portugal	Landrace	105	150	0	HR	0	0	HR	0	HR	190,12	0,24	R	1	3
PI 192436	Alentejo	Portugal	Landrace	105	130	0	HR	0	0	HR	0	HR	103,7	0,13	HR	1	4
PI 191850	HN ROD 46 14869	Portugal	Unknown improvement status	105	140	0	HR	0	0	HR	1	HR	103,7	0,13	HR	1	3
PI 134950	Iodurum	Portugal	Landrace	105	125	2	R	69,14	0,04	HR	0	HR	101,23	0,13	HR	1	4
PI 134943	2863	Portugal	Landrace	110	100	0	HR	0	0	HR	0	HR	159,26	0,2	R	1	4
PI 134933	2870	Portugal	Landrace	110	125	1	HR	69,14	0,04	HR	0	HR	140,74	0,18	HR	1	4
PI 134934	2871	Portugal	Landrace	110	100	1	HR	69,14	0,04	HR	0	HR	101,23	0,13	HR	1	4
PI 134935	2874	Portugal	Landrace	110	110	1	HR	69,14	0,04	HR	0	HR	140,74	0,18	HR	1	4
PI 192440	Alexandre	Portugal	Landrace	110	130	0	HR	0	0	HR	1	HR	103,7	0,13	HR	1	4
PI 192441	Corado	Portugal	Landrace	110	125	0	HR	0	0	HR	0	HR	103,7	0,13	HR	1	4
PI 204053	Espanhol	Portugal	Landrace	110	100	0	HR	0	0	HR	2	R	101,23	0,13	HR	1	4
PI 191811	H M3 12232	Portugal	Unknown improvement status	110	165	1	HR	69,14	0,04	HR	0	HR	103,7	0,13	HR	1	3
PI 191788	HN A 12241	Portugal	Unknown improvement status	110	120	0	HR	0	0	HR	0	HR	103,7	0,13	HR	1	4
PI 191786	HN ROD 13 13758	Portugal	Unknown improvement status	110	160	0	HR	0	0	HR	2	R	200	0,25	R	1	3

PI 191779	HN ROD 24 13769	Portugal	Unknown improvement status	110	120	0	HR	0	0	HR	0	HR	103,7	0,13	HR	1	4
PI 191809	HN ROD 43 14866	Portugal	Unknown improvement status	110	130	0	HR	0	0	HR	0	HR	103,7	0,13	HR	1	4
PI 192442	Rapazinho	Portugal	Landrace	110	145	0	HR	0	0	HR	1	HR	103,7	0,13	HR	1	3
PI 191717	Tremes Preto	Portugal	Landrace	110	125	4	MS	350	0,23	R	1	HR	200	0,25	R	1	4
PI 204031	Tremes Rijo	Portugal	Landrace	110	125	0	HR	0	0	HR	0	HR	103,7	0,13	HR	1	4
PI 184642	Verdeal Rijo	Portugal	Landrace	110	120	0	HR	0	0	HR	1	HR	279,01	0,36	R	1	4
PI 94721	429	Portugal	Landrace	115	125	2	R	350	0,23	R	1	HR	64,2	0,08	HR	1	4
PI 134910	2912	Portugal	Landrace	115	145	0	HR	0	0	HR	1	HR	140,74	0,18	HR	1	3
PI 56244	Aza de Corvo	Portugal	Landrace	115	125	0	HR	0	0	HR	0	HR	103,7	0,13	HR	1	4
PI 185725	Canoco	Portugal	Landrace	115	150	0	HR	0	0	HR	2	R	64,2	0,08	HR	1	3
PI 191858	H 22 A 13375	Portugal	Unknown improvement status	115	150	0	HR	246,3	0,16	HR	0	HR	103,7	0,13	HR	1	3
PI 191771	H N ROD 26 13771	Portugal	Unknown improvement status	115	140	0	HR	0	0	HR	0	HR	103,7	0,13	HR	1	3
PI 184544	Hibrido natural Rod 26	Portugal	Unknown improvement status	115	145	0	HR	0	0	HR	0	HR	155,56	0,2	HR	1	3
PI 191801	HN ROD 14 13759	Portugal	Unknown improvement status	115	140	0	HR	0	0	HR	0	HR	103,7	0,13	HR	1	3
PI 191784	HN ROD 18 13763	Portugal	Unknown improvement status	115	140	0	HR	0	0	HR	0	HR	103,7	0,13	HR	1	3
PI 191629	HN ROD 48 14871	Portugal	Unknown improvement status	115	130	3	MR	293,83	0,19	HR	0	HR	103,7	0,13	HR	1	4
PI 56246	Jarardo	Portugal	Landrace	115	115	1	HR	69,14	0,04	HR	0	HR	64,2	0,08	HR	1	4
PI 191949	Lobiero	Portugal	Landrace	115	140	0	HR	0	0	HR	0	HR	103,7	0,13	HR	1	3
PI 192446	Mongia	Portugal	Landrace	115	135	0	HR	0	0	HR	1	HR	103,7	0,13	HR	1	3
PI 184643	Mourisco Preto de Grao	Portugal	Landrace	115	125	0	HR	0	0	HR	0	HR	160,49	0,2	R	1	4

	Escure																
PI 192849	Mourisco Preto de Grao Escuro	Portugal	Landrace	115	130	2	R	246,3	0,16	HR	1	HR	197,53	0,25	R	1	4
PI 56250	Pombinho	Portugal	Landrace	115	130	0	HR	0	0	HR	0	HR	140,74	0,18	HR	1	3
PI 56252	Rubiao	Portugal	Landrace	115	120	2	R	298,15	0,19	HR	1	HR	60,49	0,08	HR	1	4
PI 192830	Russo	Portugal	Landrace	115	145	0	HR	0	0	HR	2	R	103,7	0,13	HR	1	3
PI 192420	Santa Marta	Portugal	Landrace	115	125	0	HR	0	0	HR	0	HR	103,7	0,13	HR	1	4
PI 204038	Vermelejoilo	Portugal	Landrace	115	125	1	HR	69,14	0,04	HR	2	R	103,7	0,13	HR	1	4
PI 94728	442	Portugal	Landrace	120	135	0	HR	0	0	HR	1	HR	103,7	0,13	HR	1	3
PI 134925	2837	Portugal	Landrace	120	135	0	HR	0	0	HR	1	HR	140,74	0,18	HR	1	3
PI 134922	2854	Portugal	Landrace	120	155	2	R	246,3	0,16	HR	1	HR	177,78	0,23	R	1	3
PI 134923	2858	Portugal	Landrace	120	140	0	HR	0	0	HR	1	HR	159,26	0,2	R	1	3
PI 134917	2880	Portugal	Landrace	120	160	2	R	337,04	0,22	R	1	HR	159,26	0,2	R	2	1
PI 134940	2887	Portugal	Landrace	120	140	0	HR	0	0	HR	0	HR	159,26	0,2	R	1	3
PI 134919	2898	Portugal	Landrace	120	135	1	HR	69,14	0,04	HR	1	HR	174,07	0,22	R	1	3
PI 134931	2906	Portugal	Landrace	120	130	2	R	337,04	0,22	R	1	HR	119,75	0,15	HR	1	4
PI 134909	2910	Portugal	Landrace	120	125	0	HR	0	0	HR	1	HR	233,33	0,3	R	1	4
PI 134911	2915	Portugal	Landrace	120	125	2	R	298,15	0,19	HR	2	R	233,33	0,3	R	2	4
PI 134914	2917	Portugal	Landrace	120	150	2	R	298,15	0,19	HR	1	HR	119,75	0,15	HR	1	3
PI 191865	3250	Portugal	Breeding material	120	135	0	HR	0	0	HR	0	HR	64,2	0,08	HR	1	3
PI 192157	3585	Portugal	Unknown improvement status	120	135	1	HR	69,14	0,04	HR	1	HR	103,7	0,13	HR	1	3
PI 134905	2843	Portugal	Landrace	105	130	3	MR	449,38	0,29	R	3	MR	318,52	0,41	MR	2	-
PI 56264	Alexandre	Portugal	Landrace	120	150	0	HR	0	0	HR	2	R	140,74	0,18	HR	1	3
PI 134907	2850	Portugal	Landrace	115	130	4	MS	583,33	0,38	R	4	MS	76,54	0,1	HR	2	1
PI 134908	2851	Portugal	Landrace	115	140	4	MS	583,33	0,38	R	1	HR	64,2	0,08	HR	2	1
PI 56243	Anafil	Portugal	Landrace	120	135	0	HR	0	0	HR	0	HR	91,36	0,12	HR	1	3
PI 185733	Asa de Corvo	Portugal	Landrace	120	155	0	HR	0	0	HR	0	HR	140,74	0,18	HR	1	3

PI 204022	Barba de Lobo	Portugal	Landrace	120	130	0	HR	0	0	HR	0	HR	118,52	0,15	HR	1	3
PI 204009	Branco	Portugal	Landrace	120	130	0	HR	0	0	HR	1	HR	64,2	0,08	HR	1	3
PI 185736	Candeal	Portugal	Landrace	120	165	0	HR	0	0	HR	0	HR	140,74	0,18	HR	1	3
PI 204025	Candeal	Portugal	Landrace	120	100	0	HR	0	0	HR	2	R	122,22	0,16	HR	1	4
PI 56259	CItr 7068	Portugal	Breeding material	120	155	2	R	246,3	0,16	HR	2	R	118,52	0,15	HR	1	3
PI 204023	Dezassete	Portugal	Landrace	120	120	0	HR	0	0	HR	0	HR	118,52	0,15	HR	1	4
PI 56255	Durazio Mollor	Portugal	Landrace	120	120	4	MS	328,4	0,21	R	1	HR	101,23	0,13	HR	1	4
PI 56254	Durazio Rigo	Portugal	Landrace	120	135	3	MR	466,67	0,3	R	1	HR	64,2	0,08	HR	2	1
PI 192853	Durazio Rijo	Portugal	Landrace	120	125	0	HR	0	0	HR	1	HR	140,74	0,18	HR	1	3
PI 185743	Durazio Rijo Glabro	Portugal	Landrace	120	145	0	HR	0	0	HR	1	HR	140,74	0,18	HR	1	3
PI 191655	Espanhol	Portugal	Landrace	120	155	0	HR	0	0	HR	2	R	140,74	0,18	HR	1	3
PI 191876	H N F 12446	Portugal	Unknown improvement status	120	160	0	HR	69,14	0,04	HR	0	HR	122,22	0,16	HR	1	3
PI 184545	Hibrido natural Rod 37	Portugal	Unknown improvement status	120	130	0	HR	0	0	HR	0	HR	155,56	0,2	HR	1	3
PI 191856	HN G 12447	Portugal	Unknown improvement status	120	145	0	HR	0	0	HR	0	HR	103,7	0,13	HR	1	3
PI 191776	HN ROD 25 13770	Portugal	Unknown improvement status	120	120	0	HR	0	0	HR	0	HR	118,52	0,15	HR	1	4
PI 134920	2890	Portugal	Landrace	120	140	2	R	453,7	0,3	R	1	HR	140,74	0,18	HR	2	1
PI 191764	HN ROD 37 13784	Portugal	Unknown improvement status	120	145	0	HR	0	0	HR	0	HR	155,56	0,2	HR	1	3
PI 269245	I 842	Portugal	Breeding material	120	120	0	HR	0	0	HR	1	HR	140,74	0,18	HR	1	4
PI 56239	Lobeiro	Portugal	Landrace	120	115	0	HR	0	0	HR	2	R	118,52	0,15	HR	1	4
PI 185737	Lobeiro	Portugal	Landrace	120	160	0	HR	0	0	HR	0	HR	188,89	0,24	R	1	3
PI 185741	Lobeiro de Grao Escuro	Portugal	Landrace	120	115	0	HR	0	0	HR	0	HR	140,74	0,18	HR	1	4
PI 192838	Lobeiro de	Portugal	Landrace	120	125	0	HR	0	0	HR	1	HR	177,78	0,23	R	1	4

	Grao Escuro																
PI 204036	Lobeiro Ruivo	Portugal	Landrace	120	125	0	HR	0	0	HR	1	HR	111,11	0,14	HR	1	3
PI 56247	Marquez	Portugal	Landrace	120	140	0	HR	0	0	HR	0	HR	118,52	0,15	HR	1	3
PI 191737	Marroquino Preto	Portugal	Landrace	120	125	0	HR	0	0	HR	0	HR	103,7	0,13	HR	1	3
PI 134916	2927	Portugal	Landrace	110	135	3	MR	587,65	0,38	R	1	HR	119,75	0,15	HR	2	1
PI 56248	Monjil	Portugal	Landrace	120	125	0	HR	0	0	HR	0	HR	118,52	0,15	HR	1	3
PI 56249	Mourisco	Portugal	Landrace	120	130	2	R	401,85	0,26	R	0	HR	103,7	0,13	HR	1	1
PI 192846	Mourisco Fino	Portugal	Landrace	120	135	0	HR	0	0	HR	1	HR	140,74	0,18	HR	1	3
PI 191971	Pardaleiro	Portugal	Unknown improvement status	120	120	0	HR	0	0	HR	0	HR	101,23	0,13	HR	1	4
PI 185410	Perdaleiro	Portugal	Unknown improvement status	120	130	1	HR	69,14	0,04	HR	1	HR	103,7	0,13	HR	1	3
PI 185731	Pombinho	Portugal	Landrace	120	165	0	HR	0	0	HR	0	HR	233,33	0,3	R	1	3
PI 56251	Raspinegro	Portugal	Landrace	120	135	1	HR	69,14	0,04	HR	0	HR	103,7	0,13	HR	1	3
PI 56240	Santa Martha	Portugal	Landrace	120	130	3	MR	246,3	0,16	HR	2	R	118,52	0,15	HR	1	4
PI 204018	Sicilio	Portugal	Landrace	120	150	0	HR	0	0	HR	1	HR	103,7	0,13	HR	1	3
PI 192840	Tremes Rijo	Portugal	Landrace	120	125	3	MR	345,68	0,22	R	0	HR	120,99	0,15	HR	1	4
PI 185742	Tremez Rijo	Portugal	Landrace	120	130	0	HR	0	0	HR	1	HR	103,7	0,13	HR	1	3
PI 190952	2161A	Portugal	Unknown improvement status	160	150	3	MR	298,15	0,19	HR	0	HR	128,4	0,16	HR	2	1
PI 190989	Alexandre	Portugal	Landrace	140	140	3	MR	622,22	0,4	MR	1	HR	140,74	0,18	HR	2	-
PI 204049	Alexandre	Portugal	Landrace	130	130	3	MR	466,67	0,3	R	1	HR	159,26	0,2	R	2	1
PI 270132	Amarelejo	Portugal	Cultivar	100	125	4	MS	764,81	0,5	MR	4	MS	155,56	0,2	HR	2	-
PI 192834	Amarelo de Barba Branca	Portugal	Landrace	110	140	3	MR	449,38	0,29	R	0	HR	140,74	0,18	HR	2	1
PI 191743	Anafil Claro	Portugal	Landrace	100	135	4	MS	449,38	0,29	R	1	HR	214,81	0,27	R	2	4
PI 192057	Anafil Claro	Portugal	Landrace	140	150	4	MS	669,75	0,44	MR	0	HR	103,7	0,13	HR	2	-
PI 192607	Aza de Corvo	Portugal	Unknown improvement status	120	115	3	MR	604,94	0,39	R	1	HR	140,74	0,18	HR	2	1

PI 192351	Amarelo de Barba Branca	Portugal	Landrace	125	135	0	HR	0	0	HR	0	HR	140,74	0,18	HR	1	3
PI 191732	Anafil Claro	Portugal	Landrace	125	130	0	HR	0	0	HR	2	R	103,7	0,13	HR	1	3
PI 204048	Anafil Claro	Portugal	Landrace	125	115	0	HR	0	0	HR	1	HR	175,31	0,22	R	1	4
PI 204032	Durazio Rijo Glabro	Portugal	Landrace	125	115	2	R	350	0,23	R	1	HR	103,7	0,13	HR	1	4
PI 192610	Entrelargo de Montijo	Portugal	Landrace	125	130	0	HR	0	0	HR	0	HR	103,7	0,13	HR	1	3
PI 192847	Entrelargo de Montijo	Portugal	Landrace	125	145	3	MR	328,4	0,21	R	1	HR	140,74	0,18	HR	1	1
PI 192857	Espanhol Mocho	Portugal	Landrace	125	135	0	HR	0	0	HR	0	HR	83,95	0,11	HR	1	3
PI 191879	H 22 A 13374	Portugal	Unknown improvement status	125	150	1	HR	69,14	0,04	HR	0	HR	103,7	0,13	HR	1	3
PI 191895	H N B 12442	Portugal	Unknown improvement status	125	150	0	HR	0	0	HR	0	HR	103,7	0,13	HR	1	3
PI 191880	H N H 12454	Portugal	Unknown improvement status	125	140	0	HR	0	0	HR	1	HR	103,7	0,13	HR	1	3
PI 191820	H N ROD 15 13760	Portugal	Unknown improvement status	125	150	2	R	246,3	0,16	HR	1	HR	103,7	0,13	HR	1	3
PI 191765	HN ROD 34 137781	Portugal	Unknown improvement status	125	145	0	HR	0	0	HR	0	HR	140,74	0,18	HR	1	3
PI 191787	HN ROD 41 14864	Portugal	Unknown improvement status	125	135	0	HR	0	0	HR	1	HR	103,7	0,13	HR	1	3
PI 185409	Lobeiro	Portugal	Landrace	125	120	0	HR	0	0	HR	NA	NA	214,81	0,27	R	1	4
PI 192828	Lobeiro	Portugal	Landrace	125	135	0	HR	0	0	HR	1	HR	159,26	0,2	R	1	3
PI 204045	Mourisco Preto de Grao Escuro	Portugal	Landrace	125	130	0	HR	0	0	HR	1	HR	140,74	0,18	HR	1	3
PI 192007	Verdeal Rijo	Portugal	Landrace	125	125	0	HR	0	0	HR	1	HR	190,12	0,24	R	1	3
PI 192067	Vermelejoilo	Portugal	Landrace	125	135	0	HR	0	0	HR	0	HR	140,74	0,18	HR	1	3
PI 192207	Canoco de Grao Escuro	Portugal	Landrace	140	150	4	MS	518,52	0,34	R	1	HR	64,2	0,08	HR	2	1

PI 185701	Fronteirico	Portugal	Landrace	120	115	4	MS	328,4	0,21	R	0	HR	218,52	0,28	R	2	4
PI 134921	2869	Portugal	Landrace	130	165	0	HR	0	0	HR	1	HR	140,74	0,18	HR	1	3
PI 134912	2883	Portugal	Landrace	130	145	0	HR	0	0	HR	1	HR	96,3	0,12	HR	1	3
PI 134913	2884	Portugal	Landrace	130	130	0	HR	0	0	HR	2	R	251,85	0,32	R	1	3
PI 134939	2886	Portugal	Landrace	130	145	0	HR	0	0	HR	0	HR	140,74	0,18	HR	1	3
PI 191626	H 22 C 13378	Portugal	Unknown improvement status	100	120	3	MR	561,73	0,37	R	0	HR	190,12	0,24	R	2	4
PI 134932	2909	Portugal	Landrace	130	125	0	HR	0	0	HR	1	HR	119,75	0,15	HR	1	3
PI 134915	2924	Portugal	Landrace	130	155	0	HR	0	0	HR	1	HR	140,74	0,18	HR	1	3
PI 134927	2929	Portugal	Landrace	130	125	0	HR	0	0	HR	0	HR	229,63	0,29	R	1	3
PI 134928	2930	Portugal	Landrace	130	135	0	HR	0	0	HR	1	HR	170,37	0,22	R	1	3
PI 191848	3233	Portugal	Breeding material	130	130	0	HR	0	0	HR	0	HR	103,7	0,13	HR	1	3
PI 192159	3589	Portugal	Breeding material	130	130	0	HR	0	0	HR	0	HR	103,7	0,13	HR	1	3
PI 204066	A Amarelejo	Portugal	Landrace	130	125	0	HR	0	0	HR	1	HR	103,7	0,13	HR	1	3
PI 185729	Alentejo	Portugal	Landrace	130	155	0	HR	0	0	HR	1	HR	140,74	0,18	HR	1	3
PI 204017	Alentejo	Portugal	Landrace	130	125	0	HR	0	0	HR	1	HR	148,15	0,19	HR	1	3
PI 56242	Amarello de Barba Preta	Portugal	Landrace	130	125	0	HR	0	0	HR	2	R	118,52	0,15	HR	1	3
PI 191979	Amarelo de Barba Preta	Portugal	Landrace	130	140	0	HR	0	0	HR	0	HR	140,74	0,18	HR	1	3
PI 204047	Amarelo de Barba Preta	Portugal	Landrace	130	140	0	HR	0	0	HR	1	HR	103,7	0,13	HR	1	3
PI 191883	H N K 12451	Portugal	Unknown improvement status	130	130	3	MR	466,67	0,3	R	0	HR	64,2	0,08	HR	2	1
PI 190996	Anafil Escuro	Portugal	Landrace	130	140	0	HR	0	0	HR	1	HR	214,81	0,27	R	1	3
PI 192825	Aza de Corvo	Portugal	Unknown improvement status	130	155	0	HR	0	0	HR	0	HR	64,2	0,08	HR	1	3
PI 192046	Barba de Lobo	Portugal	Landrace	130	115	0	HR	0	0	HR	1	HR	140,74	0,18	HR	1	3
PI 184541	H.M. 2	Portugal	Landrace	150	125	5	S	764,81	0,5	MR	3	MR	327,16	0,42	MR	2	-
PI 192826	Barba de Lobo	Portugal	Landrace	130	140	0	HR	0	0	HR	1	HR	103,7	0,13	HR	1	3

PI 192758	Belia	Portugal	Unknown improvement status	130	145	0	HR	0	0	HR	0	HR	64,2	0,08	HR	1	3
PI 192814	Branco	Portugal	Landrace	130	155	0	HR	0	0	HR	0	HR	103,7	0,13	HR	1	3
PI 192854	Durazio Molar	Portugal	Landrace	130	120	3	MR	328,4	0,21	R	1	HR	103,7	0,13	HR	1	1
PI 192005	Durazio Reichembachu	Portugal	Landrace	130	135	0	HR	0	0	HR	0	HR	170,37	0,22	R	1	3
PI 191741	Durazio Rijo	Portugal	Landrace	130	140	0	HR	0	0	HR	0	HR	103,7	0,13	HR	1	3
PI 185766	Espanhol	Portugal	Landrace	130	120	2	R	69,14	0,04	HR	0	HR	103,7	0,13	HR	1	3
PI 191735	Espanhol	Portugal	Landrace	130	130	2	R	298,15	0,19	HR	0	HR	103,7	0,13	HR	1	3
PI 270136	Guadiana	Portugal	Cultivar	130	115	0	HR	0	0	HR	0	HR	64,2	0,08	HR	1	3
PI 191805	H 29 D 13387	Portugal	Unknown improvement status	130	150	0	HR	0	0	HR	0	HR	190,12	0,24	R	1	3
PI 191822	H 29 D 13388	Portugal	Unknown improvement status	130	135	0	HR	0	0	HR	0	HR	103,7	0,13	HR	1	3
PI 191794	H 29 D 13795	Portugal	Unknown improvement status	130	140	0	HR	0	0	HR	0	HR	64,2	0,08	HR	1	3
PI 191623	HN ROD 30 13775	Portugal	Unknown improvement status	115	135	4	MS	514,2	0,33	R	1	HR	159,26	0,2	R	2	1
PI 191773	HN ROD 32 13777	Portugal	Unknown improvement status	90	85	5	S	1378,4	0,9	S	0	HR	333,33	0,42	MR	3	-
PI 191878	H N J 12449	Portugal	Unknown improvement status	130	135	0	HR	0	0	HR	0	HR	103,7	0,13	HR	1	3
PI 191855	HN ROD 17 13762	Portugal	Unknown improvement status	130	145	0	HR	0	0	HR	0	HR	103,7	0,13	HR	1	3
PI 191756	HN ROD 2013765	Portugal	Unknown improvement status	130	140	0	HR	0	0	HR	0	HR	200	0,25	R	1	3
PI 191817	HPxC35-13460	Portugal	Breeding material	130	155	0	HR	0	0	HR	1	HR	155,56	0,2	HR	1	3
PI 191627	HN ROD 42 14865	Portugal	Unknown improvement status	105	115	4	MS	466,67	0,3	R	0	HR	197,53	0,25	R	2	4

PI 204057	Javardo	Portugal	Landrace	130	130	3	MR	449,38	0,29	R	1	HR	64,2	0,08	HR	2	1
PI 204058	Javardo Glauco	Portugal	Landrace	130	150	0	HR	0	0	HR	1	HR	64,2	0,08	HR	1	3
PI 192760	Mocho de Espiga Branca	Portugal	Landrace	130	130	0	HR	0	0	HR	0	HR	103,7	0,13	HR	1	3
PI 191789	HN ROD 47 14870	Portugal	Unknown improvement status	110	NA	0	HR	0	0	HR	NA	NA	NA	NA	NA	-	-
PI 192180	Mourisco	Portugal	Landrace	130	140	0	HR	0	0	HR	1	HR	103,7	0,13	HR	1	3
PI 192845	Mourisco	Portugal	Landrace	130	165	0	HR	0	0	HR	1	HR	140,74	0,18	HR	1	3
PI 192437	Mourisco Preto de Grao Escuro	Portugal	Landrace	130	145	0	HR	0	0	HR	0	HR	103,7	0,13	HR	1	3
PI 134958	Nigrobarbatum	Portugal	Landrace	130	150	0	HR	0	0	HR	0	HR	64,2	0,08	HR	1	3
PI 204034	Pragana Preta	Portugal	Landrace	130	110	3	MR	350	0,23	R	1	HR	118,52	0,15	HR	1	4
PI 184527	Raspinegro	Portugal	Landrace	130	115	0	HR	0	0	HR	0	HR	140,74	0,18	HR	1	3
PI 191728	Raspinegro	Portugal	Landrace	130	130	3	MR	298,15	0,19	HR	1	HR	103,7	0,13	HR	1	3
PI 269241	I 838	Portugal	Breeding material	130	120	2	R	678,4	0,44	MR	5	S	NA	NA	NA	-	-
PI 204052	Raspinegro	Portugal	Landrace	130	125	0	HR	0	0	HR	1	HR	140,74	0,18	HR	1	3
PI 192161	Rijo	Portugal	Landrace	130	160	2	R	246,3	0,16	HR	0	HR	103,7	0,13	HR	1	3
PI 185727	Rubiao	Portugal	Landrace	130	160	0	HR	0	0	HR	1	HR	103,7	0,13	HR	1	3
PI 192818	Rubiao	Portugal	Landrace	130	130	0	HR	0	0	HR	1	HR	64,2	0,08	HR	1	3
PI 204035	Santa Marta	Portugal	Landrace	130	125	2	R	229,01	0,15	HR	1	HR	118,52	0,15	HR	1	3
PI 191640	Javardo Glauco	Portugal	Landrace	140	150	1	HR	453,7	0,3	R	1	HR	103,7	0,13	HR	2	1
PI 192851	Tremes Preto	Portugal	Landrace	130	115	0	HR	0	0	HR	1	HR	120,99	0,15	HR	1	3
PI 191742	Javardo Glouco	Portugal	Landrace	125	135	4	MS	449,38	0,29	R	2	R	198,77	0,25	R	2	1
PI 192839	Tremes Rijo	Portugal	Landrace	130	125	3	MR	328,4	0,21	R	1	HR	140,74	0,18	HR	1	1
PI 191227	Verdeal Rijo	Portugal	Landrace	130	145	0	HR	0	0	HR	2	R	118,52	0,15	HR	1	3
PI 56241	Vermelejoilo	Portugal	Landrace	130	140	0	HR	0	0	HR	2	R	103,7	0,13	HR	1	3
PI 192149	Vermelejoilo	Portugal	Landrace	130	130	0	HR	0	0	HR	1	HR	103,7	0,13	HR	1	3
PI 204030	Lobeiro de	Portugal	Landrace	120	105	4	MS	596,3	0,39	R	1	HR	103,7	0,13	HR	2	4

	Grao Escuro																
PI 192008	Pragana Preta	Portugal	Landrace	100	110	2	R	535,8	0,35	R	0	HR	140,74	0,18	HR	2	4
PI 204043	Preto Algarvio	Portugal	Landrace	120	140	4	MS	466,67	0,3	R	1	HR	118,52	0,15	HR	2	1
PI 203983	Preto Amarelo	Portugal	Landrace	130	160	4	MS	583,33	0,38	R	1	HR	118,52	0,15	HR	2	1
PI 192116	3531	Portugal	Unknown improvement status	135	135	0	HR	0	0	HR	0	HR	103,7	0,13	HR	1	3
PI 192819	Alentejo	Portugal	Landrace	135	120	0	HR	0	0	HR	1	HR	64,2	0,08	HR	1	3
PI 185763	Anafil Escuro	Portugal	Landrace	135	135	0	HR	0	0	HR	0	HR	200	0,25	R	1	3
PI 204021	Aza de Corvo	Portugal	Landrace	135	150	0	HR	0	0	HR	0	HR	155,56	0,2	HR	1	3
PI 191697	Raspinegro	Portugal	Landrace	150	125	4	MS	328,4	0,21	R	0	HR	198,77	0,25	R	2	1
PI 192823	Bagudo	Portugal	Landrace	135	145	0	HR	0	0	HR	0	HR	120,99	0,15	HR	1	3
PI 185721	Branco	Portugal	Landrace	135	145	0	HR	0	0	HR	2	R	140,74	0,18	HR	1	3
PI 185745	Durazio Molar Glabro	Portugal	Landrace	135	110	3	MR	69,14	0,04	HR	1	HR	103,7	0,13	HR	1	3
PI 191849	H N J 12450	Portugal	Unknown improvement status	135	130	0	HR	0	0	HR	0	HR	155,56	0,2	HR	1	3
PI 191770	HN ROD 44 14867	Portugal	Unknown improvement status	135	145	0	HR	0	0	HR	2	R	103,7	0,13	HR	1	3
PI 185770	Javardo Glauco	Portugal	Landrace	135	135	0	HR	0	0	HR	0	HR	140,74	0,18	HR	1	3
PI 192817	Rubiao	Portugal	Landrace	140	NA	0	HR	0	0	HR	NA	NA	39,51	0,05	HR	-	NA
PI 204054	Marroquino Preto	Portugal	Landrace	135	125	0	HR	0	0	HR	2	R	64,2	0,08	HR	1	3
PI 204039	Mongia	Portugal	Landrace	135	135	1	HR	207,41	0,13	HR	1	HR	148,15	0,19	HR	1	3
PI 191941	Rubiao de Barba Preta	Portugal	Landrace	125	115	5	S	933,33	0,61	MS	2	R	318,52	0,41	MR	2	-
PI 192815	nacional	Portugal	Unknown improvement status	135	170	0	HR	0	0	HR	1	HR	103,7	0,13	HR	1	3
PI 192438	Preto de Tavira	Portugal	Landrace	135	155	0	HR	0	0	HR	2	R	103,7	0,13	HR	1	3
PI 191926	Raspinegro	Portugal	Landrace	135	125	4	MS	246,3	0,16	HR	1	HR	155,56	0,2	HR	1	1
PI 192842	Santa Marta	Portugal	Landrace	135	125	3	MR	328,4	0,21	R	1	HR	159,26	0,2	R	2	1

PI 192843	Santa Marta	Portugal	Landrace	135	140	0	HR	0	0	HR	1	HR	140,74	0,18	HR	1	3
PI 185187	Vermeleijoilo	Portugal	Landrace	135	145	1	HR	69,14	0,04	HR	0	HR	140,74	0,18	HR	1	3
PI 192609	Vermelejoilo	Portugal	Landrace	135	145	0	HR	0	0	HR	1	HR	64,2	0,08	HR	1	3
PI 204020	Vermelho de Barba Preta	Portugal	Landrace	135	145	1	HR	69,14	0,04	HR	1	HR	64,2	0,08	HR	1	3
PI 192844	Vermeljoilo	Portugal	Landrace	135	145	1	HR	69,14	0,04	HR	1	HR	64,2	0,08	HR	1	3
PI 56258	Tremez Molle	Portugal	Landrace	110	115	3	MR	535,8	0,35	R	3	MR	111,11	0,14	HR	2	4
PI 56257	Tremez Rijo	Portugal	Landrace	115	125	3	MR	466,67	0,3	R	1	HR	151,85	0,19	HR	2	1
PI 192738	Vermelho Fino	Portugal	Landrace	115	125	4	MS	1058,64	0,69	MS	1	HR	88,89	0,11	HR	2	-
PI 134936	2875	Portugal	Landrace	140	135	1	HR	69,14	0,04	HR	0	HR	88,89	0,11	HR	1	3
PI 134918	2882	Portugal	Landrace	140	145	1	HR	69,14	0,04	HR	1	HR	159,26	0,2	R	1	3
PI 134941	2888	Portugal	Landrace	140	145	0	HR	0	0	HR	0	HR	101,23	0,13	HR	1	3
PI 134938	2893	Portugal	Landrace	140	145	0	HR	0	0	HR	0	HR	190,12	0,24	R	1	3
PI 134926	2928	Portugal	Landrace	140	155	0	HR	0	0	HR	0	HR	170,37	0,22	R	1	3
PI 191833	3217	Portugal	Breeding material	140	150	0	HR	0	0	HR	0	HR	135,8	0,17	HR	1	3
PI 192148	3570	Portugal	Breeding material	140	155	3	MR	229,01	0,15	HR	1	HR	103,7	0,13	HR	1	3
PI 192458	3981	Portugal	Breeding material	140	145	3	MR	328,4	0,21	R	1	HR	64,2	0,08	HR	1	1
PI 192832	Amarelo de Barba Branca	Portugal	Landrace	140	155	0	HR	0	0	HR	1	HR	155,56	0,2	HR	1	3
PI 192833	Amarelo de Barba Branca	Portugal	Landrace	140	140	0	HR	0	0	HR	1	HR	177,78	0,23	R	1	3
PI 204013	Argelino	Portugal	Landrace	140	155	0	HR	0	0	HR	1	HR	64,2	0,08	HR	1	3
PI 192822	Bagudo	Portugal	Landrace	140	140	0	HR	0	0	HR	0	HR	190,12	0,24	R	1	3
PI 191982	Branco	Portugal	Landrace	140	140	0	HR	0	0	HR	1	HR	103,7	0,13	HR	1	3
PI 192061	Candeal	Portugal	Landrace	140	130	0	HR	0	0	HR	0	HR	103,7	0,13	HR	1	3
PI 204012	Canoco	Portugal	Landrace	140	140	0	HR	0	0	HR	1	HR	64,2	0,08	HR	1	3
PI 192816	Cascalvo	Portugal	Landrace	140	160	0	HR	0	0	HR	1	HR	64,2	0,08	HR	1	3
PI 192827	Dezassete	Portugal	Landrace	140	135	0	HR	0	0	HR	1	HR	101,23	0,13	HR	1	3
PI 191714	Durazio Molar	Portugal	Landrace	140	150	0	HR	0	0	HR	0	HR	103,7	0,13	HR	1	3
PI 204051	Durazio Molar	Portugal	Landrace	140	120	0	HR	0	0	HR	1	HR	159,26	0,2	R	1	3

PI 185761	Durazio Rijo	Portugal	Landrace	140	135	3	MR	298,15	0,19	HR	2	R	103,7	0,13	HR	1	1
PI 191711	Durazio Rijo	Portugal	Landrace	140	150	0	HR	0	0	HR	0	HR	103,7	0,13	HR	1	3
PI 185772	Gigantil	Portugal	Landrace	140	130	1	HR	298,15	0,19	HR	0	HR	103,7	0,13	HR	1	1
PI 191760	H M 4 12697	Portugal	Unknown improvement status	140	145	0	HR	0	0	HR	0	HR	140,74	0,18	HR	1	3
PI 191633	HN ROD 52 14875	Portugal	Unknown improvement status	140	145	0	HR	0	0	HR	1	HR	140,74	0,18	HR	1	3
PI 191632	HND 12444	Portugal	Unknown improvement status	140	150	0	HR	0	0	HR	1	HR	190,12	0,24	R	1	3
PI 191795	HNL 12452	Portugal	Unknown improvement status	140	170	0	HR	0	0	HR	1	HR	103,7	0,13	HR	1	3
PI 191762	HP C C 41 13464	Portugal	Unknown improvement status	140	145	1	HR	69,14	0,04	HR	2	R	160,49	0,2	R	1	3
PI 266904	Lobeiro	Portugal	Landrace	140	130	0	HR	0	0	HR	0	HR	214,81	0,27	R	1	3
PI 204041	Mourisco Fino	Portugal	Landrace	140	135	0	HR	0	0	HR	1	HR	103,7	0,13	HR	1	3
PI 204042	Mourisco Preto	Portugal	Landrace	140	140	0	HR	0	0	HR	1	HR	103,7	0,13	HR	1	3
PI 184645	Novo	Portugal	Landrace	140	160	0	HR	0	0	HR	0	HR	88,89	0,11	HR	1	3
PI 192821	Pombinho	Portugal	Landrace	140	135	0	HR	0	0	HR	1	HR	83,95	0,11	HR	1	3
PI 204019	Pombinho	Portugal	Landrace	140	155	0	HR	0	0	HR	1	HR	103,7	0,13	HR	1	3
PI 185746	Pragana Preta	Portugal	Landrace	140	125	0	HR	0	0	HR	2	R	279,01	0,36	R	1	3
PI 192136	Pragana Preta	Portugal	Landrace	140	130	0	HR	0	0	HR	0	HR	103,7	0,13	HR	1	3
PI 185744	Preto Amarelo	Portugal	Landrace	140	140	3	MR	246,3	0,16	HR	0	HR	140,74	0,18	HR	1	3
PI 192850	Preto de Tavira	Portugal	Landrace	140	130	2	R	246,3	0,16	HR	0	HR	64,2	0,08	HR	1	3
PI 204016	Rubiao de Barba Preta	Portugal	Landrace	140	160	0	HR	0	0	HR	1	HR	83,95	0,11	HR	1	3
PI 192829	Russo	Portugal	Landrace	140	125	0	HR	0	0	HR	2	R	120,99	0,15	HR	1	3
PI 192831	Russo	Portugal	Landrace	140	125	0	HR	0	0	HR	0	HR	111,11	0,14	HR	1	3
PI 204027	Russo	Portugal	Landrace	140	130	0	HR	0	0	HR	1	HR	111,11	0,14	HR	1	3

PI 185747	Santa Marta	Portugal	Landrace	140	135	2	R	69,14	0,04	HR	2	R	103,7	0,13	HR	1	3
PI 185730	Sicilio	Portugal	Landrace	140	155	0	HR	0	0	HR	0	HR	140,74	0,18	HR	1	3
PI 185758	Tremez Preto	Portugal	Landrace	140	135	0	HR	0	0	HR	0	HR	103,7	0,13	HR	1	3
PI 204044	Verdeal Rijo	Portugal	Landrace	140	135	0	HR	0	0	HR	0	HR	249,38	0,32	R	1	3
PI 185749	Vermelejoilo	Portugal	Landrace	140	150	0	HR	0	0	HR	2	R	103,7	0,13	HR	1	3
PI 56253	Vermelho Fino	Portugal	Landrace	140	145	0	HR	0	0	HR	2	R	103,7	0,13	HR	1	3
PI 204037	Vermelho Fino	Portugal	Landrace	140	145	0	HR	0	0	HR	2	R	111,11	0,14	HR	1	3
PI 185759	Amarelo de Barba Preta	Portugal	Landrace	145	130	0	HR	0	0	HR	0	HR	103,7	0,13	HR	1	3
PI 204014	Canoco de Grao Escuro	Portugal	Landrace	145	160	0	HR	0	0	HR	1	HR	83,95	0,11	HR	1	3
PI 185764	Durazio Molar	Portugal	Landrace	145	130	3	MR	298,15	0,19	HR	2	R	103,7	0,13	HR	1	1
PI 191832	HN ROD 39 13786	Portugal	Unknown improvement status	145	145	0	HR	0	0	HR	1	HR	103,7	0,13	HR	1	3
PI 192625	2241A	Portugal	Breeding material	150	150	2	R	272,22	0,18	HR	1	HR	140,74	0,18	HR	2	1
PI 192734	Alexandre	Portugal	Landrace	150	140	0	HR	0	0	HR	0	HR	103,7	0,13	HR	1	3
PI 204028	Amarelo de Barba Branca	Portugal	Landrace	150	150	0	HR	0	0	HR	1	HR	103,7	0,13	HR	1	3
PI 192611	Anafil Escuro	Portugal	Landrace	150	140	0	HR	0	0	HR	0	HR	103,7	0,13	HR	1	3
PI 204050	Anafil Escuro	Portugal	Landrace	150	115	0	HR	0	0	HR	1	HR	101,23	0,13	HR	1	3
PI 191540	Anca	Portugal	Unknown improvement status	150	145	0	HR	0	0	HR	1	HR	[illegible],74	0,18	HR	1	3
PI 191986	Durazio Rijo Glabro	Portugal	Landrace	150	135	0	HR	0	0	HR	0	HR	200	0,25	R	1	3
PI 204056	Escuro	Portugal	Landrace	150	120	0	HR	0	0	HR	2	R	64,2	0,08	HR	1	3
PI 191841	H 22 E 13380	Portugal	Unknown improvement status	150	155	0	HR	0	0	HR	1	HR	103,7	0,13	HR	1	3
PI 191768	H N ROD 36 13783	Portugal	Unknown improvement status	150	140	0	HR	0	0	HR	0	HR	140,74	0,18	HR	1	3
PI 192608	Lobeiro de Grao Escuro	Portugal	Landrace	150	150	0	HR	0	0	HR	1	HR	103,7	0,13	HR	1	3

PI 204040	Mongia de Grao Escuro	Portugal	Landrace	150	130	0	HR	0	0	HR	1	HR	101,23	0,13	HR	1	3
PI 185757	Mourisco Preto de Grao Escuro	Portugal	Landrace	150	120	0	HR	0	0	HR	1	HR	140,74	0,18	HR	1	3
PI 204046	Mourisco Ruivo	Portugal	Landrace	150	155	0	HR	0	0	HR	1	HR	103,7	0,13	HR	1	3
PI 204011	Novo	Portugal	Landrace	150	145	2	R	246,3	0,16	HR	1	HR	118,52	0,15	HR	1	3
PI 184542	Preto Amarelo	Portugal	Landrace	150	140	0	HR	0	0	HR	0	HR	140,74	0,18	HR	1	3
PI 192087	Rubiao	Portugal	Landrace	150	160	0	HR	0	0	HR	0	HR	64,2	0,08	HR	1	3
PI 185738	Russo	Portugal	Landrace	150	170	0	HR	0	0	HR	0	HR	103,7	0,13	HR	1	3
PI 204029	Candeal de Grao Escuro	Portugal	Landrace	155	160	0	HR	0	0	HR	1	HR	103,7	0,13	HR	1	3
PI 191896	Jerez	Portugal	Landrace	155	145	0	HR	0	0	HR	0	HR	103,7	0,13	HR	1	3
PI 185740	Candeal de Grao Escuro	Portugal	Landrace	160	155	0	HR	0	0	HR	0	HR	103,7	0,13	HR	1	3
PI 185768	Raposinho	Portugal	Landrace	160	135	0	HR	0	0	HR	0	HR	200	0,25	R	1	3
PI 204055	Raposinho	Portugal	Landrace	160	120	0	HR	0	0	HR	0	HR	101,23	0,13	HR	1	3
PI 191181	Raspinegro de Aguilas	Spain	Landrace	100	125	0	HR	0	0	HR	0	HR	101,23	0,13	HR	1	4
PI 221406	Raspinegro	Spain	Landrace	105	150	3	MR	207,41	0,13	HR	4	MS	222,22	0,28	R	1	4
PI 191190	Recio de Granada	Spain	Landrace	105	140	0	HR	0	0	HR	1	HR	101,23	0,13	HR	1	3
PI 191249	Trigo de Albandea	Spain	Landrace	110	100	0	HR	0	0	HR	3	MR	132,1	0,17	HR	1	4
PI 308878		Spain	Breeding material	115	125	2	R	246,3	0,16	HR	3	MR	274,07	0,35	R	1	4
PI 191009	Arlante	Spain	Landrace	120	145	0	HR	0	0	HR	0	HR	177,78	0,23	R	1	3
PI 191019	Berberisco	Spain	Landrace	120	150	0	HR	0	0	HR	2	R	64,2	0,08	HR	1	3
PI 191089	Duro de Cadreita	Spain	Landrace	120	130	0	HR	0	0	HR	2	R	140,74	0,18	HR	1	3
PI 191908	Fanfarron	Spain	Landrace	120	160	3	MR	229,01	0,15	HR	1	HR	118,52	0,15	HR	1	3
PI 191103	Farto Rubio	Spain	Landrace	120	140	0	HR	0	0	HR	0	HR	159,26	0,2	R	1	3
PI 191127	Jerez 1937	Spain	Cultivar	120	140	1	HR	69,14	0,04	HR	2	R	140,74	0,18	HR	1	3
PI 191179	Oned Zenati	Spain	Landrace	120	145	0	HR	0	0	HR	2	R	101,23	0,13	HR	1	3

PI 191182	Raspinegro de Alcala	Spain	Landrace	120	145	0	HR	0	0	HR	1	HR	148,15	0,19	HR	1	3
PI 191193	Rojal de Alicante	Spain	Landrace	120	125	1	HR	69,14	0,04	HR	2	R	83,95	0,11	HR	1	3
PI 191194	Rojal de Almeria	Spain	Landrace	120	115	3	MR	298,15	0,19	HR	2	R	135,8	0,17	HR	1	4
PI 191208	Ruso	Spain	Landrace	120	145	0	HR	0	0	HR	2	R	140,74	0,18	HR	1	3
PI 136575	Semental de Sevilla	Spain	Landrace	120	125	0	HR	0	0	HR	0	HR	101,23	0,13	HR	1	3
PI 191225	Valenciano	Spain	Landrace	120	145	0	HR	0	0	HR	3	MR	103,7	0,13	HR	1	3
PI 190995	Amoros	Spain	Landrace	130	165	0	HR	0	0	HR	0	HR	103,7	0,13	HR	1	3
PI 190997	Andalucia 344	Spain	Cultivar	130	150	0	HR	0	0	HR	0	HR	140,74	0,18	HR	1	3
PI 191010	Azul de Carmona	Spain	Landrace	130	145	0	HR	0	0	HR	2	R	144,44	0,18	HR	1	3
PI 191012	Azulejo de Villa del Rio	Spain	Landrace	130	160	0	HR	0	0	HR	2	R	103,7	0,13	HR	1	3
PI 191088	Duro Africano	Spain	Landrace	130	160	0	HR	0	0	HR	1	HR	64,2	0,08	HR	1	3
PI 191163	navarro 172	Spain	Breeding material	130	140	0	HR	0	0	HR	1	HR	203,7	0,26	R	1	3
PI 191330	Pinsana	Spain	Landrace	130	135	0	HR	0	0	HR	2	R	103,7	0,13	HR	1	3
PI 191959	Portugues I	Spain	Unknown improvement status	130	140	2	R	246,3	0,16	HR	0	HR	140,74	0,18	HR	1	3
PI 191188	Raspinegro de Villanueva	Spain	Landrace	130	150	2	R	246,3	0,16	HR	2	R	107,41	0,14	HR	1	3
PI 191399	Recio	Spain	Landrace	135	145	3	MR	328,4	0,21	R	2	R	103,7	0,13	HR	1	1
PI 191206	Rubio de Miajadas	Spain	Landrace	135	160	3	MR	298,15	0,19	HR	3	MR	162,96	0,21	R	2	1
PI 190987	Alcala la Real	Spain	Landrace	140	145	4	MS	583,33	0,38	R	2	R	103,7	0,13	HR	2	1
PI 352432	Andalucia 344	Spain	Cultivar	120	115	5	S	760,49	0,49	MR	0	HR	237,04	0,3	R	2	-
PI 191057	Cascalvo Lucena y Montilla	Spain	Landrace	150	145	5	S	574,69	0,37	R	2	R	139,51	0,18	HR	2	1
PI 191090	Enano de Jaen	Spain	Landrace	130	140	3	MR	643,83	0,42	MR	2	R	111,11	0,14	HR	2	-
PI 191464	Macolo de Jerez	Spain	Landrace	120	130	4	MS	639,51	0,42	MR	3	MR	107,41	0,14	HR	2	-
CItr 13918	Morisco Selection	Spain	Landrace	150	160	3	MR	535,8	0,35	R	0	HR	214,81	0,27	R	2	1

PI 191173	navarro 302	Spain	Breeding material	140	150	4	MS	669,75	0,44	MR	2	R	140,74	0,18	HR	2	-
PI 191186	Raspinegro de Huelva	Spain	Landrace	130	165	5	S	535,8	0,35	R	2	R	103,7	0,13	HR	2	1
PI 191189	Recio de Almeria	Spain	Landrace	140	140	3	MR	466,67	0,3	R	0	HR	111,11	0,14	HR	2	1
PI 191256	Recio Pais	Spain	Landrace	145	155	4	MS	535,8	0,35	R	3	MR	222,22	0,28	R	2	1
PI 191195	Rojal de Murcia	Spain	Landrace	150	145	4	MS	350	0,23	R	2	R	125,93	0,16	HR	2	1
PI 191205	Rubio de Belalcazar	Spain	Landrace	150	140	5	S	293,83	0,19	HR	2	R	175,31	0,22	R	2	1
PI 191214	Senatore Capelli	Spain	Landrace	115	130	3	MR	246,3	0,16	HR	2	R	313,58	0,4	R	2	4
PI 190983	Adjini	Spain	Landrace	140	160	1	HR	69,14	0,04	HR	0	HR	140,74	0,18	HR	1	3
PI 190991	Alonso	Spain	Landrace	140	130	0	HR	0	0	HR	1	HR	148,15	0,19	HR	1	3
PI 191076	Claro Fino de Balazote	Spain	Landrace	140	160	0	HR	0	0	HR	0	HR	101,23	0,13	HR	1	3
PI 191079	Colorado de Jerez	Spain	Landrace	140	150	0	HR	0	0	HR	1	HR	140,74	0,18	HR	1	3
PI 191259	Duro 0181	Spain	Breeding material	140	135	0	HR	0	0	HR	1	HR	175,31	0,22	R	1	3
PI 191258	Duro 1658	Spain	Breeding material	140	155	0	HR	0	0	HR	3	MR	103,7	0,13	HR	1	3
PI 191087	Duro Sin Nombre	Spain	Unknown improvement status	140	165	0	HR	0	0	HR	1	HR	101,23	0,13	HR	1	3
PI 191102	Farto Blanco	Spain	Landrace	140	145	3	MR	350	0,23	R	1	HR	120,99	0,15	HR	2	1
PI 191105	Fino o Claro de Petrola	Spain	Landrace	140	150	0	HR	0	0	HR	0	HR	148,15	0,19	HR	1	3
PI 191129	Langlois	Spain	Cultivar	140	120	0	HR	0	0	HR	2	R	148,15	0,19	HR	1	3
PI 191178	Obispado de Lebrija	Spain	Landrace	140	145	0	HR	0	0	HR	1	HR	83,95	0,11	HR	1	3
PI 191187	Raspinegro de Murcia	Spain	Landrace	140	145	0	HR	0	0	HR	2	R	214,81	0,27	R	1	3
PI 191192	Recio de Toledo	Spain	Landrace	140	150	0	HR	0	0	HR	1	HR	140,74	0,18	HR	1	3
PI 191239	Santa Coloma	Spain	Landrace	140	140	1	HR	69,14	0,04	HR	NA	NA	107,41	0,14	HR	1	3
PI 191235	Zenati bouteille	Spain	Unknown improvement status	140	150	0	HR	0	0	HR	0	HR	218,52	0,28	R	1	3

PI 191255	Duro 270	Spain	Breeding material	145	155	1	HR	69,14	0,04	HR	3	MR	118,52	0,15	HR	1	3
PI 191223	Valenciano	Spain	Landrace	145	145	0	HR	0	0	HR	0	HR	107,41	0,14	HR	1	3
PI 190938	3990	Spain	Unknown improvement status	150	150	2	R	69,14	0,04	HR	0	HR	143,21	0,18	HR	1	3
PI 191021	Blanco de Corella	Spain	Landrace	150	155	3	MR	337,04	0,22	R	1	HR	64,2	0,08	HR	2	1
PI 191254	Duro 250	Spain	Breeding material	150	165	1	HR	69,14	0,04	HR	2	R	174,07	0,22	R	1	3
PI 191449	Medeah	Spain	Landrace	150	135	0	HR	0	0	HR	2	R	103,7	0,13	HR	1	3
PI 191476	Recio de Malaga	Spain	Landrace	150	155	0	HR	0	0	HR	2	R	111,11	0,14	HR	1	3
PI 191251	Recio San Clemente	Spain	Landrace	150	140	0	HR	0	0	HR	2	R	118,52	0,15	HR	1	3
PI 191224	Valenciano	Spain	Landrace	150	150	0	HR	0	0	HR	2	R	230,86	0,29	R	1	3
PI 190992	Alonso Perez Jurado	Spain	Landrace	160	175	1	HR	69,14	0,04	HR	0	HR	285,19	0,36	R	2	3
PI 190936	3896	Spain	Unknown improvement status	170	150	2	R	69,14	0,04	HR	0	HR	128,4	0,16	HR	1	3
PI 193390		Syria	Landrace	115	160	0	HR	0	0	HR	2	R	344,44	0,44	MR	1	-
CItr 15909	3380-34	Tunisia	Landrace	90	145	3	MR	66,67	0,08	HR	5	S	345,68	0,22	R	1	4
CItr 15472	Derbazi	Tunisia	Landrace	90	105	0	HR	98,77	0,13	HR	0	HR	0	0	HR	1	4
CItr 15469	Farine	Tunisia	Landrace	90	135	0	HR	121,6	0,15	HR	1	HR	69,14	0,04	HR	1	4
CItr 15470	Mexiquain	Tunisia	Unknown improvement status	90	100	0	HR	16,05	0,02	HR	0	HR	0	0	HR	1	4
PI 306577	D 56-27-A	Tunisia	Breeding material	95	110	0	HR	48,15	0,06	HR	3	MR	483,95	0,31	R	1	4
CItr 15431	1346-27	Tunisia	Landrace	100	125	4	MS	0	0	HR	1	HR	69,14	0,04	HR	1	4
CItr 3171	Bidi AP 2	Tunisia	Landrace	100	160	0	HR	90,12	0,11	HR	0	HR	0	0	HR	1	3
CItr 3186	Ble a Epi Cane RC 1	Tunisia	Landrace	100	135	0	HR	0	0	HR	0	HR	0	0	HR	1	4
CItr 15442	Hmira	Tunisia	Landrace	100	85	4	MS	0	0	HR	3	MR	345,68	0,22	R	1	4
CItr 15443	Hmira	Tunisia	Landrace	100	125	NA	NA	49,38	0,06	HR	4	MS	483,95	0,31	R	1	4
PI 191646	Agili Blanc	Tunisia	Unknown	110	155	0	HR	146,91	0,19	HR	3	MR	337,04	0,22	R	1	1

			improvement status														
CItr 3147	Agili Pubescent AC 1	Tunisia	Landrace	110	140	0	HR	156,79	0,2	HR	0	HR	0	0	HR	1	3
PI 55531	Agiliblanc 1	Tunisia	Landrace	110	125	0	HR	48,15	0,06	HR	0	HR	0	0	HR	1	4
CItr 15419	Hmira	Tunisia	Landrace	110	130	3	MR	0	0	HR	3	MR	328,4	0,21	R	1	4
CItr 15421	Hmira	Tunisia	Landrace	110	115	4	MS	0	0	HR	4	MS	466,67	0,3	R	1	4
PI 150380	Mahmoudi	Tunisia	Landrace	110	115	0	HR	81,48	0,1	HR	0	HR	0	0	HR	1	4
PI 534354	MG 18142	Tunisia	Landrace	110	110	0	HR	301,23	0,38	R	0	HR	0	0	HR	1	4
CItr 3261	Real Sorte AC 3	Tunisia	Landrace	110	150	0	HR	0	0	HR	0	HR	246,3	0,16	HR	1	3
PI 55533	Aouedj	Tunisia	Landrace	115	150	0	HR	48,15	0,06	HR	0	HR	0	0	HR	1	3
PI 324931	BD 1342	Tunisia	Breeding material	115	125	0	HR	0	0	HR	1	HR	130,86	0,17	HR	1	4
CItr 3192	Derberri RC 1	Tunisia	Landrace	115	115	0	HR	146,91	0,19	HR	0	HR	298,15	0,19	HR	1	4
CItr 3237	Mahmoudi Glabre AP 3	Tunisia	Landrace	115	130	0	HR	48,15	0,06	HR	1	HR	190,12	0,12	HR	1	4
CItr 15505	2116-16	Tunisia	Landrace	120	100	3	MR	16,05	0,02	HR	1	HR	203,09	0,13	HR	1	4
CItr 15439	Djebali	Tunisia	Landrace	120	110	NA	NA	106,79	0,14	HR	4	MS	276,54	0,18	HR	1	4
CItr 15897	Frigui	Tunisia	Landrace	120	140	0	HR	138,27	0,18	HR	2	R	388,89	0,25	R	1	1
CItr 3210	Lebei Pubescent AP 2	Tunisia	Landrace	120	150	4	MS	213,58	0,27	R	2	R	203,09	0,13	HR	1	1
CItr 3242	Mahmoudi Pubescent AP 1	Tunisia	Landrace	120	140	2	R	64,81	0,08	HR	1	HR	69,14	0,04	HR	1	3
PI 185411	Mahon Rijo	Tunisia	Unknown improvement status	120	150	0	HR	81,48	0,1	HR	0	HR	0	0	HR	1	3
PI 41044	Mekki	Tunisia	Landrace	120	130	0	HR	148,15	0,19	HR	1	HR	241,98	0,16	HR	1	4
PI 185194	Mekki 13	Tunisia	Cultivar	120	135	NA	NA	146,91	0,19	HR	2	R	298,15	0,19	HR	1	1
PI 185195	Sbei 7	Tunisia	Cultivar	120	125	3	MR	48,15	0,06	HR	1	HR	69,14	0,04	HR	1	3
CItr 3262	Taganrog AC 1	Tunisia	Landrace	120	170	0	HR	58,02	0,07	HR	1	HR	69,14	0,04	HR	1	3
PI 55530	Agili	Tunisia	Landrace	125	145	0	HR	200	0,25	R	1	HR	69,14	0,04	HR	1	3

CItr 3155	Aouej AP 3	Tunisia	Landrace	125	145	0	HR	33,33	0,04	HR	2	R	350	0,23	R	1	3
CItr 3174	Bidi AP 5	Tunisia	Landrace	125	150	0	HR	81,48	0,1	HR	0	HR	0	0	HR	1	3
PI 352413	Mahmoudi	Tunisia	Landrace	125	150	0	HR	69,14	0,04	HR	2	R	162,96	0,21	R	1	3
CItr 3816	Mahmoudi	Tunisia	Landrace	125	135	0	HR	98,77	0,13	HR	0	HR	0	0	HR	1	3
PI 55539	Mahmoudi AP 5	Tunisia	Landrace	125	130	1	HR	114,81	0,15	HR	0	HR	0	0	HR	1	3
CItr 15410	3124-10	Tunisia	Landrace	130	135	3	MR	213,58	0,27	R	0	HR	203,09	0,13	HR	1	1
CItr 3140	Agili Glabre AP 1	Tunisia	Landrace	130	145	0	HR	66,67	0,08	HR	0	HR	0	0	HR	1	3
CItr 3163	Azizi AP 6	Tunisia	Landrace	130	145	0	HR	96,3	0,12	HR	0	HR	0	0	HR	1	3
CItr 3173	Bidi AP 4	Tunisia	Landrace	130	140	0	HR	48,15	0,06	HR	0	HR	0	0	HR	1	3
CItr 6872	Biskri	Tunisia	Landrace	130	145	NA	NA	16,05	0,02	HR	2	R	241,98	0,16	HR	1	3
CItr 3175	Biskri Glabre AC 2	Tunisia	Landrace	130	140	0	HR	165,43	0,21	R	1	HR	203,09	0,13	HR	1	3
CItr 3176	Biskri Glabre AP 1	Tunisia	Landrace	130	135	0	HR	0	0	HR	0	HR	0	0	HR	1	3
CItr 3179	Biskri Glabre RP 1	Tunisia	Landrace	130	145	0	HR	0	0	HR	1	HR	216,05	0,14	HR	1	3
CItr 3184	Biskri Pubescent AP 1	Tunisia	Landrace	130	135	0	HR	0	0	HR	0	HR	0	0	HR	1	3
CItr 3197	Hamira AC 5	Tunisia	Landrace	130	145	3	MR	81,48	0,1	HR	0	HR	0	0	HR	1	3
CItr 3203	Jennah Rhetifa RP 2	Tunisia	Landrace	130	155	0	HR	81,48	0,1	HR	2	R	350	0,23	R	1	3
CItr 3212	Lebei Velu Bas AC 2	Tunisia	Landrace	130	160	0	HR	81,48	0,1	HR	0	HR	0	0	HR	1	3
CItr 3221	Louri AC 8	Tunisia	Landrace	130	155	0	HR	32,72	0,04	HR	0	HR	0	0	HR	1	3
CItr 3222	Louri AC 9	Tunisia	Landrace	130	160	0	HR	148,15	0,19	HR	2	R	324,07	0,21	R	2	1
CItr 3223	Louri AP 2	Tunisia	Landrace	130	145	0	HR	165,43	0,21	R	1	HR	69,14	0,04	HR	1	3
CItr 3225	Louri AP 5	Tunisia	Landrace	130	150	0	HR	16,05	0,02	HR	1	HR	69,14	0,04	HR	1	3
CItr 3231	Louri RP 5	Tunisia	Landrace	130	150	0	HR	48,15	0,06	HR	1	HR	69,14	0,04	HR	1	3
PI 55538	Mahmoudi AC 3	Tunisia	Landrace	130	130	0	HR	165,43	0,21	R	0	HR	0	0	HR	1	3
PI 534368	Mamhoudi	Tunisia	Landrace	130	145	2	R	90,12	0,11	HR	2	R	241,98	0,16	HR	1	3
CItr 3244	Medea AC 2	Tunisia	Landrace	130	145	0	HR	165,43	0,21	R	1	HR	151,23	0,1	HR	1	3

PI 534373	MG 18161	Tunisia	Landrace	130	115	3	MR	48,15	0,06	HR	3	MR	246,3	0,16	HR	1	4
PI 41038	Realforte	Tunisia	Landrace	130	145	0	HR	32,72	0,04	HR	0	HR	0	0	HR	1	3
CItr 6870	Souri	Tunisia	Landrace	130	150	0	HR	146,91	0,19	HR	0	HR	241,98	0,16	HR	1	3
PI 55544	Souri	Tunisia	Landrace	130	140	1	HR	181,48	0,23	R	0	HR	0	0	HR	1	3
PI 41043	Taganrog	Tunisia	Landrace	130	160	0	HR	165,43	0,21	R	1	HR	69,14	0,04	HR	1	3
CItr 15510	Bedi	Tunisia	Landrace	135	130	0	HR	0	0	HR	3	MR	397,53	0,26	R	1	3
PI 306575	D 117	Tunisia	Breeding material	135	130	0	HR	0	0	HR	0	HR	246,3	0,16	HR	1	3
PI 55536	Hamira	Tunisia	Landrace	135	145	3	MR	213,58	0,27	R	0	HR	0	0	HR	1	3
PI 55537	Jenah Rhetifah	Tunisia	Landrace	135	160	0	HR	146,91	0,19	HR	0	HR	0	0	HR	1	3
CItr 3199	Jennah Rhetifa AP 4	Tunisia	Landrace	135	125	0	HR	81,48	0,1	HR	0	HR	0	0	HR	1	3
CItr 15454	1144-86	Tunisia	Landrace	120	115	0	HR	374,07	0,48	MR	4	MS	466,67	0,3	R	2	-
CItr 15452	2222-78	Tunisia	Landrace	125	155	0	HR	692,59	0,88	S	2	R	350	0,23	R	2	1
CItr 15455	2238-82	Tunisia	Landrace	130	135	3	MR	248,15	0,32	R	3	MR	466,67	0,3	R	2	1
CItr 15451	3102-76	Tunisia	Landrace	130	155	0	HR	379,01	0,48	MR	2	R	194,44	0,13	HR	2	-
CItr 15411	3320-18	Tunisia	Landrace	130	150	0	HR	211,11	0,27	R	3	MR	350	0,23	R	2	1
CItr 15450	3320-72	Tunisia	Landrace	140	150	3	MR	248,15	0,32	R	4	MS	466,67	0,3	R	2	1
CItr 15449	3356-74	Tunisia	Landrace	130	145	1	HR	411,73	0,52	MR	3	MR	440,74	0,29	R	2	-
PI 55529	Adjini	Tunisia	Landrace	120	145	0	HR	272,22	0,35	R	3	MR	466,67	0,3	R	2	1
CItr 3138	Adjini AP 1	Tunisia	Landrace	130	170	0	HR	265,43	0,34	R	2	R	246,3	0,16	HR	2	1
CItr 3139	Adjini RC 1	Tunisia	Landrace	130	150	0	HR	313,58	0,4	R	3	MR	483,95	0,31	R	2	1
CItr 15482	Afili Romani	Tunisia	Landrace	130	155	4	MS	181,48	0,23	R	2	R	380,25	0,25	R	2	1
CItr 3143	Agili Glabre RP 1	Tunisia	Landrace	150	145	0	HR	475,31	0,6	MS	1	HR	69,14	0,04	HR	2	-
CItr 15415	Ajili	Tunisia	Landrace	140	140	0	HR	444,44	0,57	MR	2	R	350	0,23	R	2	-
CItr 15432	Ajili	Tunisia	Landrace	100	145	0	HR	348,15	0,44	MR	3	MR	466,67	0,3	R	2	-
CItr 15473	Ajili	Tunisia	Landrace	130	150	0	HR	528,4	0,67	MS	3	MR	466,67	0,3	R	2	-
CItr 15487	Ajili	Tunisia	Landrace	130	145	0	HR	622,22	0,79	MS	3	MR	540,12	0,35	R	2	-
CItr 15488	Ajili	Tunisia	Landrace	130	130	0	HR	344,44	0,44	MR	2	R	358,64	0,23	R	2	-
CItr 15895	Ajili	Tunisia	Landrace	140	165	0	HR	443,21	0,56	MR	3	MR	328,4	0,21	R	2	-

CItr 15477	Arbi	Tunisia	Landrace	130	105	0	HR	409,88	0,52	MR	3	MR	466,67	0,3	R	2	-
CItr 15480	Arbi	Tunisia	Landrace	140	130	0	HR	608,64	0,77	MS	0	HR	337,04	0,22	R	2	-
CItr 15490	Arbi	Tunisia	Landrace	115	155	0	HR	353,7	0,45	MR	3	MR	436,42	0,28	R	2	-
CItr 15491	Arbi	Tunisia	Landrace	120	150	0	HR	396,3	0,5	MR	2	R	246,3	0,16	HR	2	-
CItr 15913	Arbi	Tunisia	Landrace	120	145	0	HR	120,37	0,15	HR	3	MR	466,67	0,3	R	2	1
CItr 3156	Azizi AC 2	Tunisia	Landrace	135	100	0	HR	496,3	0,63	MS	2	R	69,14	0,04	HR	2	-
CItr 3162	Azizi AP 5	Tunisia	Landrace	120	140	0	HR	337,65	0,43	MR	1	HR	69,14	0,04	HR	2	-
CItr 15453	2260-84	Tunisia	Landrace	140	145	4	MS	0	0	HR	4	MS	466,67	0,3	R	1	3
PI 433751	Badri	Tunisia	Cultivar	80	120	0	HR	474,07	0,6	MS	5	S	1464,81	0,95	S	3	-
CItr 15497	6336-8	Tunisia	Landrace	140	135	0	HR	66,67	0,08	HR	2	R	449,38	0,29	R	1	1
CItr 3141	Agili Glabre RC 1	Tunisia	Landrace	140	140	0	HR	214,81	0,27	R	1	HR	69,14	0,04	HR	1	3
CItr 15483	Bedi	Tunisia	Landrace	120	140	0	HR	625,93	0,8	MS	1	HR	241,98	0,16	HR	2	-
CItr 3148	Agili Pubescent AC 2	Tunisia	Landrace	140	135	0	HR	200	0,25	R	0	HR	0	0	HR	1	3
CItr 15512	Agin Sinlika	Tunisia	Landrace	140	125	0	HR	58,02	0,07	HR	0	HR	207,41	0,13	HR	1	3
CItr 3160	Azizi AP 3	Tunisia	Landrace	140	150	0	HR	165,43	0,21	R	3	MR	203,09	0,13	HR	1	1
CItr 15494	Beskri	Tunisia	Landrace	140	145	0	HR	337,65	0,43	MR	3	MR	241,98	0,16	HR	2	-
CItr 15495	Beskri	Tunisia	Landrace	140	145	0	HR	330,86	0,42	MR	3	MR	661,11	0,43	MR	2	-
CItr 15499	Beskri	Tunisia	Landrace	130	150	2	R	309,88	0,39	R	0	HR	203,09	0,13	HR	2	1
CItr 3811	Bidi	Tunisia	Landrace	130	145	0	HR	361,73	0,46	MR	4	MS	449,38	0,29	R	2	-
CItr 3170	Bidi AP 1	Tunisia	Landrace	130	130	0	HR	361,73	0,46	MR	2	R	259,26	0,17	HR	2	-
CItr 3166	Azizi AP 9	Tunisia	Landrace	140	140	0	HR	146,91	0,19	HR	2	R	246,3	0,16	HR	1	1
CItr 3169	Baiada RP 1	Tunisia	Landrace	140	155	0	HR	48,15	0,06	HR	0	HR	69,14	0,04	HR	1	3
CItr 15515	Bedi	Tunisia	Landrace	140	155	2	R	165,43	0,21	R	2	R	69,14	0,04	HR	1	3
CItr 15516	Bedi	Tunisia	Landrace	140	120	NA	NA	0	0	HR	2	R	246,3	0,16	HR	1	3
PI 534344	Bidri	Tunisia	Landrace	140	155	4	MS	884,57	1,13	S	3	MR	756,17	0,49	MR	3	-
CItr 3172	Bidi AP 3	Tunisia	Landrace	140	145	0	HR	48,15	0,06	HR	0	HR	0	0	HR	1	3
PI 55535	Biskri	Tunisia	Landrace	135	140	0	HR	246,91	0,31	R	4	MS	583,33	0,38	R	2	1

CItr 3183	Biskri Pubescent AC 3	Tunisia	Landrace	140	140	0	HR	0	0	HR	0	HR	0	0	HR	1	3
CItr 15461	Frigui	Tunisia	Landrace	140	130	0	HR	237,65	0,3	R	0	HR	0	0	HR	1	3
CItr 3177	Biskri Glabre AP 2	Tunisia	Landrace	150	150	3	MR	692,59	0,88	S	4	MS	630,86	0,41	MR	2	-
PI 191502	Hamira	Tunisia	Landrace	140	145	NA	NA	213,58	0,27	R	0	HR	0	0	HR	1	3
CItr 3196	Hamira AC 4	Tunisia	Landrace	140	140	3	MR	200	0,25	R	0	HR	151,23	0,1	HR	1	1
CItr 3983	ICM 313	Tunisia	Landrace	140	125	4	MS	0	0	HR	0	HR	0	0	HR	1	3
CItr 3984	ICM 314	Tunisia	Landrace	140	145	2	R	0	0	HR	2	R	259,26	0,17	HR	1	3
CItr 3185	Biskri Velu RC 1	Tunisia	Landrace	140	150	1	HR	165,43	0,21	R	3	MR	466,67	0,3	R	2	1
CItr 3200	Jennah Rhetifa AP 9	Tunisia	Landrace	140	140	0	HR	213,58	0,27	R	1	HR	151,23	0,1	HR	1	1
CItr 3206	Jennah Rhetifa RP 5	Tunisia	Landrace	140	150	0	HR	129,63	0,16	HR	2	R	246,3	0,16	HR	1	3
PI 534336	Chili	Tunisia	Landrace	120	130	4	MS	1264,2	1,61	S	3	MR	760,49	0,49	MR	3	-
CItr 3241	Mahmoudi Glabre RP 2	Tunisia	Landrace	140	145	0	HR	146,91	0,19	HR	1	HR	241,98	0,16	HR	1	1
PI 306576	D 240-5-1p-3	Tunisia	Breeding material	100	130	0	HR	487,65	0,62	MS	0	HR	0	0	HR	2	-
CItr 3245	Medea AC 3	Tunisia	Landrace	140	175	0	HR	74,07	0,09	HR	0	HR	0	0	HR	1	3
CItr 3252	Medea AP 6	Tunisia	Landrace	140	165	0	HR	81,48	0,1	HR	0	HR	0	0	HR	1	3
CItr 3187	Derberri AC 1	Tunisia	Landrace	105	135	NA	NA	146,91	0,19	HR	5	S	756,17	0,49	MR	2	-
CItr 3189	Derberri AC 3	Tunisia	Landrace	90	120	0	HR	596,3	0,76	MS	4	MS	544,44	0,35	R	3	-
CItr 3190	Derberri AP 1	Tunisia	Landrace	110	135	3	MR	958,02	1,22	S	4	MS	786,42	0,51	MR	3	-
PI 191634	Sebei	Tunisia	Unknown improvement status	140	130	0	HR	165,43	0,21	R	0	HR	0	0	HR	1	3
PI 55534	Derbessi	Tunisia	Landrace	110	130	3	MR	545,68	0,69	MS	4	MS	617,9	0,4	MR	3	-
PI 191938	Derbessi	Tunisia	Landrace	110	105	NA	NA	591,36	0,75	MS	5	S	933,33	0,61	MS	3	-
CItr 15496	Dhil Bhal Senlikat	Tunisia	Landrace	140	160	4	MS	348,15	0,44	MR	3	MR	717,28	0,47	MR	2	-
CItr 15479	Farine Arbi	Tunisia	Landrace	130	150	0	HR	329,01	0,42	MR	3	MR	466,67	0,3	R	2	-
CItr 15404	Frigui	Tunisia	Landrace	145	135	0	HR	492,59	0,63	MS	1	HR	293,83	0,19	HR	2	-

CItr 15413	Frigui	Tunisia	Landrace	140	130	0	HR	424,07	0,54	MR	4	MS	375,93	0,24	R	2	-
CItr 15458	Frigui	Tunisia	Landrace	130	145	2	R	677,78	0,86	S	3	MR	466,67	0,3	R	2	1
CItr 15459	Frigui	Tunisia	Landrace	130	145	0	HR	387,65	0,49	MR	1	HR	298,15	0,19	HR	2	-
CItr 15462	Frigui	Tunisia	Landrace	140	145	0	HR	671,6	0,85	S	1	HR	151,23	0,1	HR	2	1
CItr 15464	Frigui	Tunisia	Landrace	150	150	0	HR	676,54	0,86	S	2	R	328,4	0,21	R	2	1
CItr 15486	Frigui	Tunisia	Landrace	120	120	NA	NA	760,49	0,97	S	3	MR	328,4	0,21	R	2	1
CItr 15896	Frigui	Tunisia	Landrace	130	140	0	HR	424,07	0,54	MR	3	MR	535,8	0,35	R	2	-
PI 191564	Hamira	Tunisia	Landrace	140	130	NA	NA	165,43	0,21	R	3	MR	518,52	0,34	R	2	1
CItr 3193	Hamira AC 1	Tunisia	Landrace	120	145	0	HR	343,21	0,44	MR	3	MR	466,67	0,3	R	2	-
CItr 3195	Hamira AC 3	Tunisia	Landrace	130	150	2	R	200	0,25	R	2	R	350	0,23	R	2	1
CItr 15420	Hmira	Tunisia	Landrace	110	125	NA	NA	213,58	0,27	R	4	MS	432,1	0,28	R	2	4
CItr 15426	Hmira	Tunisia	Landrace	140	150	4	MS	545,68	0,69	MS	4	MS	466,67	0,3	R	2	-
CItr 15444	Hmira	Tunisia	Landrace	90	125	NA	NA	200	0,25	R	3	MR	466,67	0,3	R	2	4
CItr 15457	Hmira	Tunisia	Landrace	90	140	NA	NA	422,22	0,54	MR	4	MS	432,1	0,28	R	2	-
CItr 15460	Hmira	Tunisia	Landrace	140	155	3	MR	774,69	0,99	S	3	MR	328,4	0,21	R	2	1
CItr 15908	Hmira	Tunisia	Landrace	120	140	NA	NA	474,07	0,6	MS	5	S	280,86	0,18	HR	2	-
CItr 15514	Hmira Makaou	Tunisia	Landrace	140	145	4	MS	329,63	0,42	MR	1	HR	69,14	0,04	HR	2	-
PI 433756	Inrat 69	Tunisia	Cultivar	110	90	4	MS	1792,59	2,28	S	1	HR	868,52	0,57	MR	3	-
PI 221407	Jenah Retifah	Tunisia	Landrace	125	130	3	MR	466,67	0,3	R	2	R	103,7	0,13	HR	2	1
CItr 3201	Jennah Rhetifa AP 10	Tunisia	Landrace	150	155	2	R	358,02	0,46	MR	4	MS	328,4	0,21	R	2	-
CItr 3198	Jennah Rhetifa AP 2	Tunisia	Landrace	130	135	1	HR	361,73	0,46	MR	2	R	298,15	0,19	HR	2	-
CItr 3202	Jennah Rhetifa RP 1	Tunisia	Landrace	120	145	2	R	632,72	0,81	S	3	MR	535,8	0,35	R	2	1
CItr 3205	Jennah Rhetifa RP 4	Tunisia	Landrace	140	155	0	HR	538,27	0,69	MS	4	MS	626,54	0,41	MR	2	-
CItr 3832	Khetifa	Tunisia	Landrace	125	130	3	MR	520,37	0,66	MS	0	HR	0	0	HR	2	-
CItr 3209	Lebei Pubescent Bas AP 2	Tunisia	Landrace	120	135	2	R	329,63	0,42	MR	3	MR	466,67	0,3	R	2	-
CItr 3230	Louri RP 4	Tunisia	Landrace	130	145	4	MS	422,22	0,54	MR	2	R	350	0,23	R	2	-

CItr 3809	Mahmoudi	Tunisia	Landrace	130	150	3	MR	377,78	0,48	MR	0	HR	69,14	0,04	HR	2	-
CItr 3824	Mahmoudi	Tunisia	Landrace	130	160	3	MR	377,78	0,48	MR	1	HR	151,23	0,1	HR	2	-
CItr 15501	Mahmoudi	Tunisia	Landrace	120	145	0	HR	248,15	0,32	R	0	HR	241,98	0,16	HR	2	1
PI 41045	Mahmoudi	Tunisia	Landrace	130	140	3	MR	304,32	0,39	R	2	R	350	0,23	R	2	1
CItr 3236	Mahmoudi Glabre AP 2	Tunisia	Landrace	115	135	4	MS	448,15	0,57	MR	2	R	272,22	0,18	HR	2	-
PI 150381	Mahmoudi Hybrid	Tunisia	Breeding material	110	120	1	HR	474,07	0,6	MS	1	HR	69,14	0,04	HR	2	-
CItr 3117	Matmata	Tunisia	Landrace	110	110	0	HR	NA	NA	NA	4	MS	401,85	0,26	R	-	-
CItr 3243	Medea AC 1	Tunisia	Landrace	150	155	0	HR	329,63	0,42	MR	4	MS	375,93	0,24	R	2	-
CItr 3142	Agili Glabre RC 2	Tunisia	Landrace	150	140	0	HR	66,67	0,08	HR	0	HR	0	0	HR	1	3
CItr 3144	Agili Glabre RP 2	Tunisia	Landrace	150	150	0	HR	170,99	0,22	R	0	HR	0	0	HR	1	3
CItr 3158	Azizi AP 1	Tunisia	Landrace	150	140	0	HR	255,56	0,33	R	0	HR	69,14	0,04	HR	2	1
CItr 3248	Medea AP 1	Tunisia	Landrace	160	165	0	HR	165,43	0,21	R	2	R	203,09	0,13	HR	2	1
CItr 3256	Medea AP 10	Tunisia	Landrace	130	155	0	HR	165,43	0,21	R	2	R	324,07	0,21	R	2	1
CItr 3224	Louri AP 3	Tunisia	Landrace	150	155	0	HR	81,48	0,1	HR	0	HR	0	0	HR	1	3
CItr 3251	Medea AP 4	Tunisia	Landrace	140	160	0	HR	337,65	0,43	MR	0	HR	203,09	0,13	HR	2	-
CItr 3226	Louri AP 6	Tunisia	Landrace	150	160	0	HR	48,15	0,06	HR	1	HR	164,2	0,11	HR	1	3
CItr 3255	Medea AP 9	Tunisia	Landrace	140	150	0	HR	165,43	0,21	R	3	MR	362,96	0,24	R	2	1
CItr 3258	Medea RP 1	Tunisia	Landrace	150	150	0	HR	90,12	0,11	HR	3	MR	587,65	0,38	R	2	1
PI 174671	Mekki	Tunisia	Landrace	130	165	3	MR	725,93	0,47	MR	3	MR	202,47	0,26	R	2	-
CItr 3229	Louri RP 3	Tunisia	Landrace	150	155	0	HR	165,43	0,21	R	2	R	69,14	0,04	HR	1	3
PI 55541	Mekki	Tunisia	Landrace	135	149	0	HR	198,77	0,25	R	3	MR	350	0,23	R	2	1
PI 55542	Mekki	Tunisia	Landrace	135	145	0	HR	165,43	0,21	R	4	MS	466,67	0,3	R	2	1
PI 191913	Mahon Rijo	Tunisia	Unknown improvement status	150	150	0	HR	0	0	HR	2	R	64,2	0,08	HR	1	3
CItr 15509	Melange	Tunisia	Landrace	135	140	1	HR	309,88	0,39	R	3	MR	466,67	0,3	R	2	1
PI 55540	Medea	Tunisia	Landrace	150	160	0	HR	106,17	0,14	HR	2	R	285,19	0,19	HR	1	3
PI 534353	MG 18141	Tunisia	Landrace	150	155	2	R	344,44	0,44	MR	1	HR	259,26	0,17	HR	2	-

CItr 3250	Medea AP 3	Tunisia	Landrace	150	165	0	HR	213,58	0,27	R	0	HR	151,23	0,1	HR	2	1
PI 534356	MG 18144	Tunisia	Landrace	115	115	4	MS	153,7	0,2	HR	2	R	548,77	0,36	R	2	4
PI 534357	MG 18145	Tunisia	Landrace	140	125	4	MS	476,54	0,61	MS	2	R	479,63	0,31	R	2	-
PI 534358	MG 18146	Tunisia	Landrace	140	145	4	MS	213,58	0,27	R	3	MR	380,25	0,25	R	2	1
PI 534360	MG 18148	Tunisia	Landrace	130	150	3	MR	294,44	0,37	R	3	MR	527,16	0,34	R	2	1
PI 534361	MG 18149	Tunisia	Landrace	145	140	4	MS	687,04	0,87	S	2	R	591,98	0,39	R	2	1
PI 534362	MG 18150	Tunisia	Landrace	145	145	4	MS	328,4	0,42	MR	3	MR	466,67	0,3	R	2	-
PI 534365	MG 18153	Tunisia	Landrace	130	140	5	S	522,22	0,66	MS	3	MR	246,3	0,16	HR	2	-
CItr 6880	Morocco	Tunisia	Landrace	130	125	4	MS	337,65	0,43	MR	2	R	445,06	0,29	R	2	-
PI 41037	Namira	Tunisia	Landrace	130	160	NA	NA	165,43	0,21	R	4	MS	449,38	0,29	R	2	1
CItr 15493	Oued Kebir	Tunisia	Landrace	130	130	0	HR	270,99	0,34	R	3	MR	246,3	0,16	HR	2	1
CItr 3260	Real Sorte AC 2	Tunisia	Landrace	130	145	0	HR	524,07	0,67	MS	1	HR	319,75	0,21	R	2	-
PI 55546	Sbei	Tunisia	Landrace	110	130	2	R	392,59	0,5	MR	4	MS	449,38	0,29	R	2	-
PI 41042	Sbei Smooth	Tunisia	Landrace	120	150	3	MR	392,59	0,5	MR	1	HR	224,69	0,15	HR	2	-
CItr 15506	Sinlikat	Tunisia	Landrace	130	155	0	HR	277,78	0,35	R	1	HR	241,98	0,16	HR	2	1
CItr 3181	Biskri Pubescent AC 1	Tunisia	Landrace	160	165	1	HR	0	0	HR	1	HR	69,14	0,04	HR	1	3
CItr 15519	Ward Bled	Tunisia	Landrace	140	130	2	R	213,58	0,27	R	2	R	328,4	0,21	R	2	1
PI 189772	Ble Dur 116	Tunisia	Cultivar	160	140	0	HR	49,38	0,06	HR	0	HR	0	0	HR	1	3
CItr 3246	Medea AC 4	Tunisia	Landrace	170	145	0	HR	165,43	0,21	R	1	HR	69,14	0,04	HR	1	3
PI 174664	Kirmize	Turkey	Unknown improvement status	110	130	4	MS	298,15	0,19	HR	NA	NA	64,2	0,08	HR	1	4
PI 245746		Turkey	Landrace	110	130	2	R	246,3	0,16	HR	3	MR	101,23	0,13	HR	1	4
PI 185083	Calibasan	Turkey	Landrace	135	150	0	HR	0	0	HR	0	HR	140,74	0,18	HR	1	3
PI 264255	Akbasak	Turkey	Unknown improvement status	130	135	0	HR	570,37	0,37	R	2	R	195,06	0,25	R	2	1
PI 266979	Akbasak	Turkey	Unknown improvement status	130	115	0	HR	0	0	HR	1	HR	NA	NA	NA	-	-

PH: plant height; SD2019: accessions evaluated at seedling stage during 2018-2019 cropping season; SD2017: accessions evaluated at seedling stage during 2016-2017 cropping season; Adult: accessions evaluated at adult stage; HR: highly resistant; R: resistant; MR: moderately resistant; MS: moderately susceptible; S: susceptible; NA: Missing data.

Table S2. Ranking of mean area under disease progress curve (AUDPC) and plant height (PH) of checks (Karim, Salim, and Nasr) evaluated in the field during two seasons (2016-2017 and 2018-2019) against *Z. tritici*

**Cropping season**	**2016-2017**			**2018-2019**		
	**Karim**	**Nasr**	**Salim**	**Karim**	**Nasr**	**Salim**
Mean AUDPC	1536.831	820.679	651.172	785.665	597.194	507.561
SD AUDPC	138.468	100.699	96.066	83.985	72.470	75.591
AUDPC range	1313.58-1858.02	669.753-989.506	410.494-795.062	646.296 - 903.703	503.704 - 696.296	402.469 - 658.025
range PH	75-95	80-110	80-115	-	-	-
Mean PH	90	94.285	94	-	-	-
SD PH	5.028	5.886	7.588	-	-	-

SD: Standard deviation; PH: Plant height in cm.
